# Coordinative Alignment of Chiral Molecules to Control over the Chirality Transfer in Spontaneous Resolution and Asymmetric Catalysis

**DOI:** 10.1038/s41598-017-15780-0

**Published:** 2017-11-13

**Authors:** Zhengqiang Xia, Xu Jing, Cheng He, Xiaoge Wang, Chunying Duan

**Affiliations:** 1 State Key Laboratory of Fine Chemicals, Dalian University of Technology, Dalian, 116024 China; 2 College of Zhang Dayu, Dalian University of Technology, Dalian, 116024 China; 3 Collaborative Innovation Center of Chemical Science and Engineering, Tianjin, 300071 China

## Abstract

The production and availability of enantiomerically pure compounds that spurred the development of chiral technologies and materials are very important to the fine chemicals and pharmaceutical industries. By coordinative alignment of enantiopure guests in the metal‒organic frameworks, we reported an approach to control over the chirality of homochiral crystallization and asymmetric transformation. Synthesized by achiral triphenylamine derivatives, the chirality of silver frameworks was determined by the encapsulated enantiopure azomethine ylides, from which clear interaction patterns were observed to explore the chiral induction principles. With the changing of addition sequence of substrates, the enantioselectivity of asymmetric cycloaddition was controlled to verify the determinant on the chirality of the bulky MOF materials. The economical chirality amplification that merges a series of complicated self-inductions, bulk homochiral crystallization and enantioselective catalysis opens new avenues for enantiopure chemical synthesis and provides a promising path for the directional design and development of homochiral materials.

## Introduction

Chirality is an eminent feature of nature, plays an indispensable role in many fields, including pharmaceutics, industrial chemicals and materials science^1–4^. Of these so-called chiral technologies that exert the ultimate control over a chemical reaction by directing the enantioselectivity^5–8^, heterogeneous asymmetric catalysis is promising because it allows the production and ready separation of large quantities of chiral product using small quantities of catalyst^9,10^. Advances in these fields have led to the emergence of homochiral metal‒organic frameworks (MOFs) that imposes size, shape and conformational restrictions *via* fine-tuned pores and regularly ordered chiral functionalities^11–16^ with enhanced enantioselective functions and new processing options. In particularly, the reversibility of the coordination assembly and the rich chiral configurations around metal nodes provide an attractive approach to achieve homochiral frameworks *via* spontaneous resolution using achiral precursors^17–21^.

Notably, this type of spontaneous resolution that dominated by thermodynamic factors is often uncontrollable and must be based on the statistical fluctuation of the initial nucleation event^22–24^. Chiral induction, as an effective way to overcome such hurdles, is generally necessary to incorporate chiral interactions between adducts and host frameworks for directing the absolute bulk chirality and accomplish chiral amplification during crystallization^25–29^. The crystalline characteristics of frameworks that depicted the precise patterns of these interactions are expected to provide a new platform to explore the principles and influence factors associated with the chiral induction during the assembly processes^30,31^. Difficulties to stabilize the intermediates with needed enantioselective performances are maninly attributed to the intrinsic removability of chiral templates during the crystallization and the cooperativity of the weak interactions between the chiral inducers and the host frameworks^32,33^.

**Figure 1:**
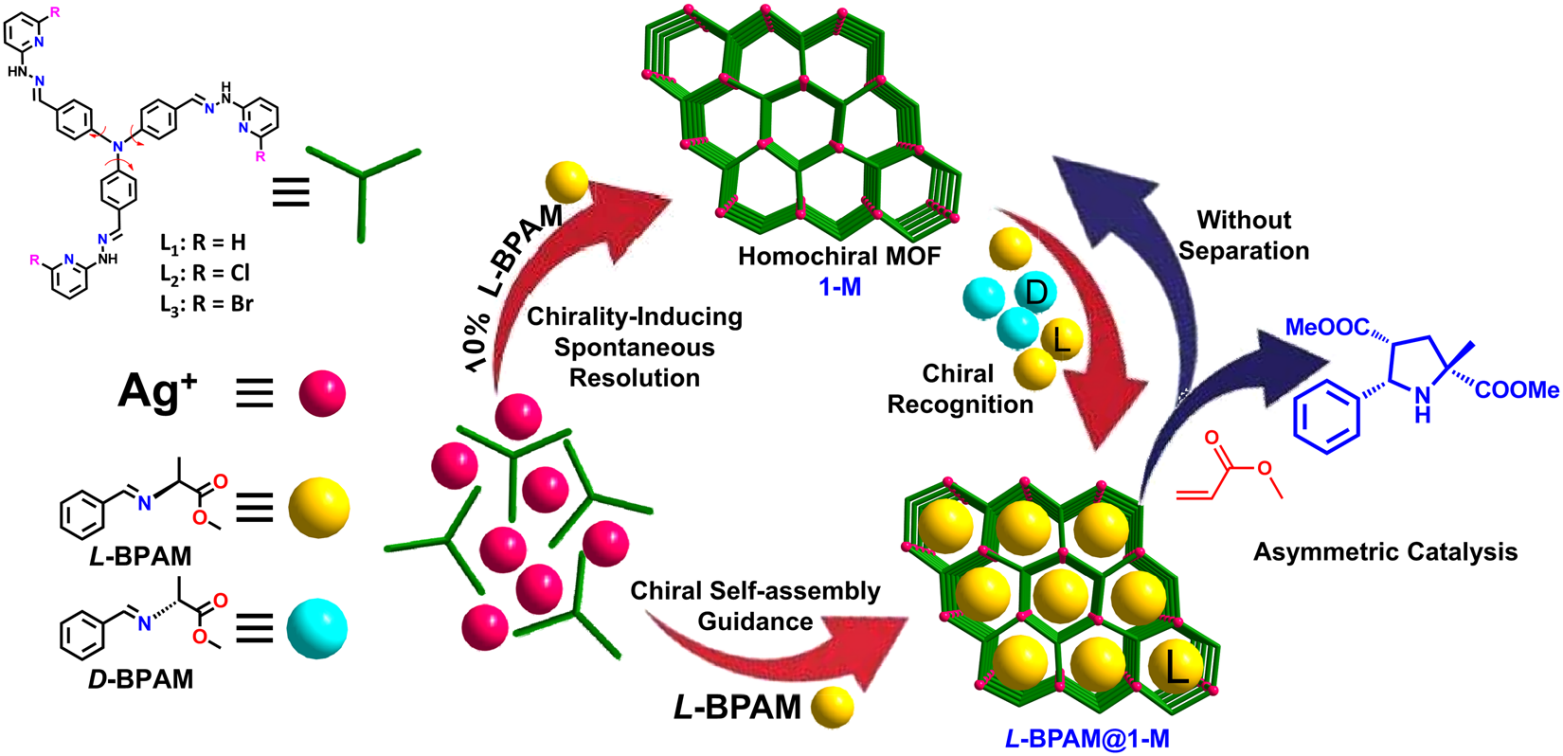
Schematic view of the scientific concept of this paper. Persective view of the chirality-directed spontaneous resolution of the silver frameworks and the asymmetric catalysis by employing an enantiopure substrate at different stages of the whole chiral transformation.

By incorporating ligands with atropisomeric chirality to translate chiralities between metal ions, we have explored economical chirality amplification approach to construct silver frameworks *via* spontaneous resolution for the asymmetric chemical transformation^34^. Using chiral azomethine ylide substrates to direct the absolute chirality of this kind of silver framework, herein, we try to stabilize the intermediates to explore the chiral induction principles and relevant influence factors during the crystallization processes, and further to regulate the chirality of the materials and asymmetric 1,3-dipolar cycloaddition transformation. We envisioned that the presence of enantiopure substrates would cause the observed interactions between substrates and host frameworks to respond to the chirality transfer from the origin chirality of substrate to the conformational chirality of silver(I) centers. The well-matched chiral interactions should be identified to the special pair of chiral substrate and host framework with directed chirality, which lead to symmetry breaking in competitive crystallization. The modified systems possibly allow the detailed exploration of mechanism of chiral self-assembly and interaction patterns between the chiral agents and frameworks, which directly decides the chirality of the bulky materials and further reflects the chiral self-assembly rules of the materials consisting of completely achiral fragments. It is also postulated that the model asymmetric cycloaddition reactions would further give opportunity to identify the efficiency of spontaneous resolution and asymmetric catalysis by employing an enantiopure substrate at different steps of the whole chiral transformation (Fig. 1).

## Results

**Figure 2:**
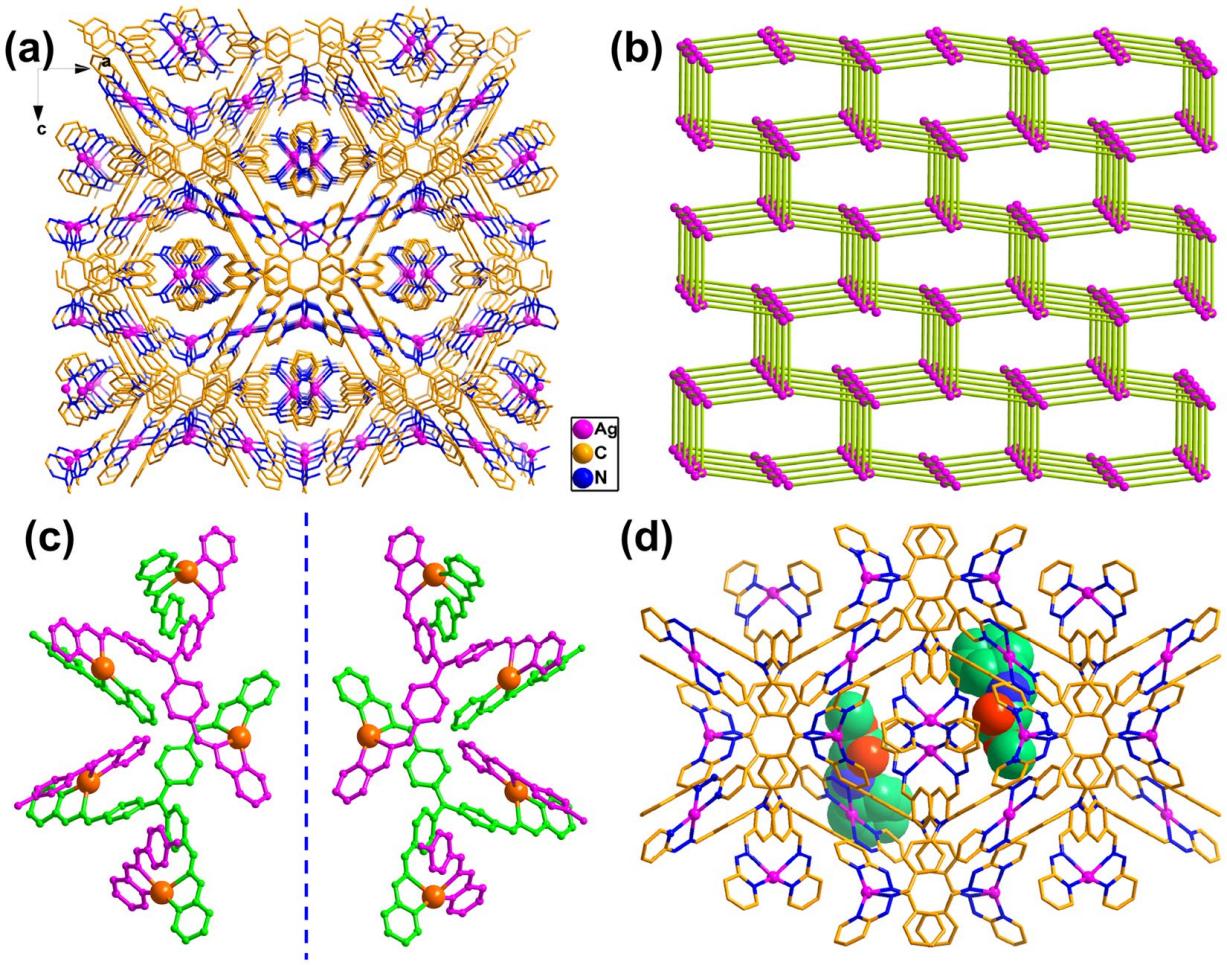
Structures of **1** and ***L*** **-BPAM@1-** ***M***. (**a**) 3D framework of **1** without H atoms, counter ions and solvent molecules. (**b**) Perspective view of the (10,3)-*a* topology of **1**, where pink spheres represent the center of the trisphenylamine moiety. (**c**) Mirror image structures of **1-** ***M*** (left) and **1-** ***P*** (right). (**d**) Crystal structure of **1-** ***M*** with encapsulated ***L*** **-BPAM** molecules.

The propeller-like ligand tris(4-(2-pyridin-2-ylhydrazono)phenyl)amine (**L**_**1**_) was synthesized in 90% yield from a Schiff-base reaction with 2-hydrazinopyridine and tris(4-formylphenyl)amine (molar ratio = 3:1)^35,36^. Diffusing a chloroform solution of the ligand into an acetonitrile solution of silver(I) salt yielded the three-dimensional framework **1** in a yield of 46% (Figure S1). Single-crystal X-ray diffraction revealed that **1** crystallizes in a space group *I*2_1_3. The silver(I) ion that located at the 2_1_ axis is coordinated by four N atoms from two different pyridine-imine chelators, giving rise to the conformational chirality. The ligand positioned at a three-fold axis bridges three silver(I) ions and is locked in an atropisomerically chiral conformation. This special conformation transmits the original chirality of one silver(I) center to another metal center that it bridges, forming a helical species (Figures S3–S8). The chirality of silver(I) centers constrains two coordinated ligands to adopt the same atropisomeric chirality, eventually leading to the formation of a homochiral framework (Fig. 2a). If the ligands are defined as three connected nodes and the silver(I) ions as directional linkers, the homochiral framework can be described as an intrinsic chiral (10,3)-*a* net (Fig. 2b) that represented by the prototypical structures of SrSi_2_ and *a*-Hg_3_S_2_Cl_2_^37,38^. Such a special architecture with intrinsic chirality and catalytic sites allows the silver-based material promisingly used as a heterogeneous asymmetric catalyst.

**Figure 3:**
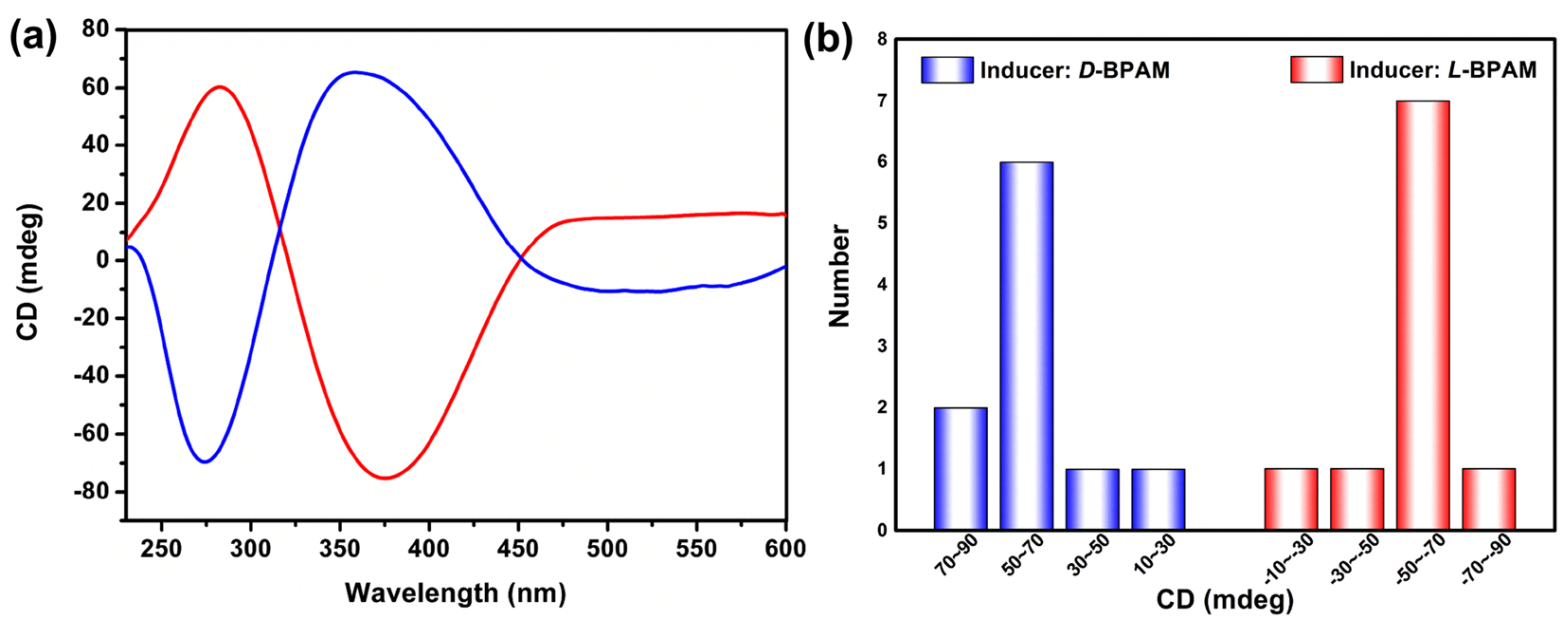
Chirality characterizations of the induced-formed **1-** ***M*** and **1-** ***P*** catalysts. (**a**) CD spectra of bulk crystalline solids of **1-** ***M*** (blue) and **1-** ***P*** (red), showing the opposite Cotton effects of the two compounds. (**b**) CD normal distribution of 10 bulk samples of **1** from 10 independent crystallizations induced by ***D*** **-BPAM** or ***L*** **-BPAM**.

Solid-state circular dichroism (CD) spectra for 20 bulk samples of **1** from 20 independent crystallizations either exhibited a positive Cotton effect at approximately 375 nm and a negative Cotton effect at approximately 280 nm or exhibited the opposite signals (Figure S19), suggesting the formation of either excess *M* or *P* enantiomers (denoted **1-** ***M*** and **1-** ***P***) during the crystallization, respectively (Figs 2c and 3a). This nonzero enantiomeric excess that occurred without the introduction of any chiral agents indicated the characteristic spontaneous resolution and the formation of a homochiral framework^39^. Single-crystal X-ray diffraction data of six randomly selected **1** crystals from a single crystallization revealed an almost equal distribution of ***P*** and ***M*** configurations, with all Flack parameters being nearly zero. (Figure S20 and Table S3). Solids of **1** (3 mol%) randomly selected from five isolated crystallization batches were employed to catalyze the cycloaddition between (*S*,*E*)-methyl-2-(benzylideneamino)propanoate (***L*** **-BPAM**) (Figures S2 and S39) and methyl acrylate. After 24 h of reaction in the presence of 10 mol% of Et_3_N, the system yielded only the *endo* products with a conversion range of 82~88% (entry 1, Table 1). Such excellent diastereoselectivity might be due to the spatial confinement effect of special helical channels within the Ag-MOFs. Meanwhile, an *ee* that varied from −19 to 33%, was also observed (Figure S52).

**Table 1:** 1,3-dipolar cycloaddition of *a*-amino ester Schiff bases with methyl acrylate.


**Substrate A:** ***L*** **-BPAM**					**Substrate A:** ***D*** **-BPAM**				
**Entry**	**Catalyst** ^***a***^	**CD**_***θ*=375**_ **/mdeg**	**Conversion** ^***b***^	***ee*** ^***c***^	**Entry**	**Catalyst** ^***a***^	**CD**_***θ*=375**_ **/mdeg**	**Conversion** ^***b***^	***ee*** ^***c***^
1	**1**	18.7	85%	16%	2	**1**	24.3	84%	20%
		−23.7	82%	−19%			−32.6	86%	−32%
		10.4	85%	9%			−17.4	87%	−12%
		−11.6	88%	−8%			−24.5	85%	−19%
		36.1	84%	33%			28.7	88%	29%
3	**1-** ***M***	57.6	88%	89%	4	**1-** ***M***	57.6	82%	88%
		62.3	85%	92%			62.3	84%	92%
		60.2	87%	91%			60.2	83%	90%
		56.6	86%	88%			56.6	84%	87%
		58.1	83%	90%			58.1	86%	89%
5	**1-** ***P***	−62.1	85%	−89%	6	**1-** ***P***	−62.1	89%	−88%
		−63.7	82%	−90%			−63.7	84%	−91%
		−58.5	86%	−87%			−58.5	86%	−86%
		−64.3	88%	−93%			−64.3	87%	−92%
		−60.4	84%	−88%			−60.4	83%	−89%
7	**1** ^*d*^	**—**	92%	44%	8	**1** ^*d*^	**—**	92%	−45%
			88%	49%				91%	−43%
			87%	42%				94%	−37%
			90%	44%				90%	−49%
			93%	41%				93%	−41%

^a^3 mol% (per silver atom) of the catalyst was used. ^b^The conversions were determined from the ^1^H NMR spectra of the crude products. ^c^The *ee* values were determined by HPLC. ^d^Five parallel tandem experiments containing homochiral crystalization and asymmetric catalysis.

Interestingly, the solid-state CD signals of these solids from five isolated crystallization batches were also random but positively linear correlated with the enantiomeric excess (*ee*) of the cycloaddition adduct (Figure S21). Crystals with the ***M*** configuration led to the (2 *R*,4 *R*,5 *S*)-isomer, whereas those with the ***P*** configuration tended to yield the other isomer. Because the substrate activation for the [3 + 2] cycloaddition reaction is a racemization process, the randomly distributed *ee* values of the product are postulated to be related to the randomly distributed chirality of the framework. To illustrate, an achiral substrate, *N*-(phenylmethylene)glycine ethyl ester (**PGE**) (Figure S41) was employed, randomly distributed *ee* values (−45 to 38%) of the *endo* cycloadduct were detected using crystals of **1** from the isolated crystallization batches as the catalysts (3 mol%) under the same conditions (Table S10, Figures S54 and S47–S51). We thus postulate that the chirality or configuration of the product is not influenced by the chirality of the substrate, but is attributed to the chirality of the catalysts. When (*R*,*E*)-methyl-2-(benzylideneamino)propanoate (***D*** **-BPAM**) was used as a substrate (Figure S40), similar results (only *endo* producrs, average conversion: 86% and *ee*: −32% to 29%) were also obtained (entry 2, Table 1 and Figures S22 and S53). These catalytic results suggested that the spontaneous resolution produced a randomness of chirality distribution of the chiral frameworks. It is thus necessary for the further enantioselective application to modify the spontaneous resolution process and direct the chirality of the MOF-based catalysts.

**Figure 4:**
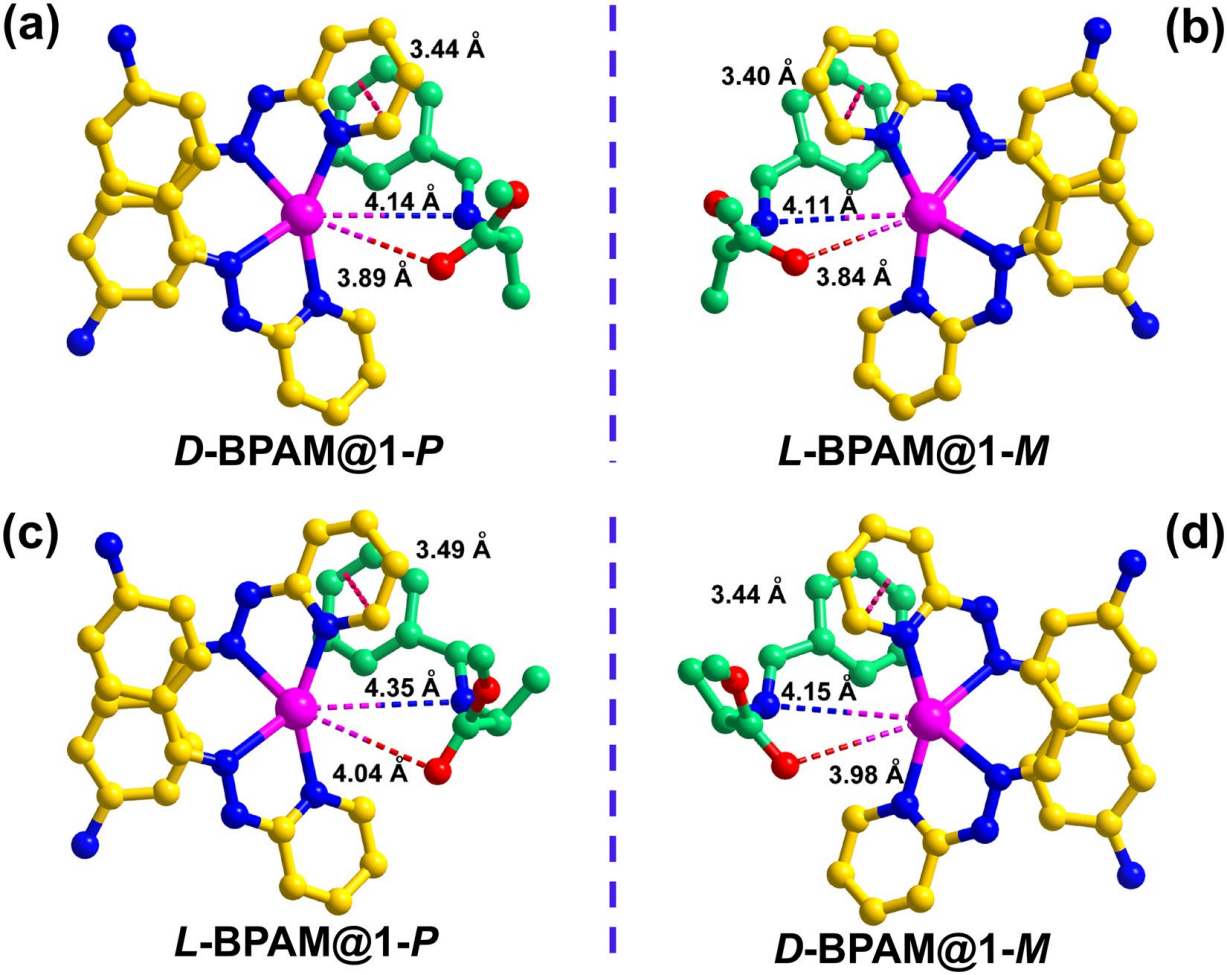
Structures of substrates incorporated catalysts. Intermolecular interactions between **1-** ***P*** and ***D*** **-BPAM** (**a**), **1-** ***M*** and ***L*** **-BPAM** (**b**), **1-** ***P*** and ***L*** **-BPAM** (**c**), and **1-** ***M*** and ***D*** **-BPAM** (**d**).

Many experiments have demonstrated that employing a chiral induction agent (CIA) to direct the homochiral crystallization process is a powerful strategy for achieving homochiral bulk solids^29^, within which the inducer transfers the chirality through the intermolecular chiral interactions. Substrate uptake studies that soaked crystals of **1** in a solution containing ***L*** **-BPAM** or ***D*** **-BPAM** was expected to give more internal chemical information. Fortunately, the crystal forms of all crystals were maintained after 24 h of immersion and the single-crystal structural analyses revealed the maintenance of the space group and cell dimensions (Fig. 2d and Figures S15–S18). As shown in Fig. 4b, the crystal structure of **1-** ***M*** with impregnated ***L*** **-BPAM** reactants (***L*** **-BPAM@1-** ***M***) exhibited that the separations between the carbonyl oxygen and imine nitrogen of ***L*** **-BPAM** and the silver(I) centre were 3.84 Å and 4.11 Å, respectively, and a strong π-π stacking interaction (Cg…Cg = 3.40 Å) occurred between the benzene of ***L*** **-BPAM** and the pyridine of **1-** ***M***. It is suggested that an interplay existed when an intermediate was generated through the chelation of the imine nitrogen and carbonyl oxygen donors, which benefited the substrate activation and the chirality transfer from ***L*** **-BPAM** to the silver centers. While in enantiomorph **1-** ***P*** with absorbed ***L*** **-BPAM** molecules (***L*** **-BPAM@1-** ***P***) (Fig. 4c), an enlarged Ag···N and Ag···O separation around the silver centers (4.35 Å and 4.04 Å, respectively) and a weakened π-π stacking interaction between **BPAM** and the catalyst (Cg…Cg = 3.49 Å) were observed. A similar situation was also found in the crystals soaked in solution of ***D*** **-BPAM** (***D*** **-BPAM@1-** ***P*** and ***D*** **-BPAM@1-** ***M***), with the Ag···N and Ag···O distances between ***D*** **-BPAM** and the silver(I) center in ***D*** **-BPAM@1-** ***P***, 4.14 Å and 3.89 Å, respectively, shorter than those in ***D*** **-BPAM@1-** ***M*** (Fig. 4a,d). These chiral interactions between **1** and **BPAM** revealed that the enantiopure **BPAM** molecules have the potential to drive the chiral information transfer from the **BPAM** to the nucleus of the framework.

By adding 10 mol% of ***L*** **-BPAM** to the interlayer of each crystallization system of **1**, the single-crystal X-ray diffraction data of six randomly and consecutively selected crystals obtained from a single crystallization revealed that each individual crystal was homochiral (all Flack parameters were nearly zero) with an ***M***-configuration of the silver(I) center, featuring the same *I*2_1_3 space group and similar lattice parameters to those of **1** (Figure S23 and Table S4). The CD spectra for 10 sets of crystallizations of bulk crystals showed positive Cotton effects at approximately 375 nm and negative Cotton effects at approximately 280 nm (Fig. 3b and Figure S24), which suggested the excellent inducing-resolution ability of the chiral **BPAM** substrate molecules. **1-** ***M*** crystals from five parallel crystallization batches with the similar CD signals were used as catalyst for the same [3 + 2] cycloaddition reaction between ***L*** **-BPAM** and methyl acrylate under aforementioned reaction conditions (Figure S25), which resulted in the *endo* product with an average conversion of 86% and a high *ee* range of 88~92% (entry 3, Table 1 and Figures S42–S46). When ***D*** **-BPAM** was used as substrate, **1-** ***M*** crystals from the same five crystallizations gave an average conversion of 84% and an *ee* range of 87~92% (entry 4, Table 1). The comparable and identical enantioselectivity suggested that the chirality of the cycloaddition adducts had nothing to do with the chirality of the **BPAM** substates but depended on the handness of the MOF materials (Figures S55 and S56), and at the same time the similar enantioselectivity corresponding to similar CD singals further confirmed the good direction of the ***L*** **-BPAM** molecules on the chirality of the MOF materials.

When the chiral inducers were replaced by 10 mol% of ***D*** **-BPAM**, the CD spectra for 10 sets of crystallizations of the produced bulk crystals all showed the opposite CD signals to those of **1-** ***M*** (Fig. 3b and Figure S27), and the six randomly and consecutively selected crystals from the a single crystallization all featured the ***P***-configuration of silver(I) ion, further verifying the chiral characteristics of the produced **1-** ***P*** crystals (Figure S28 and Table S5). These results demonstrated that the enantiopure **BPAM** can be regarded as real chiral induction agents. As can be expected, the [3 + 2] cycloaddition catalyzed by **1-** ***P*** crystals from five parallel crystallizations with the substrate of ***L*** **-BPAM** (Figure S26), gave the products with the similar conversion range of 82~88% but opposite enantioselectivity ranging from −87% to −93% under the optimum conditions (entry 5, Table 1). With substituting the ***L*** **-BPAM** substrate with ***D*** **-BPAM**, **1-** ***P*** crystals from the same five crystallizations still yielded the similar catalytic performances (*endo* products with an average conversion of 86% and the average *ee* value of −89%) (entry 6, Table 1). These comparable catalytic conversions but opposite enantioselectivity between **1-** ***M*** and **1-** ***P*** suggested that the origin chirality of ***L*** **-BPAM** and ***D*** **-BPAM** was efficiently transferred to the generated **1-** ***M*** and **1-** ***P*** MOF materials during the directed spontaneous resolution processes, respectively (Figures S57 and S58).

From a mechanistic point of view, the transition from an achiral or a conglomerate state to a homochiral or an enantioenriched state could be interpreted as a spontaneous symmetry breaking, in which the secondary crystal nuclei of the same configuration as the parent crystal are rapidly cloned, while competitive crystallization of the opposite enantiomer is suppressed, thus yielding chiral amplification and production of enantiopure crystals^40^. So the stronger interactions between the substrate and silver center in ***L*** **-BPAM@1-** ***M*** mean the more matching chiral environments of them than those in the structure of ***L*** **-BPAM@1-** ***P***. In the presence of ***L*** **-BPAM**, the interactions makes the formation of ***M*** configuration superior to that of ***P*** configuration of silver(I) centres in the competitive crystallization. The chirality stored was then transferred to the mother nucleus of **1-** ***M*** crystals preferentially through these well-merged interactions, which is responsible for the symmetry breaking of **1-** ***M*** in the presence of ***L*** **-BPAM**. For the same reason, the stored chirality of ***D*** **-BPAM** tends to be transferred to the mother nucleus of **1-** ***P***. Apparently, the acquirement of the host-guest chemical interactions and detailed chiral information *via* coordinative alignment of potential CIAs into chiral crystalline materials is an effective way to assess and filtrate the valid CIAs for inducing homochiral spontaneous resolution and chiral amplification, which developed a new approach to achieve the directional preparation of homochiral MOF materials.

Because the similar but opposite enantioselectivity results were derived from the opposite chirality characteristics of induced MOF catalysts, it only indirectly reflected the induction (producing homochiral **1-** ***M*** or **1-** ***P*** materials) and catalytic results but did not clearly present the induction and catalysis processes. Considering that the enantiopure **BPAM** molecules are both CIAs and the reaction substrates, a designed tandem experiment by adding reaction stoichiometric **BPAM** prior to crystallization was performed to better illustrate the spontaneous resolution and asymmetric catalysis processes. This tandem process not only avoided the separation and removal of the generated MOF crystals but also provided the possibility to obtain enantiopure product without introducing any additional chiral agents to direct the absolute chirality of the product. Meanwhile, the CIAs left in the solution do not influence the catalytic transformation.

In this case, the reaction stoichiometric ***L*** **-BPAM** was added prior to the crystal growth, after complete crystallization, the other substate methyl acrylate and 10% Et_3_N were then added to the mixture and was stirred for 24 h. Surprisingly, five parallel abovementioned tandem experiments gave only *endo* products with decent conversions ranging from 87 to 93% and identical enantioselectivity (*ee*) ranging from 41 to 49% (entry 7, Table 1), and the (2 *R*,4 *R*,5 *S*)-isomer of the cycloadduct was dominant (Figure S59). Similarly, when ***D*** **-BPAM** was added prior to the parallel spontaneous resolution systems of **1** under the same reaction conditions, five parallel experiments produced similar conversions (90~94%), but with completely opposite *ee* values ranging from −37 to −49% (entrie 8, Table 1) (Figure S60). The orderly distributed enantio excess value of the adduct (all possess positive or negative *ee*) demonstrated that the chirality of the cycloaddition adducts is derived from and fully determined by the chirality of the **BPAM** substrates, which is completely different from the previous conclusions that the chirality of product has nothing to do with the **BPAM** substrates, wherein the **BPAM** substrates were added after crystalization.

**Figure 5:**
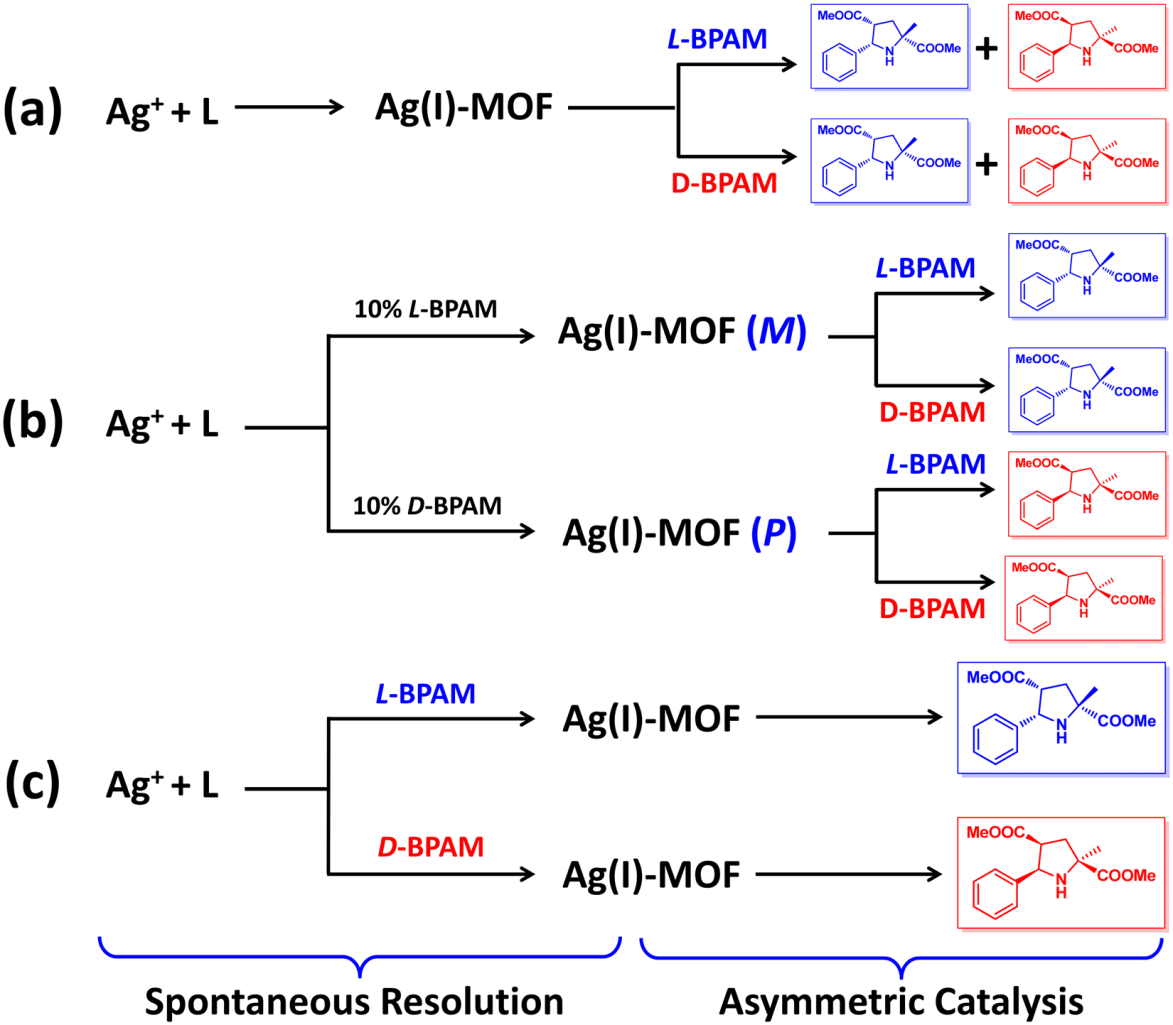
Different processes of catalysts assembly and asymmetric cycloaddition transformations. (**a**) Ag(I)-MOFs formed *via* the spontaneous crystallization were used for the asymmetric 1,3-dipolar cycloaddition, showing the random chirality of the product. (**b**) Ag(I)-MOFs formed *via* the induction crystallization were used for the asymmetric 1,3-dipolar cycloaddition, showing the catalyst-determined chirality of the product. (**c**) The tandem process of spontaneous resolution and the asymmetric 1,3-dipolar cycloaddition, showing the substrate-determined chirality of the product.

Obviously, the alteration of the addition sequence of the homochiral substrates greatly changed the enantioselectivity of the cycloaddition product, and which was exactly based on the accurate understanding of the different chiral interactional ways between the **BPAM** and MOF materials (Fig. 5). Moreover, the formation of the enantioenriched products exhibited that the homochiral **BPAM** molecules were able to bond back smoothly to the silver(I) sites after the chirality-induced crystallization. It was postulated that the chirality of the MOF materials was directed by the chirality of the substrates, the MOFs further duplicated the stored chirality to control the conformation of the active intermediates and the cycloaddition product. This consecutive chirality transfer provided a fresh perspective on the relationship among the chiral origin, the chirality amplification and the asymmetric catalysis.

**Figure 6:**
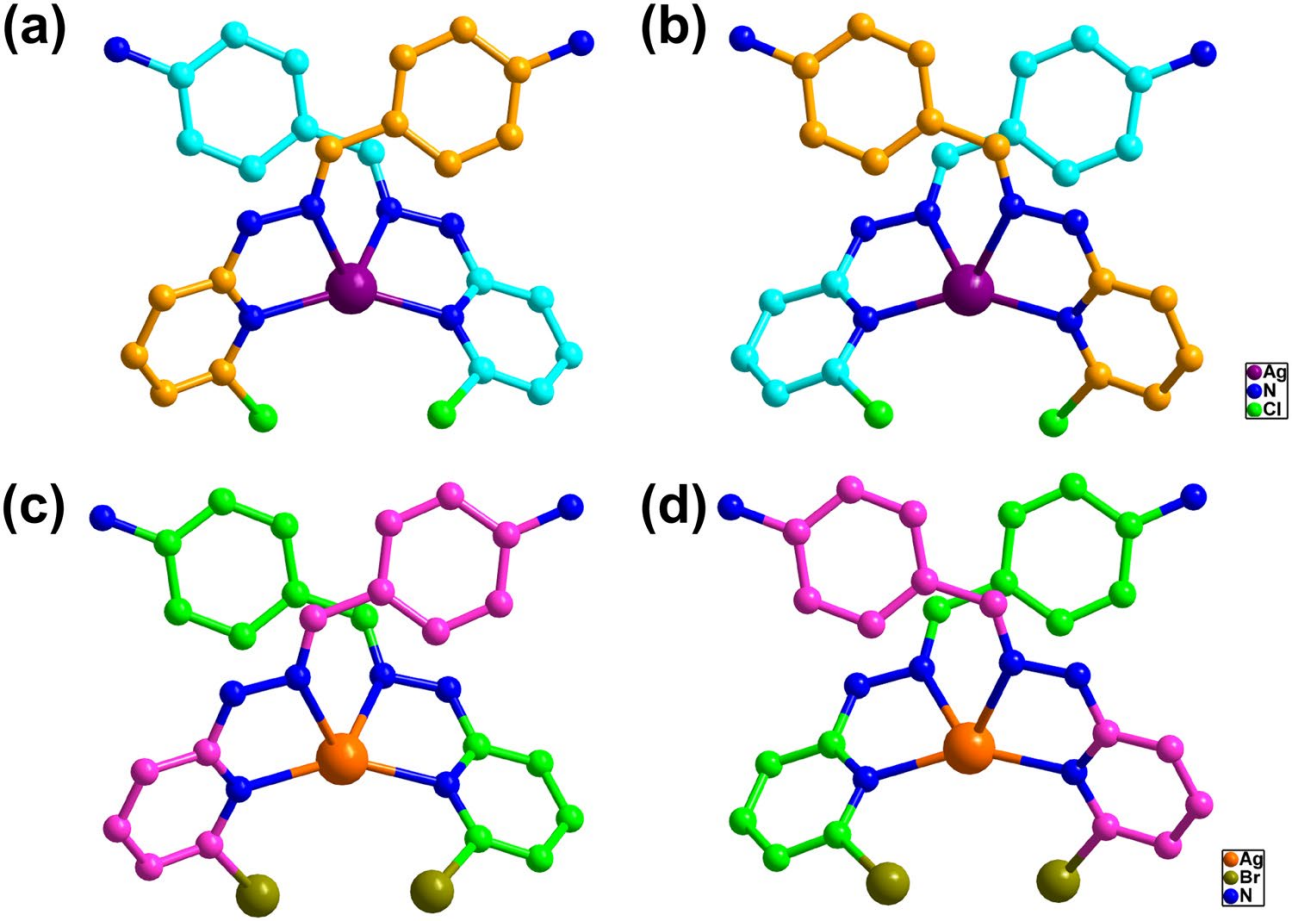
Structures of **2** and **3**. Molecular structures of **2-** ***P*** (**a**), **2-** ***M*** (**b**), **3-** ***P*** (**c**) and **3-** ***M*** (**d**) showing the chiral configuration of the silver(I) centers in all cases.

**Figure 7:**
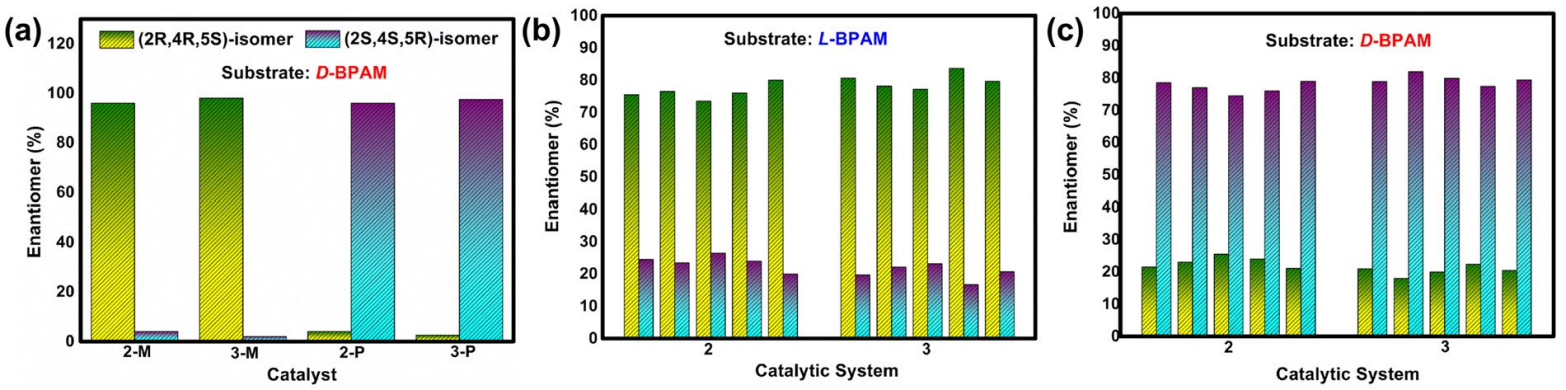
Enantioselectivity of the catalytic systems **2** and **3**. (**a**) The enantioselectivity of the asymmetric 1,3-dipolar cycloaddition with the substrate of ***D*** **-BPAM** and the catalysts of the homochiral MOF materials **2** and **3**. The enantioselectivity of the cycloaddition products yielded in the tandem systems of **2** and **3** with the presence of the substrate of ***L*** **-BPAM** (**b**) and ***D*** **-BPAM** (**c**).

The good induction effects of enantiopure **BPAM** molecules also applied to the other propeller-like ligands, **L**_**2**_ and **L**_**3**_, within which sterically hindered halogen atoms (Cl and Br) were introduced into the ortho-position of the pyridine nitrogen atom (Fig. 1). According to the same procedures used for **1**, 10 mol% ***L*** **-BPAM** was added to the interlayer of the crystallization systems, homochiral Ag-MOFs of **2-** ***M*** and **3-** ***M*** were generated a few days later. Structural analysis of **2-** ***M*** and **3-** ***M*** revealed that they are isostructural with **1**, both possessing a chiral *I*2_1_3 space group and an intrinsic chiral (10,3)-*a* network (Fig. 6 and Figures S9–S14). Randomly selected crystals from one single crystallization system of **2-** ***M*** or **3-** ***M*** showed that each crystal was homochiral with ***M***-configuration of silver atom (Flack parameters approach zero) (Figures S29 and S31, Tables S6 and S8). Similarly, crystals of **2-** ***P*** and **3-** ***P***, with the same structural framework as **2-** ***M*** and **3-** ***M*** but completely opposite chirality, were produced using ***D*** **-BPAM** as the CIAs (Figures S30 and S32, Tables S7 and S9). These results indicated that the enantiopure **BPAM** molecules could induce the formation of other similar homochiral Ag(I)-MOF materials. Under the same conditions as those [3 + 2] cycloaddition experiments used **1-** ***M***, when employing ***D*** **-BPAM** as the substrate, the loading of 3% equiv. crystals **2-** ***M*** also led to only *endo* product with an approximate 85% conversion and 92% *ee*, while a comparable conversion of 84% *endo* product with an opposite enantiomeric excess of −92% was obtained when employing the crystals of **2-** ***P***. This opposite enantioselectivity confirmed the powerful chirality direction of the enantiopure **BPAM** molecules on the MOF materials. Similar results were also observed in the catalytic systems of **3-** ***M*** and **3-** ***P***, respectively (Fig. 7a and Figures S61–S64).

The tandem homochiral crystalization and asymmetric catalysis approach also extended to these new homochiral materials (Fig. 7b,c). Under the same conditions as those used in the system of **1**, a higher average conversion of 90% and larger *ee* values (ranging from 47 to 60%) were obtained from five parallel batches involving ***L*** **-BPAM** and ligand **L**_**2**_ as well as silver salts. Among the similar tandem systems with **L**_**3**_, the reactions resulted in an average conversion of 89% and *ee* value of 59%. Conversely, when ***D*** **-BPAM** was added prior to the crystallization systems of **2** or **3** under the same reaction conditions, the comparable conversions but fully opposite *ee* distribution were observed (Tables S11–S12 and Figures S65–S68). Comparing the catalytic experiments used **2-** ***M*** (or **3-** ***M***) with the corresponding tandem experiments containing ***L*** **-BPAM** (Fig. 7a,b), clearly the addition of substrates with opposite chirality but yielded the adducts with the identical enantioselectivity, which suggested the importance of the intermolecular chiral interactions to the control of the handness and enantioselective performances of the MOF materials.

## Discussion

Generally, the synergistically controlling the chirality of the MOF materials assembled by achiral precursors and the combined processes merging homochiral self-resolution crystallization and asymmetric transformation are difficult. However, good crystallization induction effects and catalytic results were achieved in the tandem experiments. Therefore, such a presumption could be concluded: the chiral **BPAM** molecule first interacted with silver(I) ion and that this interaction subsequently induced the initial chirality of silver(I) centers. The atropisomeric chirality of the trisphenylamine moieties thus facilitated the chiral transfer between the silver centers, leading to the symmetry breaking during the competitive crystallizations. The potential coordination saturation and space steric hindrance lead the **BPAM** molecule excluded from the coordination sphere of the metal center. The departing **BPAM** substrate distributed around the metal center, is easy to be activated with the conformation affected by the chiral micro-environment around the metal ions. It briefly bonds back again with the silver(I) ion until the methyl acrylate arrives. The generated intermediate further duplicated the stored chirality of silver(I) centers to produce one enantiomer of the [3 + 2] cycloaddition product. It is worth emphasizing that the special fashion of chelating coordination requires a matching local chiral micro-environments: an appropriate angle, configuration and direction, which is advantageous for the “go and back” of the **BPAM** substrate^41,42^. It is these specific interactions and matching environments that allow the induction resolution and asymmetric catalysis processes to proceed with a chirality-control manner.

To sum up, we developed a new system that the incorporation of enantiopure **BPAM** molecules acting as both CIAs and substrates to induce the homochiral crystallization of Ag(I)-MOF materials assembled by achiral precursors, and to directionally facilitate the asymmetric [3 + 2] cycloaddition transformation without the separation of the induction-formed chiral materials, which properly presented the complex intermolecular chiral interactions and chirality transfer during the spontaneous resolution and asymmetric transformation. The catalytic results were used as evidence to assess the effects of the chiral interactions on the chirality direction of the MOF materials and the enantioselectivity control of the adducts. The crystalline structural characteristics of the materials encapsulating homochiral CIAs with clear absolute configuration precisely displayed the matched chiral interactions and ways between CIAs and materials, which represented a new approach to understand the principles of the chiral induction and homochiral self-assembly of materials. More importantly, the addition of homochiral substrates before and after crystallization yielded different enantioselective performances, which further supported the crucial influence of the intermolecular chiral interactions on the controllable direction of the chirality of homochiral MOF materials. Such a new strategy of merging a series of complicated self-inductions, bulk homochiral crystallization and Ag(I)-based enantioselective catalysis achieved the most economical chirality amplification without introducing any additional chiral source, opens new avenues for enantiopure chemical synthesis and provides a promising path for the directional design and development of homochiral materials.

## Methods

### Materials and general procedures

Unless otherwise specified, all chemicals were of reagent grade quality, were obtained from commercial sources and used without further purification. Tris(4-formylphenyl)amine, (*S*,*E*)-methyl-2-(benzylideneamino) propanoate (***L*** **-BPAM**) and (*R*,*E*)-methyl-2-(benzylideneamino)propanoate (***D*** **-BPAM**) were synthesized according to published procedures^35,43^. **L**_**1**_~**L**_**3**_ were synthesized through Schiff-base reactions (Figure S33–S38). ^1^H NMR spectra were recorded at 400 MHz. ^13^C NMR, ^1^H-^1^H COSY, ^1^H-^1^H NOESY and DEPT 135 (distortionless enhancement by polarization transfer at pulse angle *θ* = 135°) spectra were recorded on a Varian INOVA 500 M spectrometer. Tetramethylsilane (TMS) served as an internal reference (*δ* = 0) for^1^H NMR, and CDCl_3_ served as an internal standard (*δ* = 77.0) for ^13^C NMR. The elemental analyses of C, H and N were performed on a Vario EL III elemental analyzer. FT-IR spectra were recorded as KBr pellets on JASCO FT/IR-430. The CD spectra were measured on a JASCO J-810; the crystalline samples were ground into a powder and compacted into KBr pellets for analysis. HPLC analysis was performed on an Agilent 1100 using ChIRALPAK AS-H (0.46 cml.D. × 25 cmL) or AD-H (0.46 cml.D. × 25 cmL) columns purchased from Daicel Chemical Industries, Ltd. Products were purified by flash column chromatography on 200–300 mesh silica gel and Kromasil Lc-80 using a SiO_2_ chromatographic column. Optical rotations were obtained on a WZZ-2S polarimeter and reported as follows: [α]_D_^T^ (*c* in g per 100 mL, solvent). High resolution mass spectra were obtained on a GCT-CA156 Micromass GC/TOF mass spectrometer. ESI-MS were performed on a Agilent 6130 MSD mass spectrometer.

### Preparation of 1, 1-*M* and 1-*P*

Two drops of acetic acid were added to the suspension of **L**_**1**_ (15.2 mg, 0.025 mmol) in 4 mL CH_3_OH/CHCl_3_ (*v*:*v* = 1:3); then, 8 mL of CH_3_CN/CHCl_3_ (*v*:v = 5:3) was layered carefully, over which AgClO_4_ (7.8 mg, 0.0375 mmol) dissolved in CH_3_CN (6 mL) was layered. The container was covered and stored in the dark for the slow diffusion of reactants at room temperature, which afforded yellow crystals within one week. Yield: 46% (based on Ag). Anal. calcd for C_77_H_73_N_22_Ag_3_Cl_6_O_15_: C 44.40, H 3.53, N 14.79; found: C 44.62, H 4.10, N 14.34. **1-** ***M*** and **1-** ***P*** were synthesized according to the same procedure except by adding (*S*,*E*)-methyl-2-(benzylideneamino)propanoate (***L*** **-BPAM**) (0.48 mg, 0.0025 mmol) and (*R*,*E*)-methyl-2-(benzylideneamino)propanoate (***D*** **-BPAM**) (0.48 mg, 0.0025 mmol) into the interlayer CH_3_CN/CHCl_3_ solution, respectively.

### Preparation of 2, 2-*M* and 2-*P*.

**L**_**2**_(17.7 mg, 0.025 mmol) was dissolved in 4 mL CH_3_OH/CHCl_3_ (*v*:*v* = 1:3); then, 8 mL CH_3_CN/CHCl_3_ (*v*:*v* = 5:3) was layered carefully, over which AgClO_4_ (7.8 mg, 0.0375 mmol) dissolved in CH_3_CN (6 mL) was layered. The container was covered and stored in the dark for the slow diffusion of reactants at room temperature, which afforded yellow crystals within one week. Yield: 38% (based on Ag). Anal. calcd for C_78_H_62_N_22_Ag_3_Cl_15_O_12_: C 39.78, H 2.65, N 13.09; found: C 39.82, H 2.60, N 13.03. **2-** ***M*** and **2-** ***P*** were synthesized according to the same procedure except with ***L*** **-BPAM** (0.48 mg, 0.0025 mmol) and ***D*** **-BPAM** (0.48 mg, 0.0025 mmol) added to the interlayer CH_3_CN/CHCl_3_ solution, respectively.

### Preparation of 3, 3-*M* and 3-*P*

Two drops of acetic acid were added to the suspension of **L**_**3**_ (20.9 mg, 0.025 mmol) in 3 mL CHCl_3_; then, 10 mL CH_3_CN/CHCl_3_ (*v*:*v = *1:1) was layered carefully, over which AgClO_4_ (7.8 mg, 0.0375 mmol) dissolved in CH_3_CN (3 mL) was layered. The container was covered and stored in the dark at room temperature for the slow diffusion of reactants, which afforded yellow crystals within one week. Yield: 32% (based on Ag). Anal. calcd for C_78_H_62_N_22_Ag_3_Br_6_Cl_9_O_12_: C 35.74, H 2.38, N 11.75; found: C 35.62, H 2.20, N 11.64. **3-** ***M*** and **3-** ***P*** were synthesized according to the same procedure except with ***L*** **-BPAM** (0.48 mg, 0.0025 mmol) and ***D*** **-BPAM** (0.48 mg, 0.0025 mmol) added to the interlayer CH_3_CN/CHCl_3_ solution, respectively.

### Typical procedure for the 1,3-dipolar cycloaddition between chiral BPAM and methyl acrylate

A mixture of ***L*** **-BPAM** (0.2 mmol) [or ***D*** **-BPAM** (0.2 mmol)] and methyl acrylate (0.24 mmol) was added to a suspension of the Ag-catalyst (0.006 mmol) in chloroform (0.5 mL). To the resulting suspension, Et_3_N (0.02 mmol, 2.77 μL) was added, and the mixture was stirred at room temperature for 24 h (in the absence of the light). The precipitate was filtered, washed with chloroform three times and then collected by centrifugation for catalyzing a new batch. The organic filtrate was directly evaporated, and the residue was purified by preparative thin-layer chromatography (PTLC) with hexane-ethyl acetate (3:1) as the eluent to give the pure product as a colorless oil.

### Typical tandem procedure

Onto a solution of **L**_**1**_ (9.1 mg, 0.015 mmol) in 2 mL of CH_3_OH/CHCl_3_ (*v*:*v* = 1:3), a solution of ***L*** **-BPAM** (95.5 mg, 0.5 mmol) or ***D*** **-BPAM** (95.5 mg, 0.5 mmol) in 3 mL of CH_3_CN/CHCl_3_ (*v*:*v* = 1:1) was carefully layered, over which AgClO_4_ (4.4 mg, 0.023 mmol) dissolved in CH_3_CN (1 mL) was layered. The container was covered and stored in the dark for the slow diffusion of reactants at room temperature. After complete crystallization; Et_3_N (0.05 mmol, 6.93 μL) and methyl acrylate (0.6 mmol) were added to the reaction system, which was then covered and stirred at room temperature for 24 h in the absence of light; the precipitate was filtered, and the complex was recovered. The organic filtrate was directly evaporated, and the residue was purified by preparative thin-layer chromatography (PTLC) with hexane-ethyl acetate (3:1) as the eluent to give the pure product as a colorless oil.

### X-ray crystallography

Single crystals with suitable dimensions were selected under an optical microscope and mounted onto a glass fiber for data collection. Intensity data for all crystals were collected at 220 K on a Bruker SMART APEX diffracto- meter equipped with a CCD area detector and a Mo-K*α* (*λ* = 0.71073 Å) radiation source. The data integration and reduction were processed using the SAINT software^44^. An empirical absorption correction was applied to the collected reflections with SADABS^45^. The structures were solved by direct methods using SHELXTL and were refined on *F* ^2^ by the full-matrix least-squares method using the program SHELXL-97^46,47^.

### Data availability

The X-ray crystallographic coordinates for structures reported in this Article have been deposited at the Cambridge Crystallographic Data Centre (CCDC) under deposition numbers CCDC 1417887–1417898, 1417905–1417911, 1448704–1448709, 1448740–1448745, 1449160–1449171 and 1457367–1457369 (Tables S1–S9). These data can be obtained free of charge from The Cambridge Crystallographic Data Centre via www.ccdc.cam.ac.uk/ data_request /cif. All other data, if not included in the Article or the Supplementary Information, are available from the authors on request.

## Electronic supplementary material

supporting information
