# Supplementary material for: Coordinative Alignment of Chiral Molecules to Control over the Chirality Transfer in Spontaneous Resolution and Asymmetric Catalysis
Source: Sci Rep. 2017 Nov 13;7:15418. doi: 10.1038/s41598-017-15780-0 (PMC5684417; doi:10.1038/s41598-017-15780-0)
Supplement: Supplementary file 1 — supporting information [file 41598_2017_15780_MOESM1_ESM.pdf]

Electronic Supplementary Information for:

Coordinative Alignment of Chiral Molecules to Control over the Chirality Transfer in Spontaneous Resolution and Asymmetric Catalysis

Zhengqiang Xia,[a,b] Xu Jing,[a,b,c] Cheng He,[a] Xiaoge Wang[a] & Chunying Duan[a,b,c]*

[a] State Key Laboratory of Fine Chemicals, Dalian University of Technology, Dalian 116024

[b] College of Zhang Dayu, Dalian University of Technology, Dalian 116024

[c] Collaborative Innovation Center of Chemical Science and Engineering, Tianjin 300071, China

Correspondence and requests for materials should be addressed to C.D. (E-mail: cyduan@dlut.edu.cn)

Supplementary Methods

Synthesis of tris(4-formylphenyl)amine[1]

Phosphorus oxychloride (100 mL, 1.09 mol) was added dropwise at 0 °C under N_2 to DMF (75.0 mL, 0.97 mol) and the reaction mixture was stirred for 1 h. Triphenylamine (10.0 g, 40.8 mmol) was added in batches, and the resulting mixture was stirred at 95 °C for 8 h. After cooling to r.t., the mixture was poured into ice-water (500 mL), and basified with 1 M NaOH. After extraction with CH_2Cl_2 (400 mL), the organic layer was washed with water (3 × 150 mL), dried with Na_2SO_4. After filtration and removal of the solvent in vacuo, the residue was added on an ice-cooled mixture of $POCl_3$ (80.0 mL, 0.87 mol) and DMF (60.0 mL, 0.78 mol). The resulting mixture was stirred at 95 °C for 4 h, and after cooling to r.t., poured into ice-water (500 mL), and basified with 1 M NaOH. After extraction with CH_2Cl_2 (400 mL), the organic layer was washed with water (3 × 150 mL) and dried with Na_2SO_4. After filtration and removal of the solvent under reduced pressure, the crude product was purified by column chromatography with CH_2Cl_2 as an eluent to give a yellow solid (7.85 g, 58.4%). ^{1}H NMR (400 MHz, $CDCl_3$), δ (ppm): 9.95 (s, 3H), 7.85 (d, J = 8.6 Hz, 6H), 7.26 (d, J = 8.6 Hz, 6H).

Synthesis of Tris(4-(2-pyridin-2-ylhydrazono)phenyl)amine (L_1)

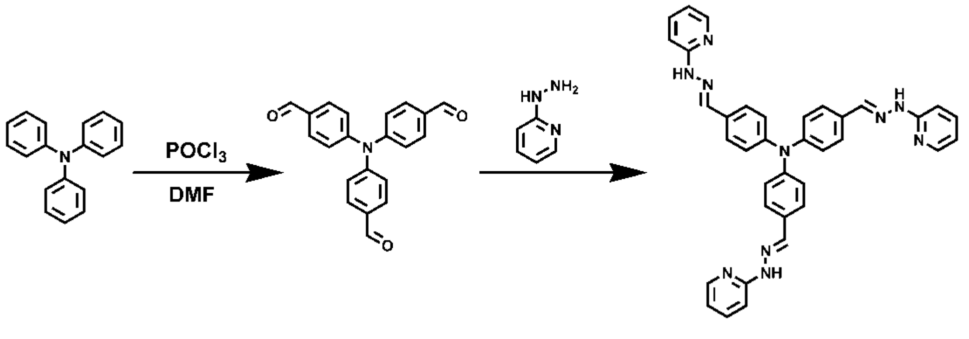

L_1

Five drops of acetic acid were added to a mixture of tris(4-formylphenyl)amine (0.33 g, 1 mmol) and 2-hydrazinopyridine (0.34 g, 3.1 mmol) in a dry CH_3OH solution (25 mL). The mixture was refluxed over 4 h and a yellow precipitate (0.55 g) was isolated by filtration, washed with dry CH_3OH and dried under vacuum. Yield: 91%. Anal. calcd. for $C_{36}H_{30}N_{10}$: C, 71.74; H, 5.02; N, 23.24. Found: C, 71.78; H, 5.26; N, 22.91. ^{1}H NMR (400 MHz, DMSO-d_6), δ (ppm): 10.82 (s, 3H), 8.10 (d, J = 3.9 Hz, 3H), 7.99 (s, 3H), 7.62 (t, J = 8.2 Hz, 9H), 7.20 (d, J = 8.5 Hz, 3H), 7.10 (dd, J = 16.9, 8.6 Hz, 6H), 6.78–6.70 (m, 3H). ESI-MS: *m/z* 603.1 (calcd. *m/z* 603.27 for $[L+H]^+$).

Synthesis of Tris(4-(6-chloro-2-pyridin-2-ylhydrazono)phenyl)amine (L_2)

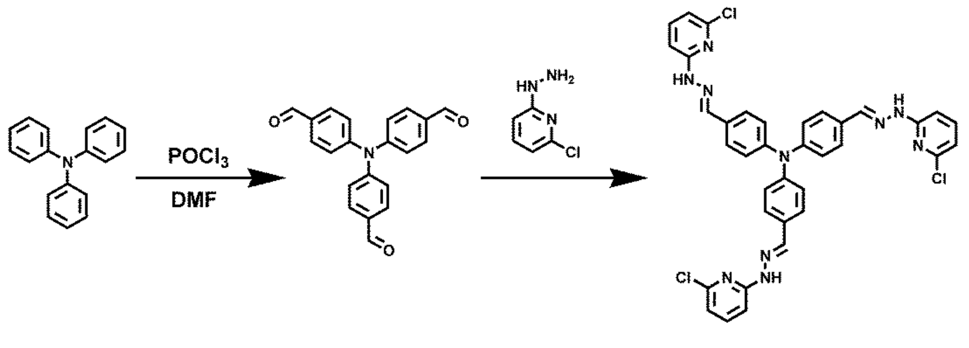

L_2

Five drops of acetic acid were added to a mixture of tris(4-formylphenyl)amine (0.33 g, 1 mmol) and 2-chloro-6-hydrazinopyridine (0.44 g, 3.1 mmol) in a dry CH_3OH solution (25 mL). The mixture was refluxed for 6 h, and a yellow precipitate (0.62 g) was isolated by filtration, washed with dry CH_3OH and dried under vacuum. Yield: 89%. Anal. calcd. for $C_{36}H_{27}N_{10}Cl_3$: C, 61.24; H, 3.85; N, 19.84. Found: C, 61.66; H, 3.46; N, 19.43. ^{1}H NMR (400 MHz, DMSO-d_6), δ (ppm): 11.21 (s, 3H), 7.99 (s, 3H), 7.76–7.52 (m, 9H), 7.15 (d, J = 8.3 Hz, 3H), 7.06 (t, J = 11.4 Hz, 6H), 6.79 (d, J = 7.5 Hz, 3H). ESI-MS: *m/z* 705.1 (calcd. *m/z* 705.15 for $[L-H]^-$).

Synthesis of Tris(4-(6-bromo-2-pyridin-2-ylhydrazono)phenyl)amine (L_3)

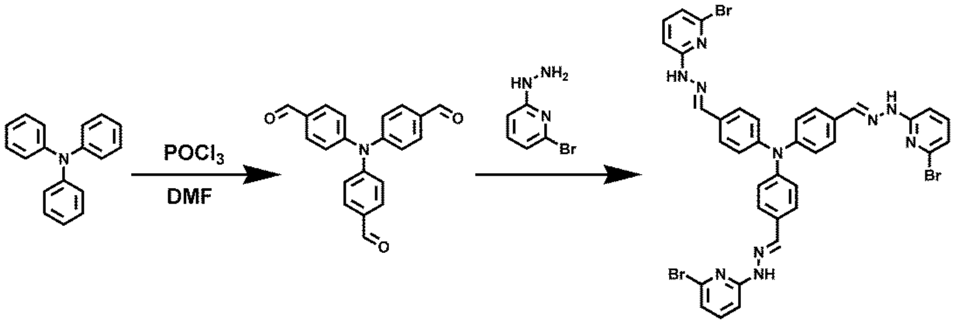

L_3

Five drops of acetic acid were added to a mixture of tris(4-formylphenyl)amine (0.33 g, 1 mmol) and 2-bromo-6-hydrazinopyridine (0.58 g, 3.1 mmol) in a dry CH_3OH solution (25 mL). The mixture was refluxed for 6 h, and a yellow precipitate (0.78 g) was isolated by filtration, washed with dry CH_3OH and dried under vacuum. Yield: 92%. Anal. calcd. for $C_{36}H_{27}N_{10}Br_3$: C, 51.51; H, 3.24; N, 16.69. Found: C, 51.88; H, 3.44; N, 16.63. ^{1}H NMR (400 MHz, DMSO-d_6), δ (ppm): 11.23 (s, 3H), 7.98 (s, 3H), 7.63 (d, J = 8.6 Hz, 6H), 7.55 (t, J = 7.9 Hz, 3H), 7.17 (d, J = 8.2 Hz, 3H), 7.07 (d, J = 8.6 Hz, 6H), 6.92 (d, J = 7.4 Hz, 3H). ESI-MS: *m/z* 837.0 (calcd. *m/z* 836.99 for $[L-H]^-$).

Synthesis of 1. Two drops of acetic acid were added to the suspension of $\mathbf{L_1}$ (15.2 mg, 0.025 mmol) in 4 mL $CH_3OH/CHCl_3$ (v:v = 1:3); then, 8 mL $CH_3CN/CHCl_3$ (v:v = 5:3) were layered carefully, over which $AgClO_4$ (7.8 mg, 0.0375 mmol) dissolved in CH_3CN (6 mL) was layered. The container was covered and stored in the dark for slow diffusion of reactants at room temperature, which afforded yellow crystals within one week time. Yield: 46% (based on Ag). Elemental analysis (%) Calc. for $C_{77}H_{73}N_{22}Ag_3Cl_6O_{15}$: H, 3.53; C, 44.40.; N, 14.79. Found: H, 4.10; C, 44.62; N, 14.34. IR cm^{-1} (KBr): 3476 (br), 3267 (w), 3032 (w), 1615 (s), 1598 (s), 1577 (m), 1538 (m), 1503 (s), 1483 (s), 1444 (m), 1423 (s), 1320 (s), 1286 (s), 1248 (s), 1177 (m), 1095 (s), 1004 (m), 927 (w), 827 (w), 768 (m), 738 (w), 667 (w), 622 (m), 555(w), 529 (w).

Synthesis of 1-*M*. Two drops of acetic acid were added to the suspension of $\mathbf{L_1}$ (15.2 mg, 0.025 mmol) in 4 mL $CH_3OH/CHCl_3$ (v:v = 1:3); then, the solution of (S,E)-methyl 2-(benzylideneamino)propanoate (***L*-BPAM**)[2] (0.48 mg, 0.0025 mmol) in 8 mL $CH_3CN/CHCl_3$ (v:v = 5:3) was layered carefully, over which $AgClO_4$ (7.8 mg, 0.0375 mmol) dissolved in CH_3CN (6 mL) was layered. The container was covered and stored in the dark for slow diffusion of

reactants at room temperature, which afforded yellow crystals within one week time. Yield: 42% (based on Ag). Elemental analysis (%) Calc. for $C_{77}H_{73}N_{22}Ag_3Cl_6O_{15}$: H, 3.53; C, 44.40.; N, 14.79. Found: H, 3.72; C, 44.73; N, 14.74. IR cm^{-1} (KBr): 3443 (br), 3280 (w), 3032 (w), 1615 (s), 1599 (s), 1578 (m), 1538 (m), 1503 (s), 1483 (s), 1444 (w), 1423 (s), 1320 (s), 1286 (s), 1248 (s), 1177 (w), 1095 (s), 1004 (w), 926 (w), 827 (w), 768 (m), 738 (w), 667 (w), 622 (m), 555(w), 530 (w).

Synthesis of 1-*P*. An identical procedure with **1-*M*** was followed to prepare **1-*P*** except ***L*-BPAM** was replaced by (*R*,*E*)-methyl-2-(benzylideneamino)propanoate (***D*-BPAM**).[2] Yellow crystals of **1-*P*** were collected in 44% yield (based on Ag). Elemental analysis (%) Calc. for $C_{77}H_{73}N_{22}Ag_3Cl_6O_{15}$: H, 3.53; C, 44.40.; N, 14.79. Found: H, 4.08; C, 45.10; N, 14.66. IR cm^{-1} (KBr): 3444 (br), 3276 (w), 1615 (s), 1600 (s), 1577 (m), 1538 (m), 1502 (s), 1483 (s), 1444 (m), 1423 (s), 1320 (s), 1286 (s), 1248 (s), 1177 (m), 1095 (s), 1004 (m), 926 (w), 827 (w), 768 (m), 738 (w), 667 (w), 622 (m), 556(w), 530 (w).

Syntheses of 2, 2-*M* and 2-*P*. $\mathbf{L_2}$ (17.7 mg, 0.025 mmol) was dissolved in 4 mL $CH_3OH/CHCl_3$ (*v*:*v* = 1:3); then, 8 mL $CH_3CN/CHCl_3$ (*v*:*v* = 5:3) were layered carefully, over which $AgClO_4$ (7.8 mg, 0.0375 mmol) dissolved in CH_3CN (6 mL) was layered. The container was covered and stored in the dark for slow diffusion of reactants at room temperature, which afforded yellow crystals within one week time. Yield: 38% (based on Ag). Elemental analysis (%) Calc. for $C_{78}H_{62}N_{22}Ag_3Cl_{15}O_{12}$: H, 2.65; C, 39.78.; N, 13.09. Found: H, 2.60; C, 39.82; N, 13.03. IR cm^{-1} (KBr): 3443 (br, m), 3262 (m), 2958 (s), 2868(m), 1601 (s), 1531 (m), 1503 (s), 1457 (s), 1401 (m), 1321 (s), 1280 (s), 1254 (s), 1174 (s), 1103 (s), 1073 (s), 996 (m), 959 (w), 925 (m), 885 (m), 826 (m), 762 (m), 731 (w), 663 (w), 617 (m), 553 (w), 522 (w). **2-*M*** and **2-*P*** were synthesized according to the same procedure except by adding ***L*-BPAM** (0.48 mg, 0.0025 mmol) and ***D*-BPAM** (0.48 mg, 0.0025 mmol) in the interlayer $CH_3CN/CHCl_3$ solution, respectively.

Syntheses of 3, 3-*M* and 3-*P*. Two drops of acetic acid were added to the suspension of $\mathbf{L_3}$ (20.9 mg, 0.025 mmol) in 3 mL $CHCl_3$; then, 10 mL $CH_3CN/CHCl_3$ (*v*:*v* = 1:1) were layered carefully, over which $AgClO_4$ (7.8 mg, 0.0375 mmol) dissolved in CH_3CN (3 mL) was layered. The container was covered and stored in the dark for slow diffusion of reactants at room temperature, which afforded yellow crystals within one week time. Yield: 32% (based on Ag). Elemental analysis (%) Calc. for $C_{78}H_{62}N_{22}Ag_3Br_6Cl_9O_{12}$: H, 2.38; C, 35.74.; N, 11.75. Found: H, 2.20; C,

35.62; N, 11.64. IR cm^{-1} (KBr): 3443 (br, m), 3262 (m), 2924 (w), 1612 (s), 1598 (s), 1580 (m), 1527 (m), 1500 (s), 1481 (s), 1457 (s), 1429 (m), 1398 (s), 1318 (s), 1279 (m), 1254 (s), 1171 (s), 1097 (s), 989 (m), 956 (m), 928 (m), 860 (w), 823 (m, 767 (m), 735 (w), 664 (w), 619 (m), 550 (w), 522 (w). **3-*M*** and **3-*P*** were synthesized according to the same procedure except by adding ***L*-BPAM** (0.48 mg, 0.0025 mmol) and ***D*-BPAM** (0.48 mg, 0.0025 mmol) in the interlayer $CH_3CN/CHCl_3$ solution, respectively.

Synthesis of *L*-BPAM@1-*M*. Crystals of the compound were obtained soaking the crystals of **1-*M*** (0.01 mmol) in a $CHCl_3$ (1 mL) solution containing ***L*-BPAM** (1 mmol) for 24 hours.

Synthesis of *D*-BPAM@1-*P*. Crystals of the compound were obtained soaking the crystals of **1-*P*** (0.01 mmol) in a $CHCl_3$ (1 mL) solution containing ***D*-BPAM** (1 mmol) for 24 hours.

Synthesis of *D*-BPAM@1-*M*. Crystals of the compound were obtained soaking the crystals of **1-*M*** (0.01 mmol) in a $CHCl_3$ (1 mL) solution containing ***D*-BPAM** (1 mmol) for 24 hours.

Synthesis of *L*-BPAM@1-*P*.Crystals of the compound were obtained soaking the crystals of **1-*P*** (0.01 mmol) in a $CHCl_3$ (1 mL) solution containing ***L*-BPAM** (1 mmol) for 24 hours.

Synthesis of (*S*,*E*)-methyl 2-(benzylideneamino)propanoate (*L*-BPAM)[2]

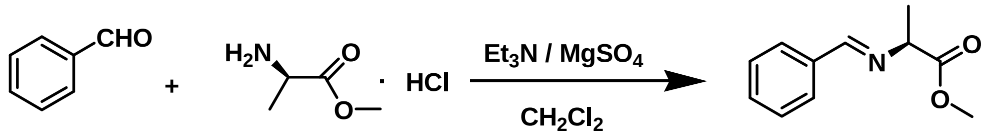

A suspension of *L*-alanine methyl ester hydrochloride (3.53 g, 25.3 mmol) and $MgSO_4$ (3.2 g, 26.7 mmol) in CH_2Cl_2 (50 mL) was stirred at 0 °C, TEA (3.5 mL, 25.3 mmol) was added dropwise. And then benzaldehyde (2.1 mL, 21.1 mmol) was added dropwise over 10 min. The reaction was allowed to warm to ambient temperature and stirred for 24 h. Et_2O (50 mL) was then added and the solution was filtered. The filtrate was washed with water (× 2) and brine (× 2), dried ($MgSO_4$) and concentrated *in vacuo* to give the target compound as a light-yellow oil (3.59 g, 89 %).^{1}H NMR (400 MHz, $CDCl_3$), δ (ppm): 8.31 (s, 1H), 7.78 (dt, J = 3.7, 2.2 Hz, 2H), 7.45–7.38 (m, 3H), 4.16 (q, J = 6.8 Hz, 1H), 3.74 (s, 3H), 1.53 (d, J = 6.8 Hz, 3H).

Synthesis of (*R*,*E*)-methyl 2-(benzylideneamino)propanoate (*D*-BPAM)[2]

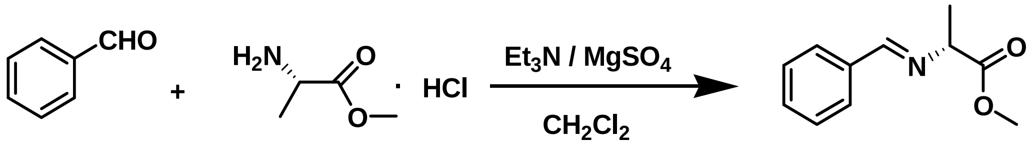

The same procedure as that for ***L*-BPAM** was used, except that *D*-alanine methyl ester hydrochloride was used instead of *L*-alanine methyl ester hydrochloride. Light-yellow oil (3.47 g, 86 %) was obtained in the end. ^{1}H NMR (400 MHz, $CDCl_3$), δ (ppm): 8.31 (s, 1H), 7.85–7.72 (m, 2H), 7.45–7.37 (m, 3H), 4.15 (t, J = 6.8 Hz, 1H), 3.74 (s, 3H), 1.53 (d, J = 6.8 Hz, 3H).

Synthesis of N-(Phenylmethylene)-glycine ethyl ester (PGE)

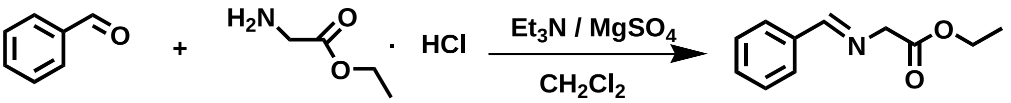

To a suspension of ethyl glycinate hydrochloride (2.065 g, 14.8 mmol) and $MgSO_4$ (2.17 g, 18.0 mmol) in CH_2Cl_2 (36 mL), TEA (2.10 mL, 14.8 mmol) was added dropwise. The mixture was stirred at 0 °C for one hour, and then benzaldehyde (1.06 mL, 10.0 mmol) was added dropwise over 10 min. The reaction was allowed to warm to ambient temperature and stirred for 12 h. The solution was filtered, and the filtrate was washed with water (10 mL × 2), the aqueous layer was extracted with CH_2Cl_2 (10 mL × 2). The combined organic layers were washed with brine (× 2), dried ($MgSO_4$) and concentrated *in vacuo* to give the target compound as a light-yellow oil (1.61 g, 84 %). ^{1}H NMR (400 MHz, $CDCl_3$), δ (ppm): 8.28 (s, 1H), 7.88–7.68 (m, 2H), 7.49–7.26 (m, 3H), 4.39 (s, 2H), 4.23 (q, J = 7.1 Hz, 2H), 1.30 (t, J = 7.1 Hz, 3H).

General procedure for the 1,3-dipolar cycloaddition reaction between chiral methyl 2-(benzylideneamino)propanoate and methyl acrylate[3]

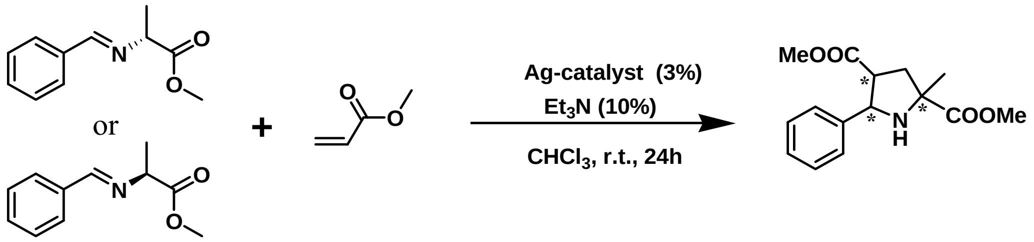

A mixture of (*S*,*E*)-methyl 2-(benzylideneamino)propanoate (0.2 mmol) [or (*R*,*E*)-methyl 2-(benzylideneamino)propanoate (0.2 mmol)] and methyl acrylate (0.24 mmol) was added to a suspension of the Ag-catalyst (0.006 mmol) in chloroform (0.5 mL). To the resulting suspension Et_3N (0.02 mmol, 2.77 μL) was added and the mixture was stirred at room temperature for 24 h (in the absence of the light). The precipitate was filtered, washed with chloroform three times and then collected by centrifugation for catalyzing a new batch. The organic filtrate was directly evaporated and the residue was purified by preparative thin-layer chromatography (PTLC) with hexane-ethyl acetate (3:1) as the eluent to give the pure product as colorless oil. ^{1}H NMR (500 MHz, $CDCl_3$), δ (ppm): 7.32–7.20 (m, 5H), 4.66 (d, J = 7.5 Hz, 1H), 3.82 (s, 3H), 3.35 (td, J = 7.5, 5.3 Hz, 1H), 3.20 (s, 3H), 3.15 (s, 1H), 2.72 (dd, J = 13.6, 5.2 Hz, 1H), 2.05 (dd, J = 13.6, 7.5 Hz, 1H), 1.50 (s, 3H). ^{13}C NMR (126 MHz, $CDCl_3$) δ 176.68, 173.15, 139.19, 128.30, 127.69, 126.81, 65.89, 65.09, 52.63, 51.27, 50.62, 40.44, 27.68. HPLC Chiral pak AS-H, *i*PrOH/hexane = 10:90, flow rate = 0.8 mL/min, 210 nm, t_R = 13.9 min, (2*S*,4*S*,5*R*)-isomer; t_R = 8.7 min, (2*R*,4*R*,5*S*)-isomer. ESI-HRMS (m/z) calcd. for $C_{15}H_{20}NO_4$ $(M+H)^+$: 278.1392; found: 278.1393.

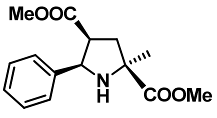

(2S,4S,5R)-isomer

(2*S*,4*S*,5*R*)-Dimethyl-2-methyl-5-phenylpyrrolidine-2,4-dicarboxylate: $[\alpha]_D^{20}$ = 18.2 (*c* = 0.73, $CHCl_3$, 91% ee). Absolute configuration determined in comparison to similar compound in Literature $[\alpha]_D$ = 33.1 (c = 1.11, CH_2Cl_2).[4] ^{1}H NMR and ^{13}C NMR data were consistent with previously reported value.[5] ESI-HRMS (m/z) calcd. for $C_{15}H_{20}NO_4$ $(M+H)^+$: 278.1392; found: 278.1394.

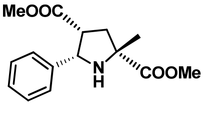

(2R,4R,5S)-isomer

(2*R*,4*R*,5*S*)-Dimethyl-2-methyl-5-phenylpyrrolidine-2,4-dicarboxylate: $[\alpha]_D^{20}$ = -19.9 (*c* = 0.78, $CHCl_3$, 92% ee). Absolute configuration determined in comparison to similar compound in Literature $[\alpha]_D$ = 33.1 (c = 1.11, CH_2Cl_2).[4] ^{1}H NMR, ^{13}C NMR and ESI-HRMS data please see above for details.

Typical one-pot tandem procedure merging the homochiral Ag(I)-based MOFs formation and the abovementioned 1,3-dipolar cycloaddition.

Onto a solution of **L_1** (9.1 mg, 0.015 mmol) in 2 mL of $CH_3OH/CHCl_3$ (*v*:*v* = 1:3), a solution of ***L*-BPAM** (95.5 mg, 0.5 mmol) or ***D*-BPAM** (95.5 mg, 0.5 mmol) in 3 mL of $CH_3CN/CHCl_3$ (*v*:*v* = 1:1) was carefully layered, over which $AgClO_4$ (4.4 mg, 0.023 mmol) dissolved in acetonitrile (1 mL) was layered. The container was covered and stored in the dark for slow diffusion of reactants at room temperature. After complete crystallization, Et_3N (0.05 mmol, 6.93 μL) and methyl acrylate (0.6 mmol) were added to the reaction system, which was then covered and stirred at room temperature for 24 h in the absence of light; the precipitate was filtered, and the complex was recovered. The organic filtrate was directly evaporated, and the residue was purified by preparative thin-layer chromatography (PTLC) with hexane-ethyl acetate (3:1) as the eluent to give the pure product as a colorless oil.

For the 'ont-pot' tandem asymmetric catalytic systems of **2** and **3**: a similar procedure as that of **1**, except **L_1** (9.1 mg, 0.015 mmol) was replaced by **L_2** (10.6 mg, 0.015 mmol) and **L_3** (12.5 mg, 0.015 mmol), respectively.

General procedure for the catalytic reaction between *N*-(phenylmethylene)-glycine ethyl ester (PGE) and methyl acrylate[6]

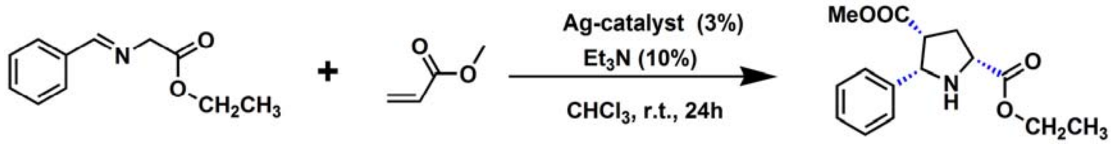

A mixture of *N*-(phenylmethylene)-glycine ethyl ester (0.2 mmol) and methyl acrylate (0.24 mmol) was added to a suspension of the Ag-catalyst (0.006 mmol) in chloroform (0.5 mL). To the resulting suspension Et_3N (0.02 mmol, 2.77 μL) was added and the mixture was stirred at room temperature for 24 h (in the absence of the light). The precipitate was filtered, washed with chloroform three times and then collected by centrifugation for catalyzing a new batch. The organic filtrate was directly evaporated and the residue was purified by preparative thin-layer chromatography (PTLC) with hexane-ethyl acetate (3:1) as the eluent to give the pure product as colorless oil. ^{1}H NMR (500 MHz, $CDCl_3$), δ (ppm): 7.31 (m, J = 7.0, 6.3 Hz, 5H), 4.55 (d, J = 7.8 Hz, 1H), 4.33–4.25 (m, 2H), 3.98 (s, 1H), 3.33 (d, J = 7.0 Hz, 1H), 3.22 (s, 3H), 2.53 (s, 1H), 2.45–2.38 (m, 2H), 1.33 (t, J = 7.1 Hz, 3H). ^{13}C NMR (126 MHz, $CDCl_3$) δ173.45, 173.18, 139.24, 128.43, 127.86, 126.99, 66.09, 61.48, 60.27, 51.44, 49.99, 33.58, 14.45. HPLC Chiral pak AS-H, *i*PrOH/hexane = 15:85, flow rate = 0.8 mL/min, 210 nm, t_R = 10.5 min; t_R = 21.8 min.

X-ray Crystallography

Summary of crystallographic data and details of data collection for **1-*M***, **1-*P***, **2-*M***, **2-*P*** and **3-*M*** are given in Table S1, and **3-*P***, ***L*-BPAM@1-*M***, ***D*-BPAM@1-*P***, ***D*-BPAM@1-*M*** and ***L*-BPAM@1-*P*** are listed in Table S2.

Single crystals with suitable dimensions were selected under an optical microscope and mounted onto a glass fiber for data collection. Intensity data for all crystals were collected at 220 K on a Bruker SMART APEX diffractometer equipped with a CCD area detector and a Mo-Kα (λ = 0.71073 Å) radiation source. The data integration and reduction were processed using the SAINT software.[7] An empirical absorption correction was applied to the collected reflections with SADABS.[8] The structures were solved by direct methods using SHELXTL and were refined on F^2 by the full-matrix least-squares method using the program SHELXL-97.[9,10] The X-ray crystallographic coordinates for structures reported in this article have been deposited at the Cambridge Crystallographic Data Centre (CCDC) under deposition numbers CCDC 1417887—1417898, 1417905—1417911, 1448704—1448709, 1448740—1448745, 1449160—1449171 and 1457367—1457369. These data can be obtained free of charge from The Cambridge Crystallographic Data Centre *via* www.ccdc.cam.ac.uk/data_request/cif.

Refinements for 1-*M*, **1-*P*, 2-*M*, 2-*P*, 3-*M*** and **3-*P***: All non-hydrogen atoms were refined anisotropically till convergence is reached. Hydrogen atoms attached to the organic ligands, chloroform and acetonitrile molecules were located geometrically and refined in a riding model. The water hydrogen atoms were located from the difference Fourier maps, with distance restraints of O—H = 0.85±0.02 Å, and then refined with isotropic thermal parameters 1.5 times those of O atoms.

Refinements for *L*-BPAM@1-*M*, ***D*-BPAM@1-*P***, ***D*-BPAM@1-*M*** and ***L*-BPAM@1-*P***: All of the non-hydrogen atoms in the MOFs backbone were refined with anisotropically. Hydrogen atoms of organic ligands and the **BPAM** molecule were located geometrically and refined in a riding model, whereas those of solvent molecules were not treated during the structural refinements. The atoms in the **BPAM** molecule were refined disordered with the site occupancy factor (*s.o.f.*) of these atoms being fixed at 1/6. To help the stability of the refinement for the encapsulated **BPAM** molecule, bond distances between several atoms were fixed, the geometrical constraints of idealized regular polygons were used for its benzene rings, and thermal parameters on adjacent atoms in **BPAM** molecules were restrained to be similar.

Supplementary Tables

Table S1. Crystal data and refinement parameters for complexes **1-*M***, **1-*P***, **2-*M***, **2-*P*** and **3-*M***.

Compound	**1-*M***	**1-*P***	**2-*M***	**2-*P***	**3-*M***
Empirical formula	$C_{77}H_{73}Ag_3Cl_6N_{22}O_{15}$	$C_{77}H_{73}Ag_3Cl_6N_{22}O_{15}$	$C_{78}H_{62}Ag_3Cl_{15}N_{22}O_{12}$	$C_{78}H_{62}Ag_3Cl_{15}N_{22}O_{12}$	$C_{78}H_{62}Ag_3Br_6Cl_9N_{22}O_{12}$
Formula weight	2082.88	2082.88	2354.86	2354.86	2621.62
Crystal system	Cubic	Cubic	Cubic	Cubic	Cubic
Space group	$I2_13$	$I2_13$	$I2_13$	$I2_13$	$I2_13$
a/Å	21.3062(3)	21.3212(3)	21.3930(4)	21.3239(4)	21.2844(3)
b/Å	21.3062(3)	21.3212(3)	21.3930(4)	21.3239(4)	21.2844(3)
c/Å	21.3062(3)	21.3212(3)	21.3930(4)	21.3239(4)	21.2844(3)
α/º	90	90	90	90	90
β/º	90	90	90	90	90
γ/º	90	90	90	90	90
V/Å ^{3}	9672.0(2)	9692.5(2)	9790.7(3)	9696.2(3)	9642.4(2)
Z	4	4	4	4	4
D_c/(g·cm^{-3})	1.430	1.427	1.598	1.613	1.806
T (K)	220(2)	220(2)	220(2)	220(2)	220(2)
F(000)	4208	4208	4704	4704	5136
Absorption coefficient/mm^{-1}	0.835	0.834	1.071	1.081	3.407
Reflections collected/unique	35408 / 3675	36053 / 3685	33168 / 3743	27746 / 3670	38059 / 3673
R(int)	0.0483	0.0443	0.0435	0.0620	0.0710
Data/restraints/parameters	3675 / 6 / 200	3685 / 6 / 200	3743 / 0 / 198	3670 / 0 / 198	3673 / 0 / 198
Goodness-of-fit on F^2	1.029	1.025	1.039	1.015	1.017
$R_1{}^a$ [I>2σ(I)]	0.0566	0.0546	0.0366	0.0546	0.0427
w$R_2{}^b$ [I>2σ(I)]	0.1492	0.1466	0.1046	0.1180	0.1100
$R_1{}^a$ (all data)	0.0731	0.0703	0.0429	0.0903	0.0612
w$R_2{}^b$ (all data)	0.1627	0.1606	0.1091	0.1336	0.1199
Flack parameter	-0.01(5)	-0.04(5)	0.04(3)	0.02(5)	0.034(15)
CCDC number	1417887	1417893	1448704	1448740	1449166

[a] $R_1 = \sum||F_o| - |F_c||/\sum|F_o|$

[b] $wR_2 = [\sum w(F_o^2 - F_c^2)^2/\sum w(F_o^2)^2]^{1/2}$

Table S2. Crystal data and refinement parameters for complexes ***3-P***, ***L*-BPAM@1-*M***, ***D*-BPAM@1-*P***, ***D*-BPAM@1-*M*** and ***L*-BPAM@1-*P***.

Compound	**3-*P***	***L*-BPAM@1-*M***	***D*-BPAM@1-*P***	***D*-BPAM@1-*M***	***L*-BPAM@1-*P***
Empirical formula	$C_{78}H_{62}Ag_3Br_6Cl_9N_{22}O_{12}$	$C_{83}H_{75}Ag_3Cl_3N_{21}O_{15}$	$C_{83}H_{75}Ag_3Cl_3N_{21}O_{15}$	$C_{83}H_{75}Ag_3Cl_3N_{21}O_{15}$	$C_{83}H_{75}Ag_3Cl_3N_{21}O_{15}$
Formula weight	2621.62	2036.60	2036.60	2036.60	2036.60
Crystal system	Cubic	Cubic	Cubic	Cubic	Cubic
Space group	$I2_13$	$I2_13$	$I2_13$	$I2_13$	$I2_13$
a/Å	21.3221(3)	21.5932(13)	21.5496(3)	21.6586(5)	21.6426(5)
b/Å	21.3221(3)	21.5932(13)	21.5496(3)	21.6586(5)	21.6426(5)
c/Å	21.3221(3)	21.5932(13)	21.5496(3)	21.6586(5)	21.6426(5)
α/°	90	90	90	90	90
β/°	90	90	90	90	90
γ/°	90	90	90	90	90
V/Å ^{3}	9693.7(2)	10068.2(10)	10007.3(2)	10159.9(4)	10137.4(4)
Z	4	4	4	4	4
D_c/(g·cm^{-3})	1.796	1.344	1.352	1.331	1.334
T (K)	220(2)	220(2)	220(2)	220(2)	220(2)
F(000)	5136	4128	4128	4128	4128
Absorption coefficient/mm^{-1}	3.389	0.724	0.728	0.717	0.719
Reflections collected/unique	37153 / 3668	27952 / 2952	29235 / 2941	25307 / 2982	29312 / 2986
R(int)	0.0512	0.0582	0.0541	0.0989	0.0907
Data/restraints/parameters	3668 / 0 / 198	2952 / 58 / 223	2941 / 62 / 223	2982 / 62 / 223	2986 / 62 / 223
Goodness-of-fit on F^2	1.073	1.058	1.075	1.048	1.068
R_1[a] [I>2σ(I)]	0.0373	0.0665	0.0722	0.0785	0.0774
wR_2[b] [I>2σ(I)]	0.1085	0.1696	0.1917	0.1987	0.1976
R_1[a] (all data)	0.0466	0.1041	0.0952	0.1182	0.1099
wR_2[b] (all data)	0.1155	0.1870	0.2093	0.2215	0.2193
Flack parameter	0.048(13)	0.01(7)	0.07(7)	0.07(8)	0.03(8)
CCDC number	1449160	1417911	1457367	1457368	1457369

[a] $R_1 = \sum||F_o| - |F_c||/\sum|F_o|$

[b] $wR_2 = [\sum w(F_o^2 - F_c^2)^2/\sum w(F_o^2)^2]^{1/2}$

==

Table S3. A summary of structure determinations of six randomly selected crystals for **1** grown in the absence of chiral templates: Cell parameters, R factors, Flack absolute structure parameters for each refinement are given.

SN	Space group	*a* (Å)	V (Å^3)	R_1	wR_2	Flack parameter	Configuration of silver (I)	CCDC No.
1	$I2_13$	21.3203(3)	9691.3(2)	0.0566	0.1635	-0.05(5)	*P*	1417905
2	$I2_13$	21.3457(3)	9725.9(2)	0.0583	0.1658	0.01(5)	*M*	1417906
3	$I2_13$	21.3361(4)	9712.8(3)	0.0600	0.1775	-0.03(5)	*P*	1417907
4	$I2_13$	21.2948(3)	9656.5(2)	0.0598	0.1714	-0.05(6)	*M*	1417908
5	$I2_13$	21.3220(3)	9693.6(2)	0.0619	0.1717	-0.01(5)	*M*	1417909
6	$I2_13$	21.3474(4)	9728.3(3)	0.0646	0.1967	0.02(6)	*P*	1417910

Table S4. A summary of structure determinations of six randomly selected crystals for **1-*M*** grown in the presence of ***L*-BPAM**: Cell parameters, R factors, Flack absolute structure parameters for each refinement are given.

SN	Space group	*a* (Å)	V (Å^3)	R_1	wR_2	Flack parameter	Configuration of silver (I)	CCDC No.
1	$I2_13$	21.3062(3)	9672.0(2)	0.0566	0.1627	-0.01(5)	*M*	1417887
2	$I2_13$	21.2987(3)	9661.8(2)	0.0589	0.1661	0.00(5)	*M*	1417888
3	$I2_13$	21.3054(3)	9671.0(2)	0.0616	0.1726	0.00(6)	*M*	1417889
4	$I2_13$	21.3483(4)	9729.5(3)	0.0659	0.1840	0.03(6)	*M*	1417890
5	$I2_13$	21.3000(5)	9663.6(4)	0.0667	0.1757	0.01(6)	*M*	1417891
6	$I2_13$	21.4745(11)	9903.1(9)	0.0695	0.2019	0.01(6)	*M*	1417892

Table S5. A summary of structure determinations of six randomly selected crystals for **1-*P*** grown in the presence of ***D*-BPAM**: Cell parameters, R factors, Flack absolute structure parameters for each refinement are given.

SN	Space group	*a* (Å)	V (Å^3)	R_1	wR_2	Flack parameter	Configuration of silver (I)	CCDC No.
1	$I2_13$	21.3212(3)	9692.5(2)	0.0546	0.1606	-0.04(5)	*P*	1417893
2	$I2_13$	21.3496(3)	9731.3(2)	0.0594	0.1724	-0.07(5)	*P*	1417894
3	$I2_13$	21.2904(3)	9650.5(2)	0.0601	0.1703	-0.05(5)	*P*	1417895
4	$I2_13$	21.3038(3)	9668.8(2)	0.0602	0.1705	-0.06(5)	*P*	1417896
5	$I2_13$	21.3228(3)	9694.7(2)	0.0618	0.1756	-0.04(6)	*P*	1417897
6	$I2_13$	21.3193(3)	9689.9(2)	0.0621	0.1737	-0.01(6)	*P*	1417898

Table S6. A summary of structure determinations of six randomly selected crystals for **2-*M*** grown in the presence of ***L*-BPAM**: Cell parameters, R factors, Flack absolute structure parameters for each refinement are given.

SN	Space group	*a* (Å)	V ($Å^3$)	R_1	wR_2	Flack parameter	Configuration of silver (I)	CCDC No.
1	$I2_13$	21.3930(4)	9790.7(3)	0.0366	0.1091	0.04(3)	*M*	1448704
2	$I2_13$	21.3180(3)	9688.1(2)	0.0466	0.1217	-0.01(4)	*M*	1448705
3	$I2_13$	21.3281(11)	9701.9(9)	0.0501	0.1162	0.00(4)	*M*	1448706
4	$I2_13$	21.3446(3)	9724.4(2)	0.0500	0.1260	-0.04(4)	*M*	1448707
5	$I2_13$	21.3528(3)	9735.6(2)	0.0552	0.1319	-0.01(4)	*M*	1448708
6	$I2_13$	21.3847(5)	9779.3(4)	0.0568	0.1351	-0.03(5)	*M*	1448709

Table S7. A summary of structure determinations of six randomly selected crystals for **2-*P*** grown in the presence of ***D*-BPAM**: Cell parameters, R factors, Flack absolute structure parameters for each refinement are given.

SN	Space group	*a* (Å)	V ($Å^3$)	R_1	wR_2	Flack parameter	Configuration of silver (I)	CCDC No.
1	$I2_13$	21.3239(4)	9696.2(3)	0.0546	0.1336	0.02(5)	*P*	1448740
2	$I2_13$	21.3432(3)	9722.5(2)	0.0666	0.1653	-0.02(6)	*P*	1448741
3	$I2_13$	21.2788(5)	9634.8(4)	0.0555	0.1307	0.00(5)	*P*	1448742
4	$I2_13$	21.3154(4)	9684.6(3)	0.0597	0.1425	-0.01(5)	*P*	1448743
5	$I2_13$	21.3288(4)	9702.8(3)	0.0542	0.1308	-0.04(5)	*P*	1448744
6	$I2_13$	21.3018(4)	9666.0(3)	0.0573	0.1496	0.01(5)	*P*	1448745

Table S8. A summary of structure determinations of six randomly selected crystals for **3-*M*** grown in the presence of ***L*-BPAM**: Cell parameters, R factors, Flack absolute structure parameters for each refinement are given.

SN	Space group	*a* (Å)	V ($Å^3$)	R_1	wR_2	Flack parameter	Configuration of silver (I)	CCDC No.
1	$I2_13$	21.2844(3)	9642.4(2)	0.0427	0.1199	0.034(15)	*M*	1449166
2	$I2_13$	21.160(16)	9474.3(2)	0.1185	0.2948	0.07(5)	*M*	1449167
3	$I2_13$	21.287(2)	9645.9(2)	0.1032	0.2570	0.06(4)	*M*	1449168
4	$I2_13$	21.3238(3)	9696.0(2)	0.0344	0.1074	0.05(12)	*M*	1449169
5	$I2_13$	21.154(2)	9466.9(2)	0.1079	0.2839	0.07(4)	*M*	1449170
6	$I2_13$	21.2804(4)	9636.9(3)	0.0461	0.1202	0.022(15)	*M*	1449171

Table S9. A summary of structure determinations of six randomly selected crystals for **3-*P*** grown in the presence of ***D*-BPAM**: Cell parameters, R factors, Flack absolute structure parameters for each refinement are given.

SN	Space group	*a* (Å)	V ($Å^3$)	R_1	wR_2	Flack parameter	Configuration of silver (I)	CCDC No.
1	$I2_13$	21.3221(3)	9693.7(2)	0.0373	0.1155	0.048(13)	*P*	1449160
2	$I2_13$	21.3186(11)	9688.9(9)	0.0372	0.1147	0.061(13)	*P*	1449161
3	$I2_13$	21.3189(3)	9689.3(2)	0.0366	0.1150	0.058(13)	*P*	1449162
4	$I2_13$	21.2287(5)	9566.9(4)	0.0787	0.2828	0.110(4)	*P*	1449163
5	$I2_13$	21.3154(3)	9684.6(2)	0.0355	0.1109	0.029(13)	*P*	1449164
6	$I2_13$	21.3139(3)	9682.5(2)	0.0432	0.1294	0.010(16)	*P*	1449165

Table S10. Catalytic results of five parallel cycloaddition reactions between **PGE** and methyl acrylate catalyzed by **1** randomly collected from five isolated crystallization batches.

Entry	Conversion	*ee*
a	84%	-26%
b	85%	38%
c	80%	27%
d	87%	-11%
e	81%	-45%

Table S11. The catalytic results of the tandem systems of **2+*L*-BPAM** and **3+*L*-BPAM**.

**2+*L*-BPAM**			**3+*L*-BPAM**		
Entry	Conversion	*ee*	Entry	Conversion	*ee*
a	89%	51%	f	90%	60%
b	92%	53%	g	89%	56%
c	87%	47%	h	91%	54%
d	91%	52%	i	90%	67%
e	92%	60%	j	86%	59%

Table S12. The catalytic results of the tandem systems of **2+*D*-BPAM** and **3+*D*-BPAM**.

**2+*D*-BPAM**			**3+*D*-BPAM**		
Entry	Conversion	*ee*	Entry	Conversion	*ee*
a	90%	-57%	f	87%	-58%
b	88%	-54%	g	91%	-64%
c	89%	-49%	h	89%	-60%
d	86%	-52%	i	88%	-55%
e	91%	-58%	j	92%	-59%

Supplementary Figures

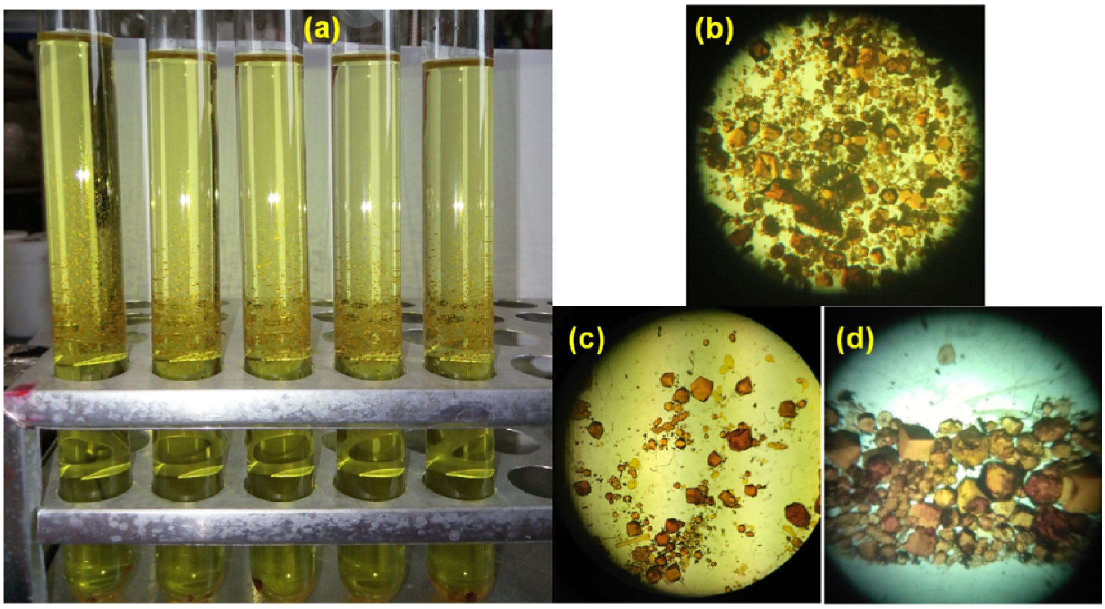

Figure S1. (a) The crystallization of Ag(I)-based MOFs *via* solution-layering diffusion. Optical microscope images of single crystals for **1** (b), **2** (c) and **3** (d).

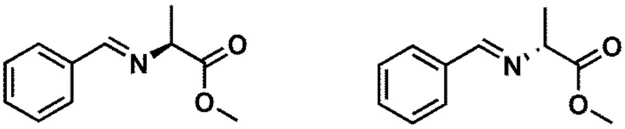

Figure S2. Structural formula of (*S*,*E*)-methyl-2-(benzylideneamino)propanoate (***L*-BPAM**) and (*R*,*E*)-methyl-2-(benzylideneamino)propanoate (***D*-BPAM**).

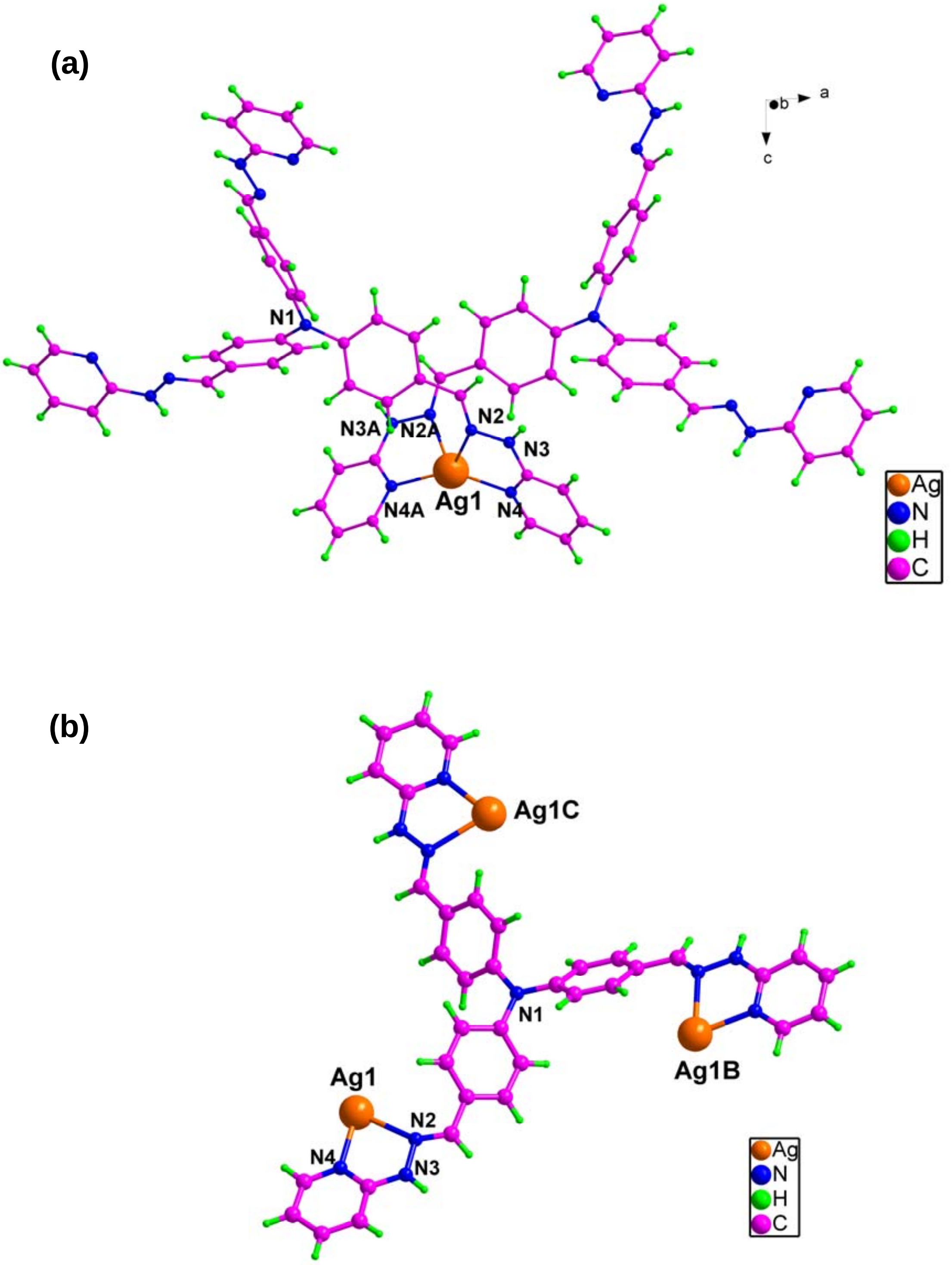

Figure S3. Coordination environments of the silver(I) ion (a) and $\mathbf{L_1}$ ligand (b) in **1**. Symmetry code: A, *-x, -y+1/2, z*; B: *x+1/2, y-1/2, z-1/2*; C, *-x, y+1/2, -z+1/2*.

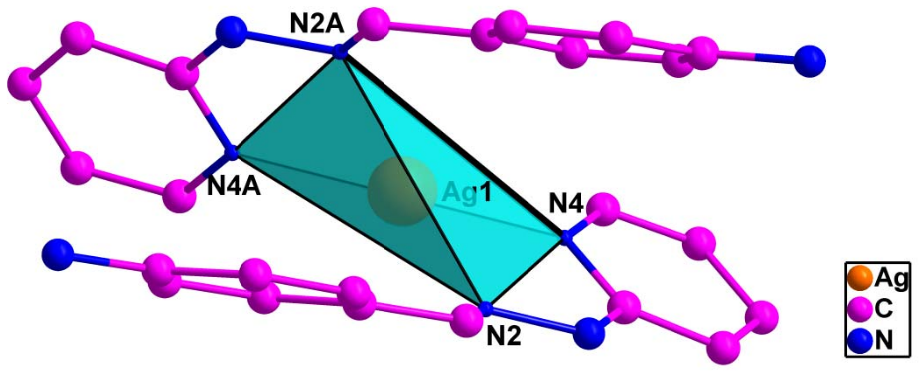

Figure S4. The distorted tetrahedral coordination geometry of Ag(I) ion with selected labelling scheme in **1** (Symmetry code**:** A *-x+5/2, y, -z*). In the tetrahedron, the crystallographically independent Ag(1) ion is coordinated by two nitrogen atoms from one $\mathbf{L_1}$ ligand [Ag(1)-N(4) 2.197(4) Å, Ag(1)-N(2) 2.429(3) Å] and two equivalent nitrogen atoms from another $\mathbf{L_1}$ ligand. Selected bond angle: N(4A)-Ag(1)-N(4) 147.25(19)º, N(4A)-Ag(1)-N(2) 132.96(14)º, N(4)-Ag(1)-N(2) 72.17(13)º, N(2)-Ag(1)-N(2A) 96.53(16)º.

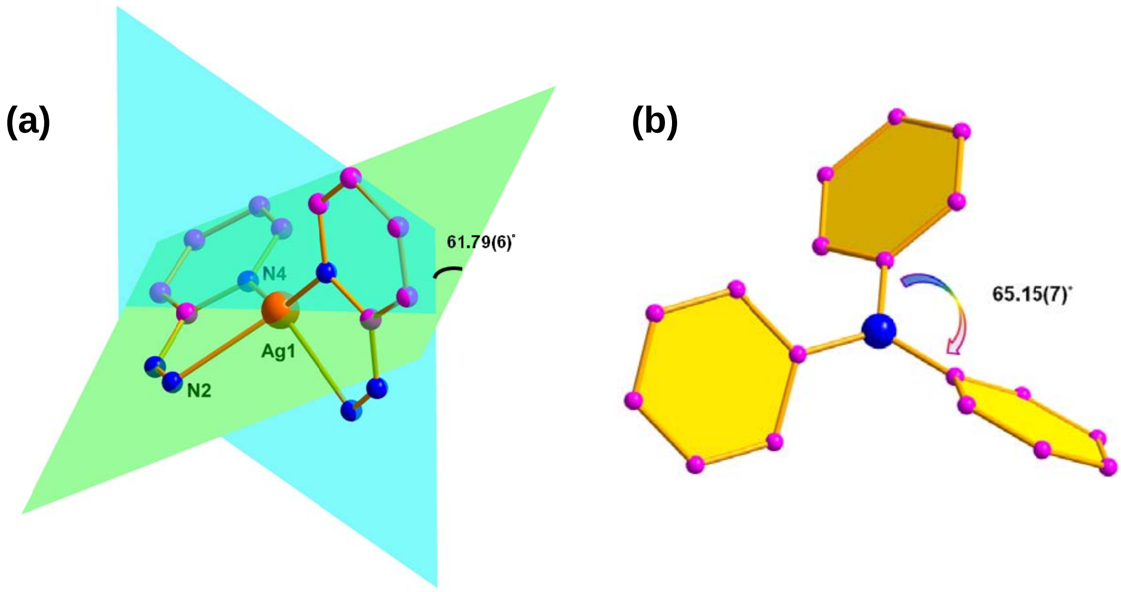

Figure S5. (a) The dihedral angle [61.79(6)º] between the bidentate chelating planes in **1**. (b) The dihedral angle [65.15(7)º] between the two phenyl rings of triphenylamine moiety in **1**.

(a)

(b)

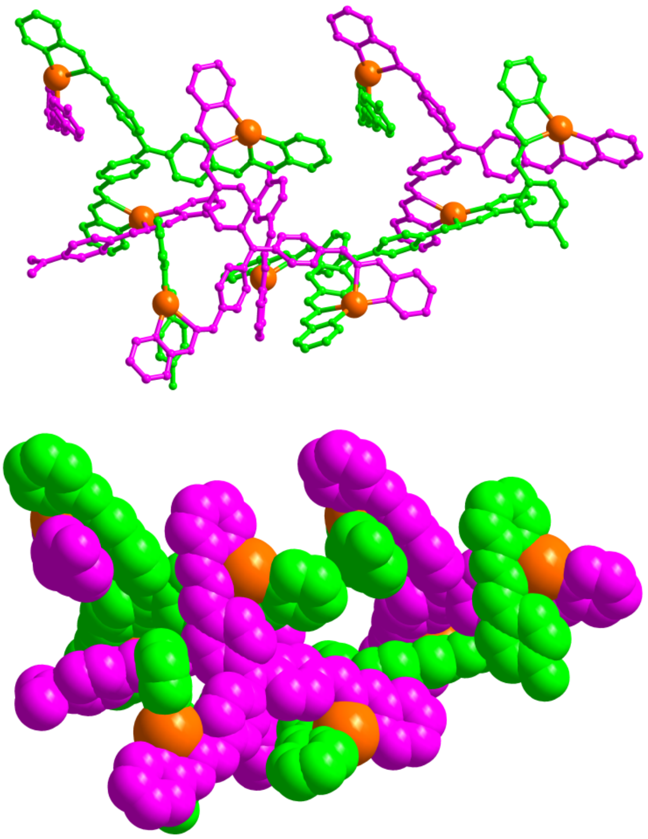

Figure S6. Helical moieties in **1** shown in Ball-Stick mode (a) and Spacefill mode (b), respectively.

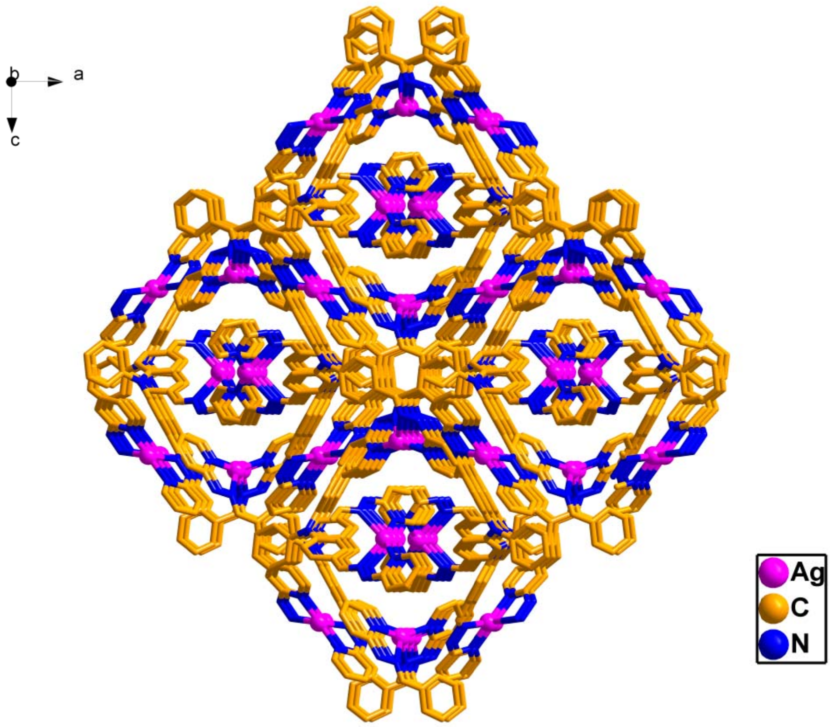

Figure S7. 3D framework of **1**. All H atoms, counter ions and solvent molecules are omitted for clarity.

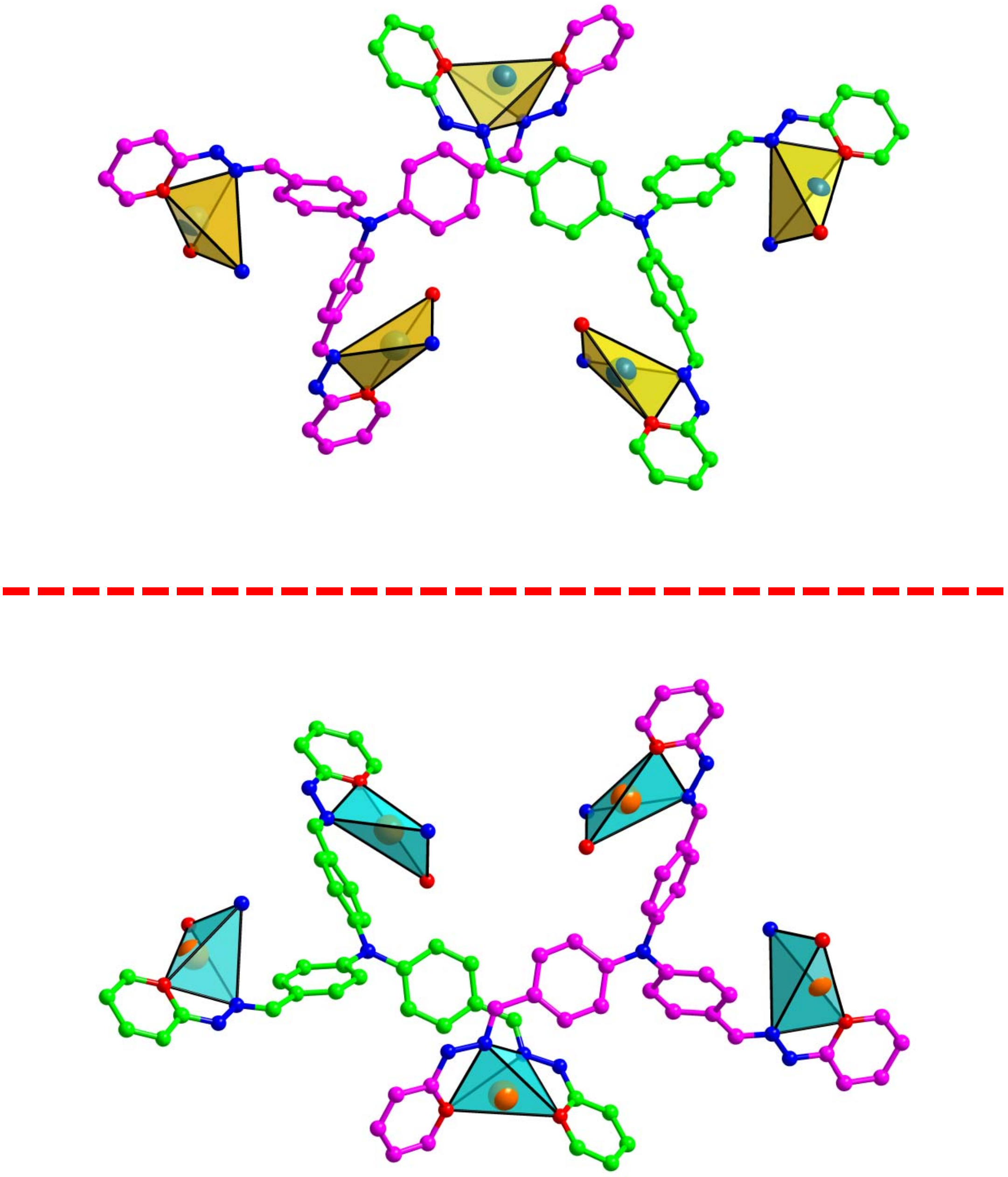

Figure S8. The mirror relationship chart of **1−*M*** (up) and **1−*P*** (down), showing the distorted tetrahedral silver(I) centers, red balls represent the pyridine N atoms.

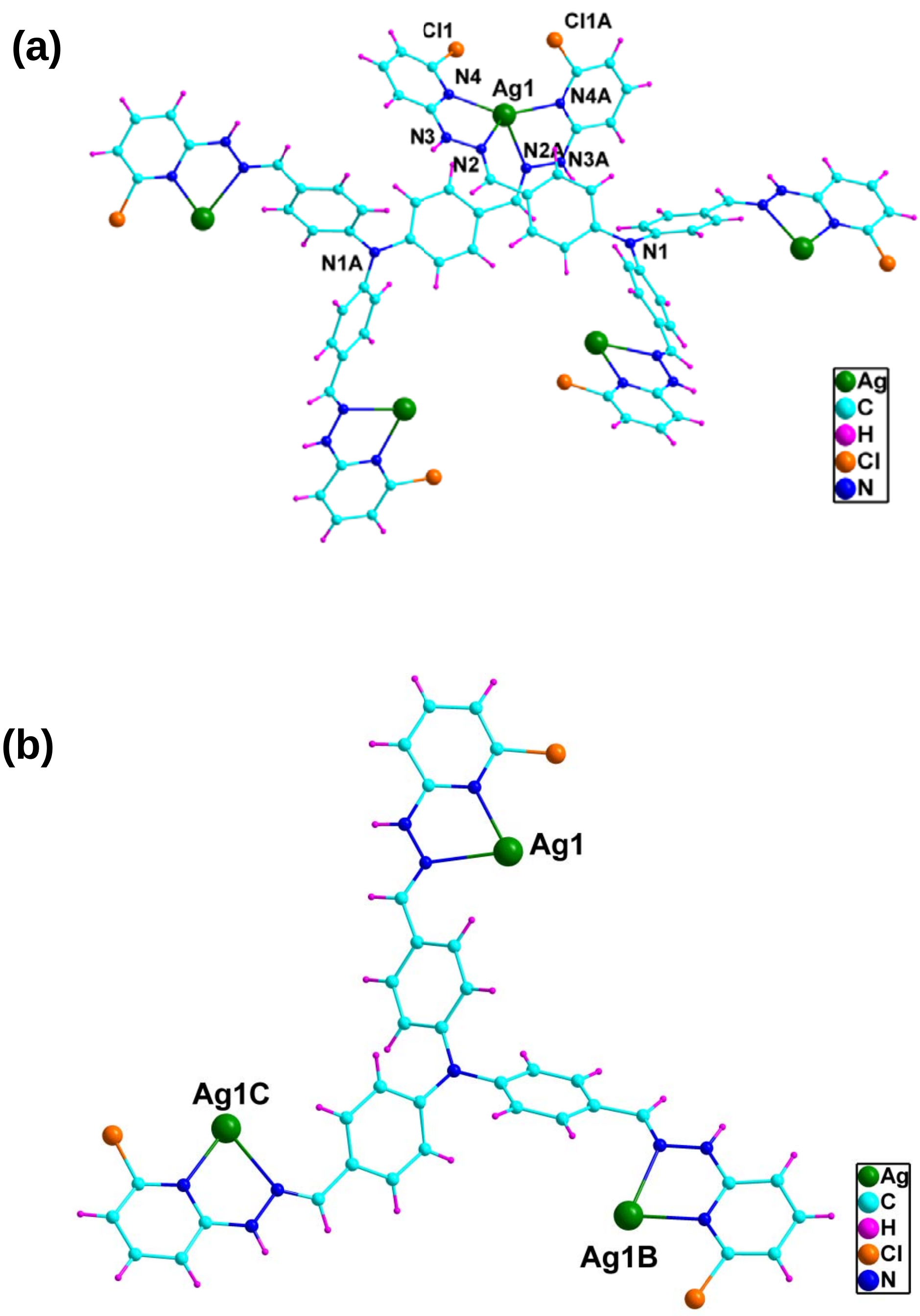

Figure S9. Coordination environments of silver(I) (a) and $\mathbf{L_2}$ ligand (b) in **2**. Selected bond lengths and angles: Ag(1)-N(4) 2.227(3) Å, Ag(1)-N(2) 2.434(3) Å; N(4A)-Ag(1)-N(4) 148.07(15)º, N(4A)-Ag(1)-N(2) 132.92(10)º, N(4)-Ag(1)-N(2) 71.93(9)º, N(2)-Ag(1)-N(2A) 95.26(11)º. Symmetry code: A, *x+1/2, y-1/2, z-1/2*; B: *x+1/2, -y+3/2, -z+2*; C, *x+1/2, y+1/2, z-1/2*.

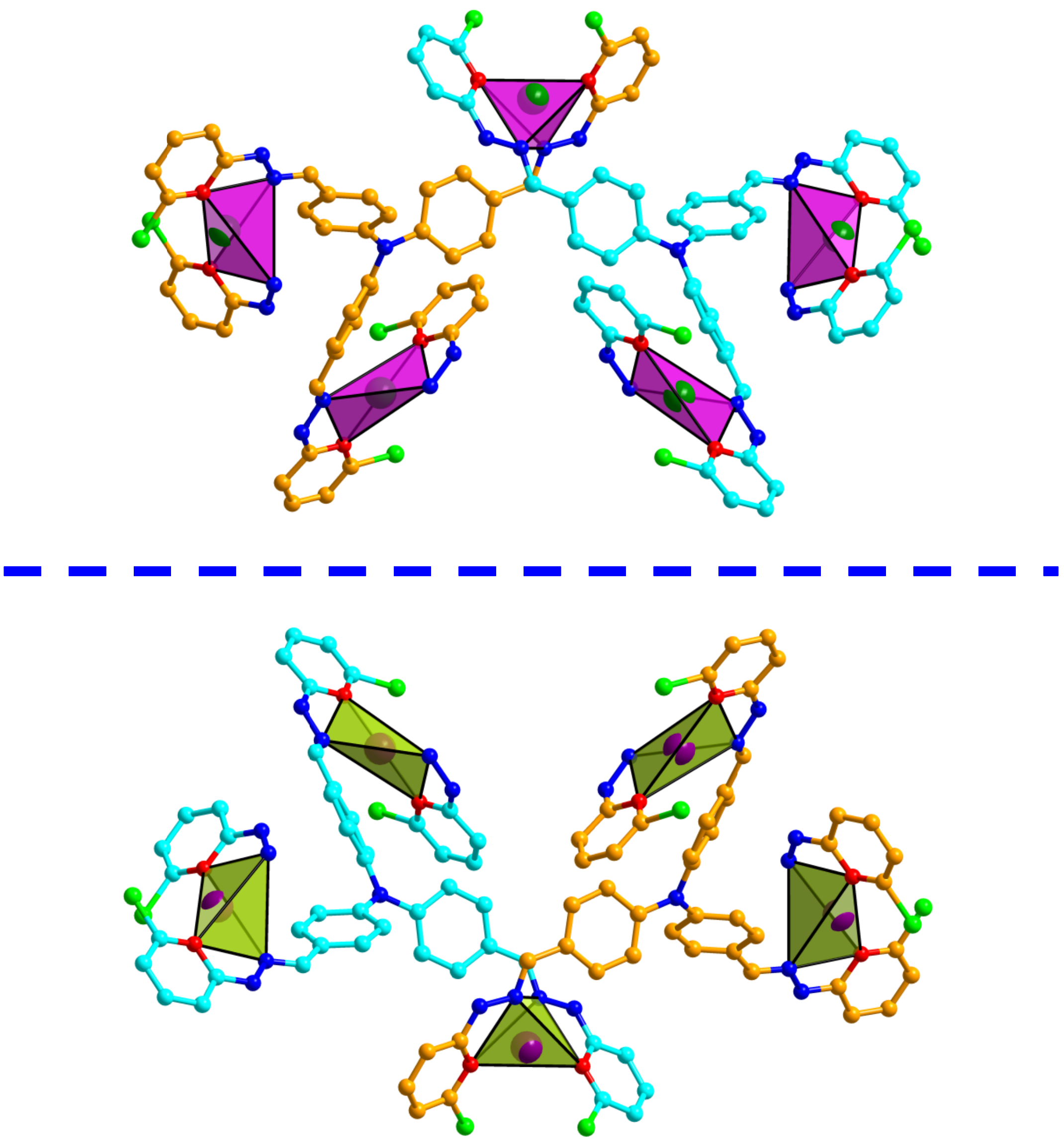

Figure S10. The mirror image structures of **2−*M*** (up) and **2−*P*** (down), showing the distorted tetrahedral silver(I) centers. Red balls: pyridine N atoms; bright green balls: chloro atoms.

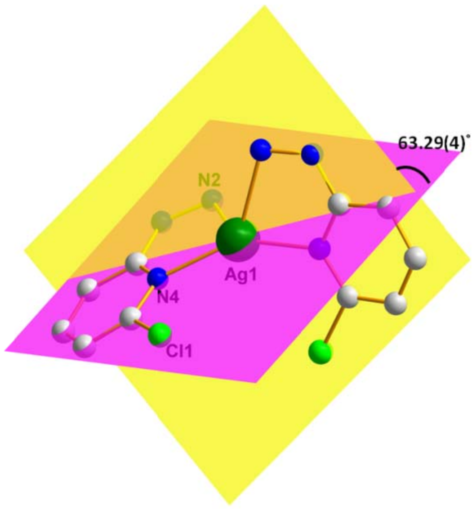

Figure S11. The dihedral angle [63.29(4)º] between the bidentate chelating planes in **2**.

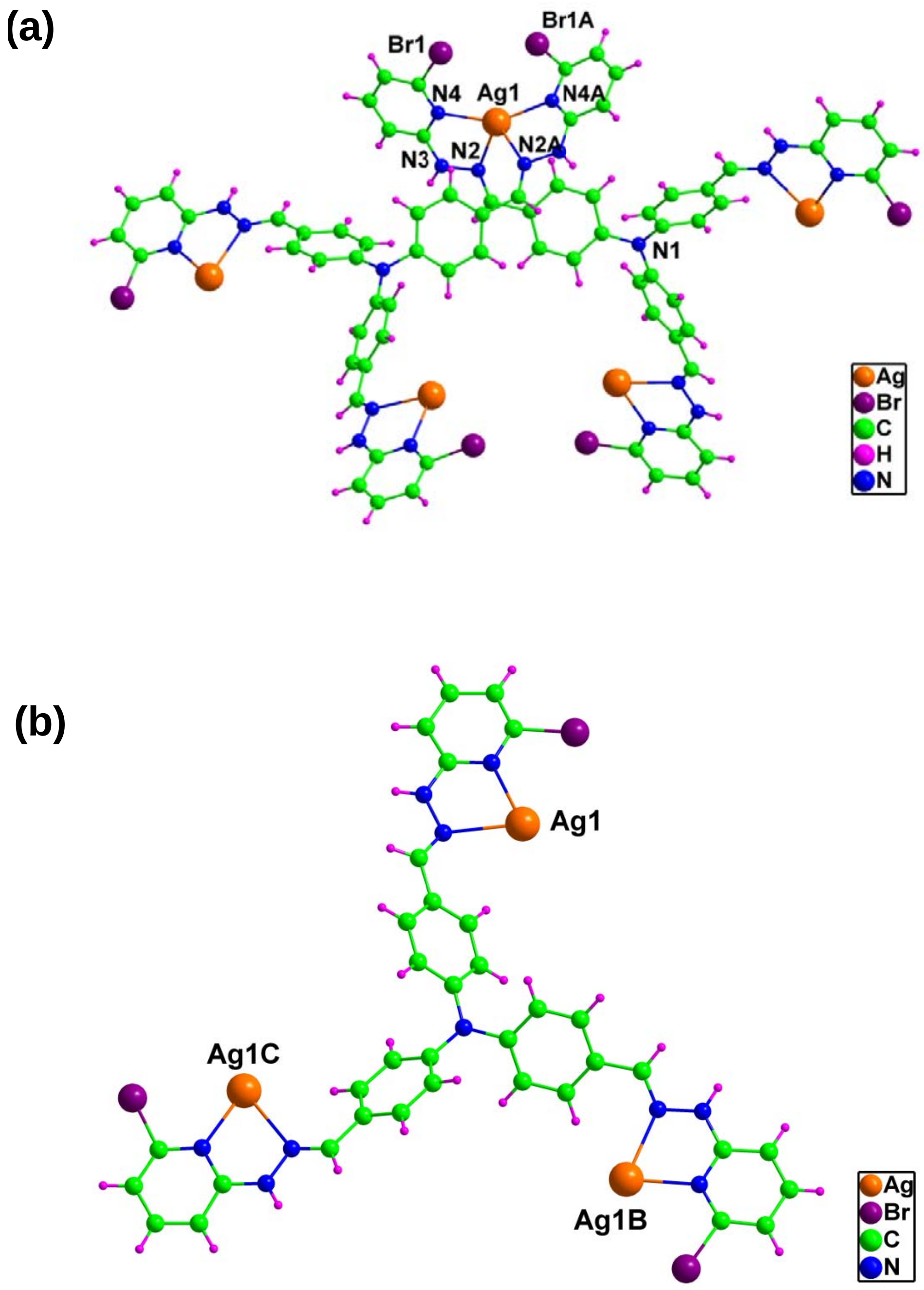

Figure S12. Coordination environments of silver(I) (a) and $\mathbf{L_3}$ ligand (b) in **3**. Selected bond lengths and angles: Ag(1)-N(4) 2.238(3) Å, Ag(1)-N(2) 2.439(3) Å; N(4A)-Ag(1)-N(4) 150.8(2)º, N(4A)-Ag(1)-N(2) 131.15(12)º, N(4)-Ag(1)-N(2) 71.71(12)º, N(2)-Ag(1)-N(2A) 93.89(16)º. Symmetry code: A, *-x+1/2, y, -z+1*; B: *x+1/2, y+1/2, z-1/2*; C, *-x, -y+1/2, z*.

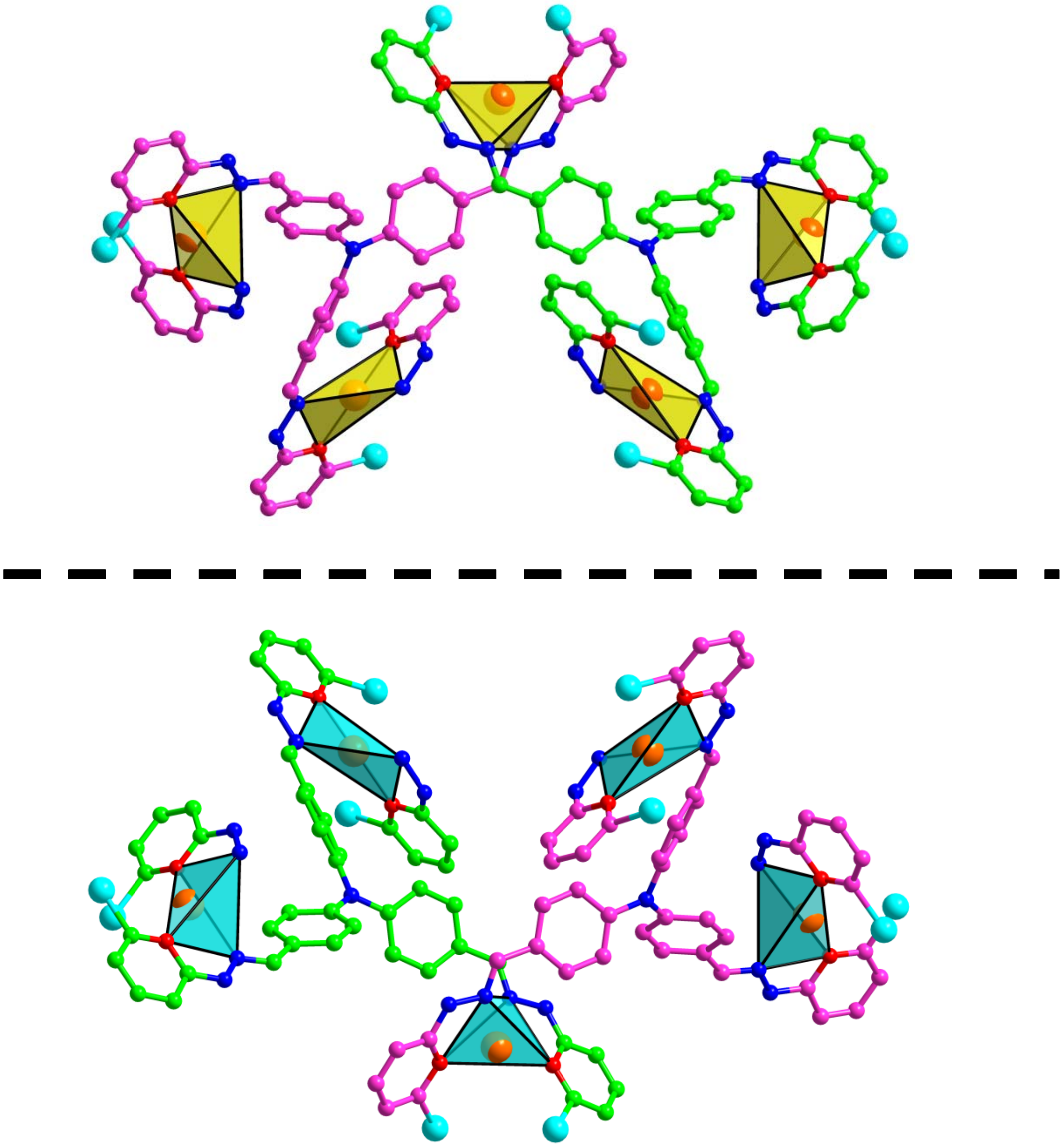

Figure S13. The mirror image structures of **3−*M*** (up) and **3−*P*** (down), showing the distorted tetrahedral silver(I) centers. Red balls: pyridine N atoms; turquiose balls: bromo atoms.

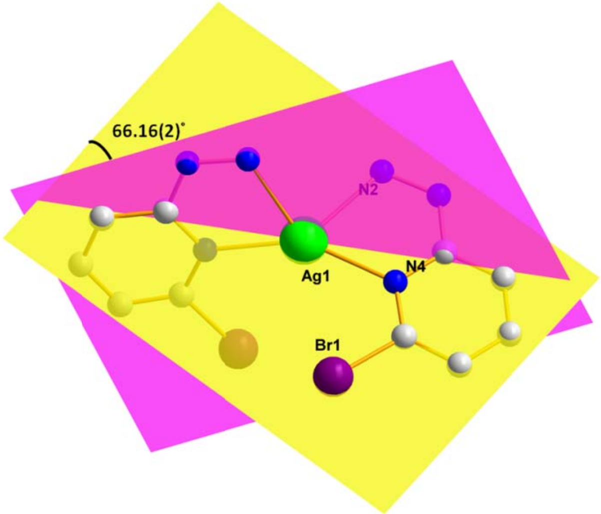

Figure S14. The dihedral angle [66.16(2)º] between the bidentate chelating planes in **3**.

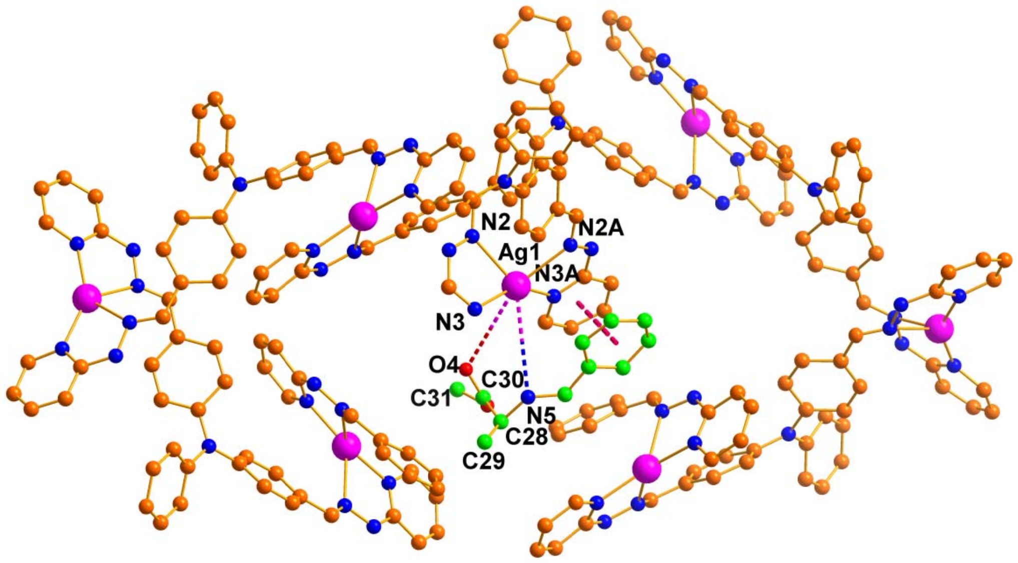

Figure S15. Ball-and-stick representation of the crystal structure of **1-*M*** impregnated ***L*-BPAM** molecule with selected labeling scheme, displaying potential interactions between the framework and the substrate molecules adsorbed. Selected bond distances (Å) and angle (°): Ag(1)−N(2) 2.464(3), Ag(1)−N(3) 2.198(4), C(30)-O(4) 1.346(9); N(3A)-Ag(1)-N(3) 146.6(2), N(3A)-Ag(1)-N(2A) 71.96(14), N(3)-Ag(1)-N(2A) 134.85(15), N(2A)-Ag(1)-N(2) 93.04(15). Potential intermolecular interactions: Ag(1)−N(5) 4.108(4) Å, Ag(1)−O(4) 3.835(3) Å; π-π stacking interaction between ***L*-BPAM** and **1-*M***: Cg…Cg = 3.40 Å. (Symmetry code**:** A *3/2-x, y, 1-z*).

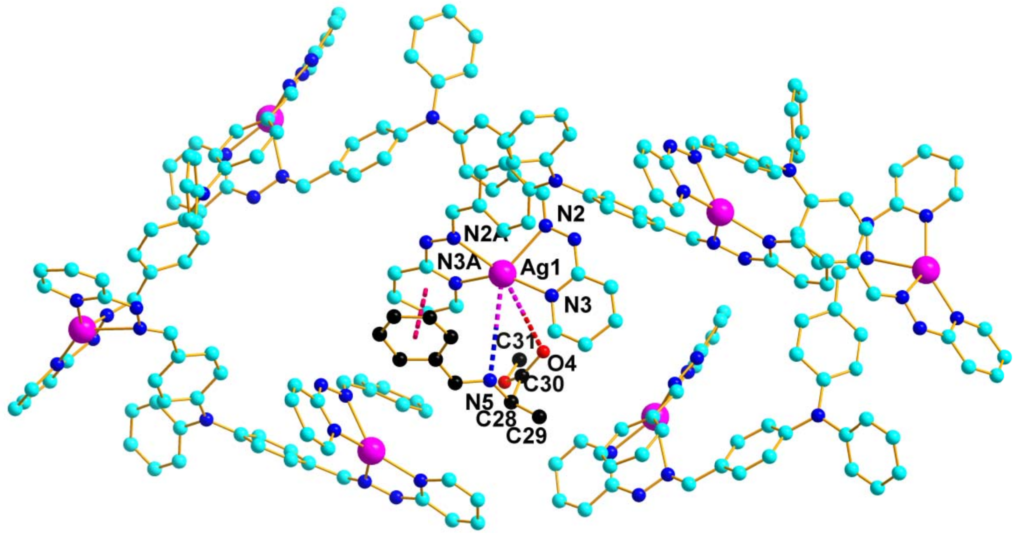

Figure S16. Ball-and-stick representation of the crystal structure of **1-*P*** impregnated ***D*-BPAM** molecule with selected labeling scheme, displaying potential interactions between the framework and the substrate molecules adsorbed. Selected bond distances (Å) and angle (°): Ag(1)−N(2) 2.457(4), Ag(1)−N(3) 2.211(4), C(30)-O(4) 1.350(9); N(3A)-Ag(1)-N(3) 147.0(2), N(3A)-Ag(1)-N(2A) 71.68(14), N(3)-Ag(1)-N(2A) 134.65(15), N(2A)-Ag(1)-N(2) 93.66(17). Potential intermolecular interactions: Ag(1)−N(5) 4.143(4) Å, Ag(1)−O(4) 3.892(3) Å; π-π stacking interaction between ***D*-BPAM** and **1-*P***: Cg…Cg = 3.437 Å. (Symmetry code**:** A *1/2-x, y, 1-z*).

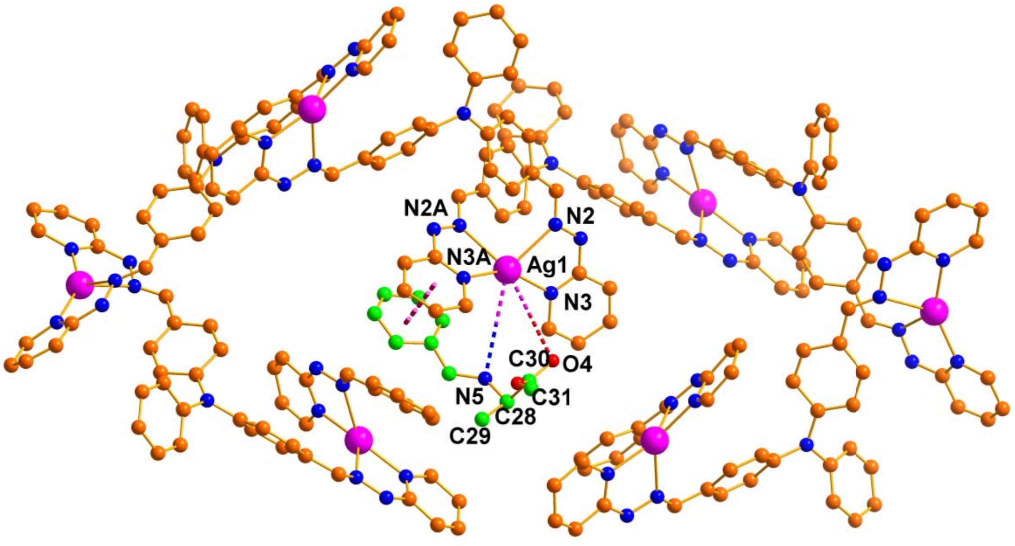

Figure S17. Ball-and-stick representation of the crystal structure of **1-*M*** impregnated ***D*-BPAM** molecule with selected labeling scheme, displaying potential interactions between the framework and the substrate molecules adsorbed. Selected bond distances (Å) and angle (°): Ag(1)−N(2) 2.473(4), Ag(1)−N(3) 2.206(5), C(30)-O(4) 1.346(9); N(3A)-Ag(1)-N(3) 148.4(2), N(3A)-Ag(1)-N(2A) 71.38(16), N(3)-Ag(1)-N(2A) 134.07(16), N(2A)-Ag(1)-N(2) 92.79(19). Potential intermolecular interactions: Ag(1)−N(5) 4.146(4) Å, Ag(1)−O(4) 3.984(3) Å; π-π stacking interaction between ***D*-BPAM** and **1-*M***: Cg…Cg = 3.441 Å. (Symmetry code**:** A *3/2-x, y, 1-z*).

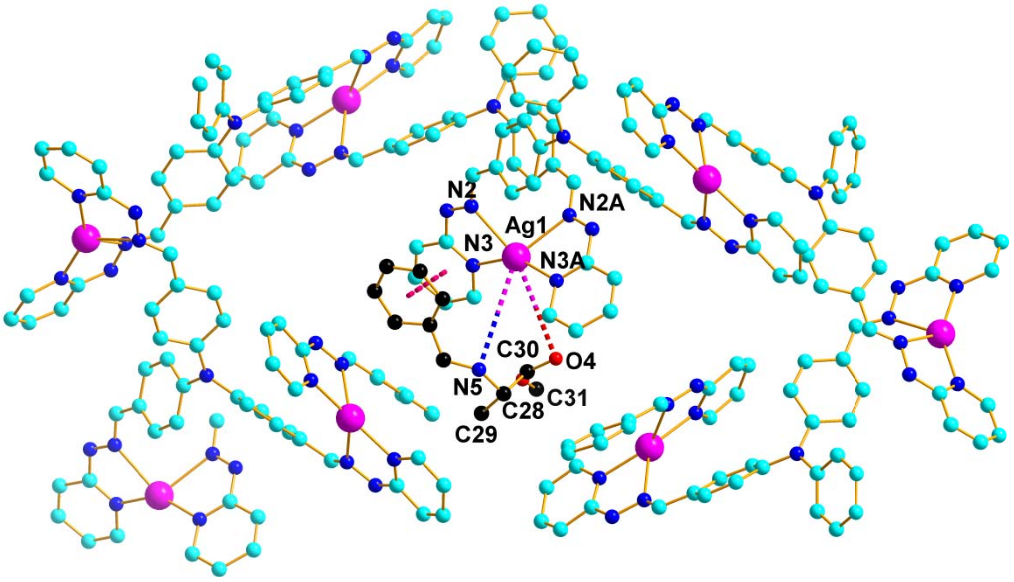

Figure S18. Ball-and-stick representation of the crystal structure of **1-*P*** impregnated ***L*-BPAM** molecule with selected labeling scheme, displaying potential interactions between the framework and the substrate molecules adsorbed. Selected bond distances (Å) and angle (°): Ag(1)−N(2) 2.483(4), Ag(1)−N(3) 2.209(5), C(30)-O(4) 1.349(9); N(3A)-Ag(1)-N(3) 148.6(2), N(3A)-Ag(1)-N(2A) 70.98(16), N(3)-Ag(1)-N(2A) 134.20(16), N(2A)-Ag(1)-N(2) 93.34(18). Potential intermolecular interactions: Ag(1)−N(5) 4.350(4) Å, Ag(1)−O(4) 4.040(2) Å; π-π stacking interaction between ***L*-BPAM** and **1-*P***: Cg…Cg = 3.493 Å. (Symmetry code**:** A *1/2-x, y, 1-z*).

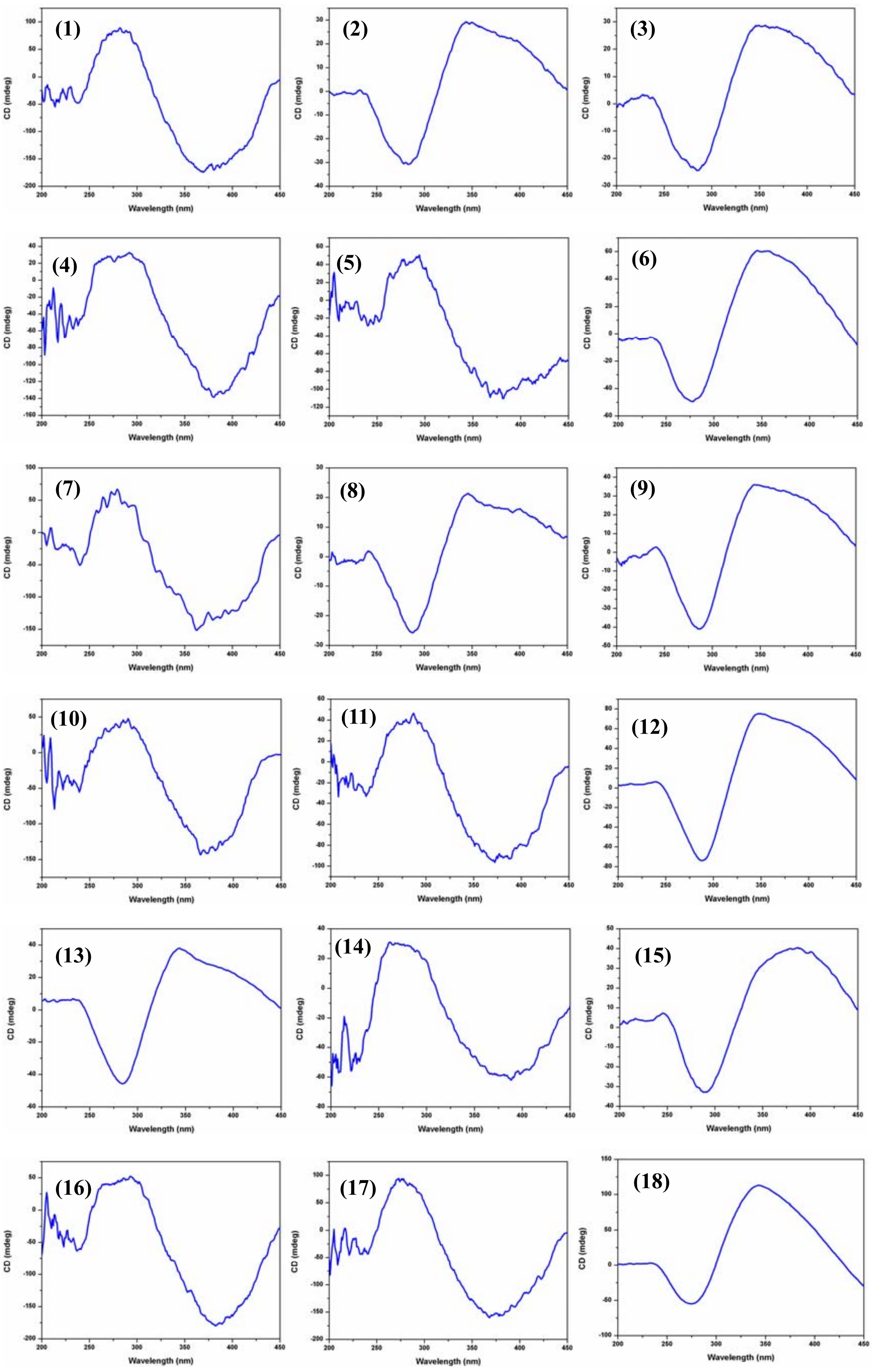
(1)
(2)
(3)
(4)
(5)
(6)
(7)
(8)
(9)
(10)
(11)
(12)
(13)
(14)
(15)
(16)
(17)
(18)
CD (mdeg)
Wavelength (nm)

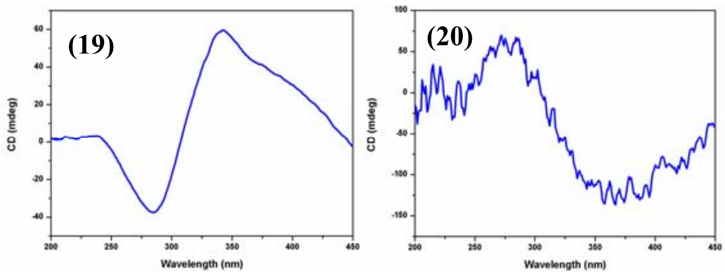

Figure S19. Solid state CD spectra for 20 bulk samples of **1** from twenty independent crystallization batches.

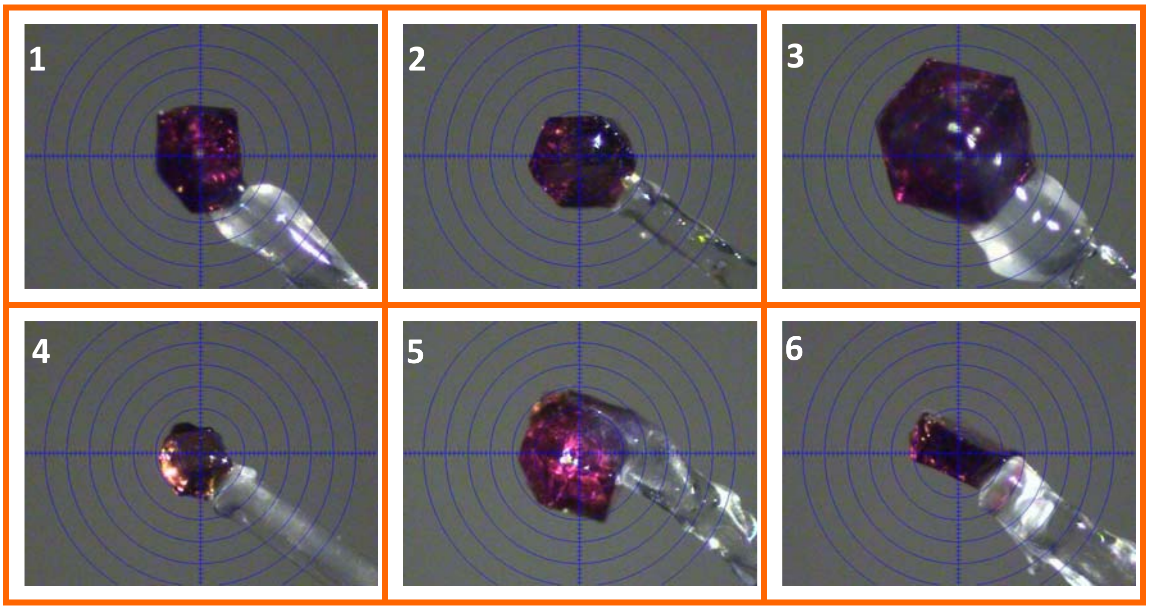

Figure S20. Photographs of six randomly selected crystals for **1** grown in the absence of chiral templates and used for X-ray data collection.

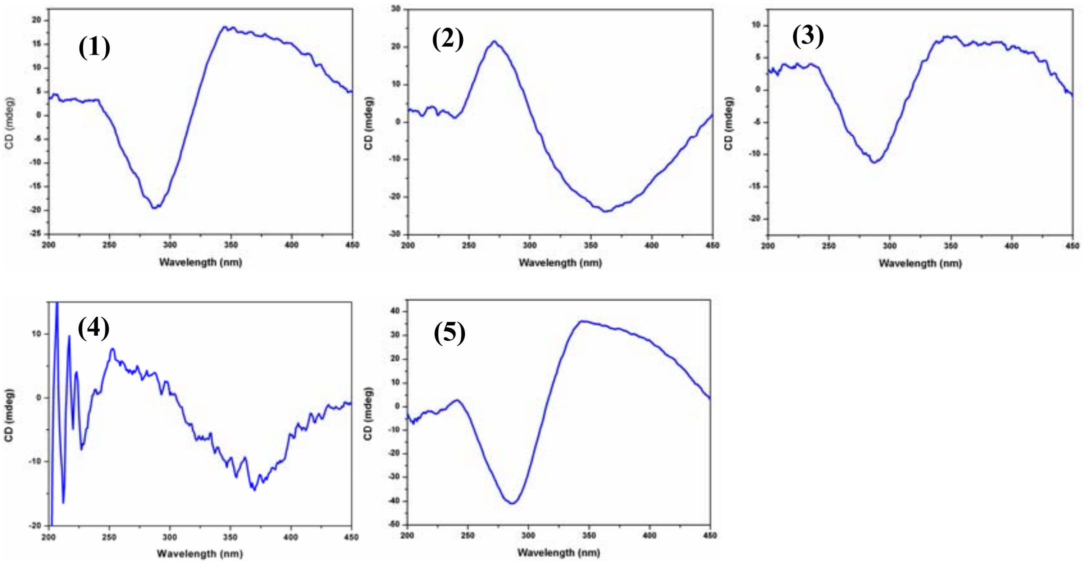

Figure S21. Solid state CD spectra of compound **1** used to catalyze the cycloaddition reactions between ***L*-BPAM** and methyl acrylate (the crystalline solids of **1** was randomly collected from five isolated crystallization batches).

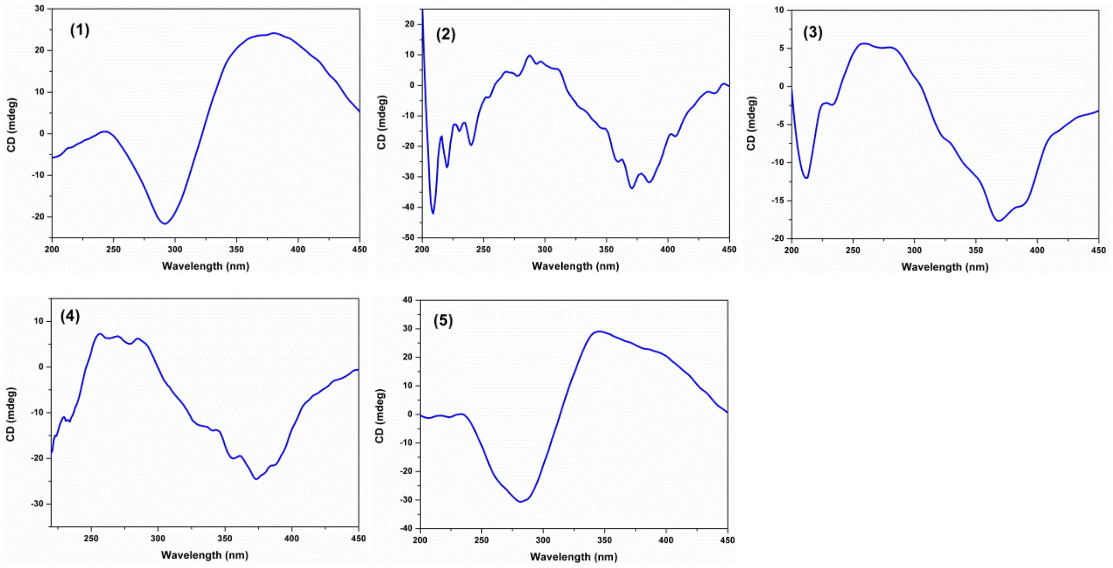

Figure S22. Solid state CD spectra of compound **1** used to catalyze the cycloaddition reactions between ***D*-BPAM** and methyl acrylate (the crystalline solids of **1** was randomly collected from five isolated crystallization batches).

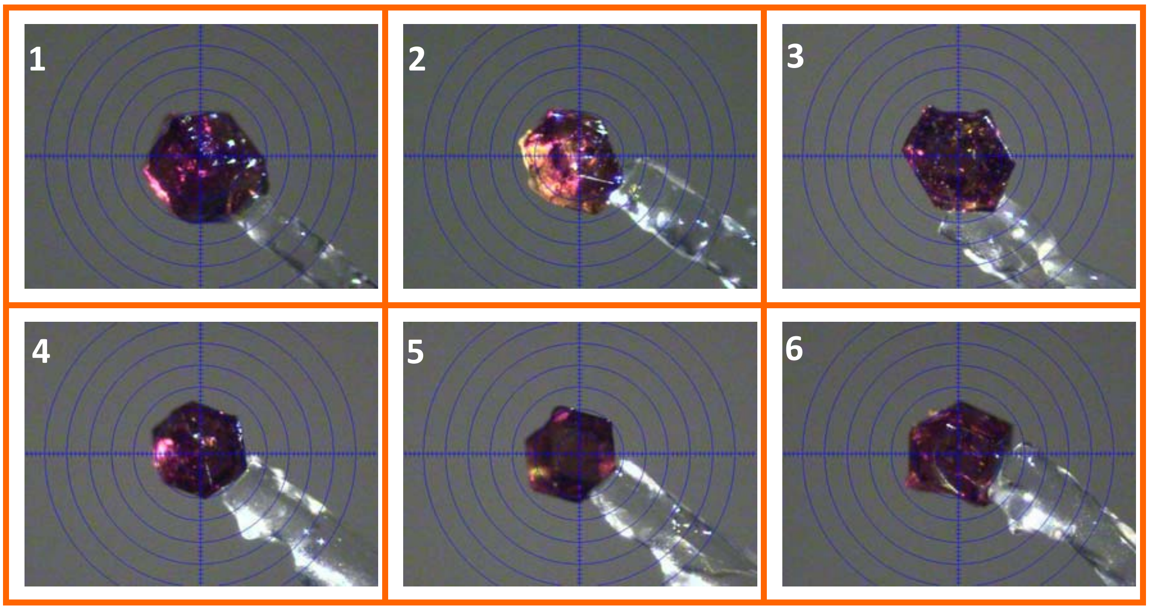

Figure S23. Photographs of six randomly selected crystals for **1-*M*** grown in the presence of ***L*-BPAM** and used for X-ray data collection.

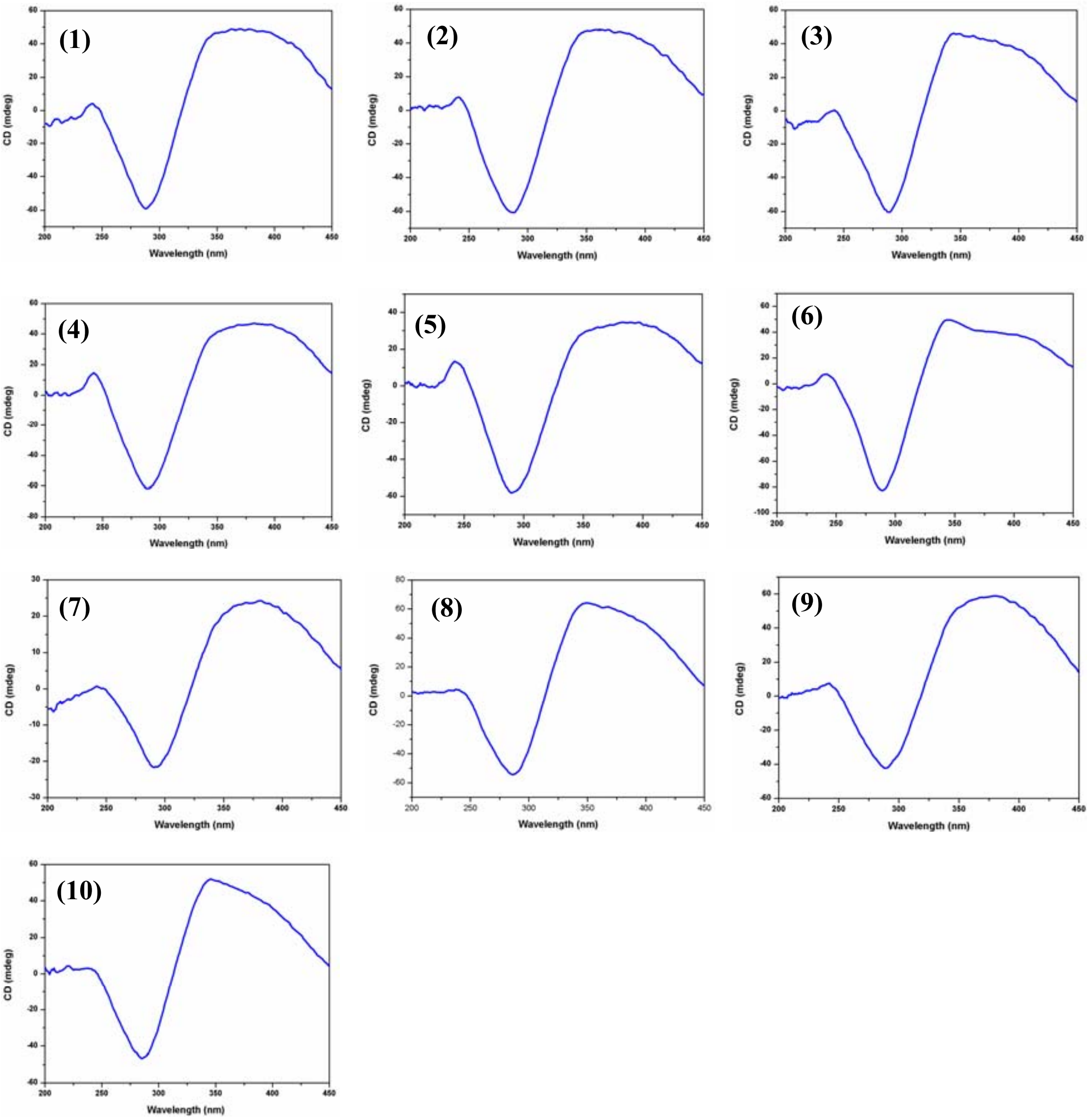

Figure S24. Solid state CD spectra for 10 bulk samples of **1-*M*** from ten independent crystallization batches (induced by ***L*-BPAM**).

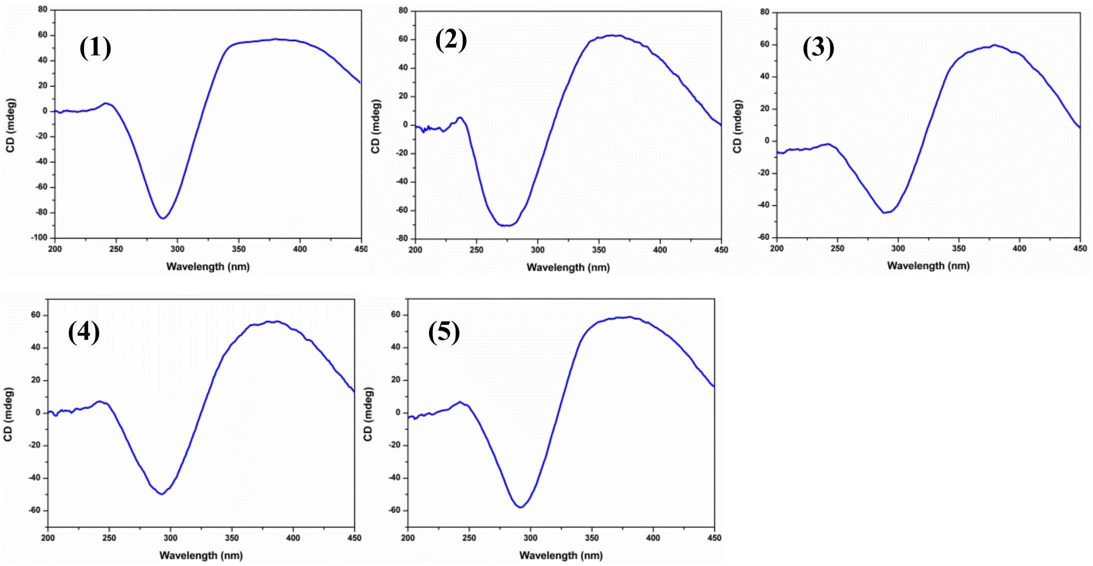

Figure S25. Solid state CD spectra of **1-*M*** used to catalyze the cycloaddition reactions between chiral **BPAM** and methyl acrylate (the crystalline solids of **1-*M*** was collected from five isolated crystallization batches).

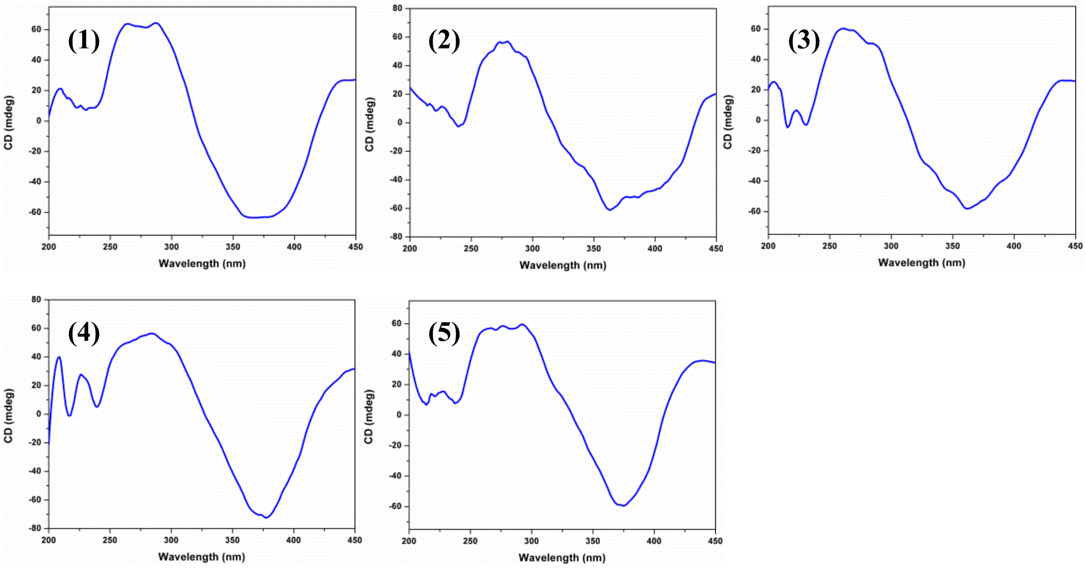

Figure S26. Solid state CD spectra of **1-*P*** used to catalyze the cycloaddition reactions between chiral **BPAM** and methyl acrylate (the crystalline solids of **1-*P*** was collected from five isolated crystallization batches).

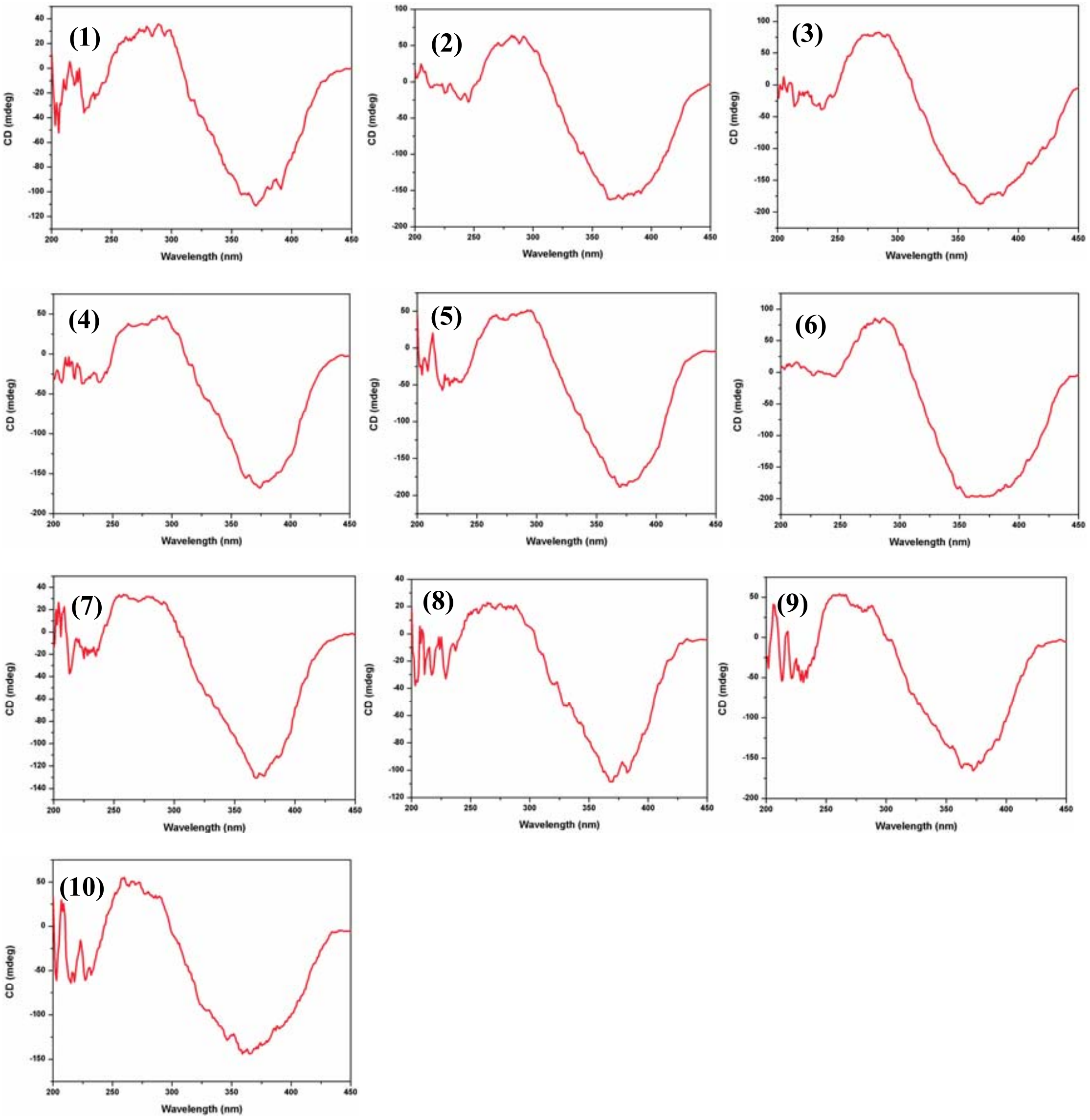

Figure S27. Solid state CD spectra for 10 bulk samples of **1-*P*** from ten independent crystallization batches (induced by ***D*-BPAM**).

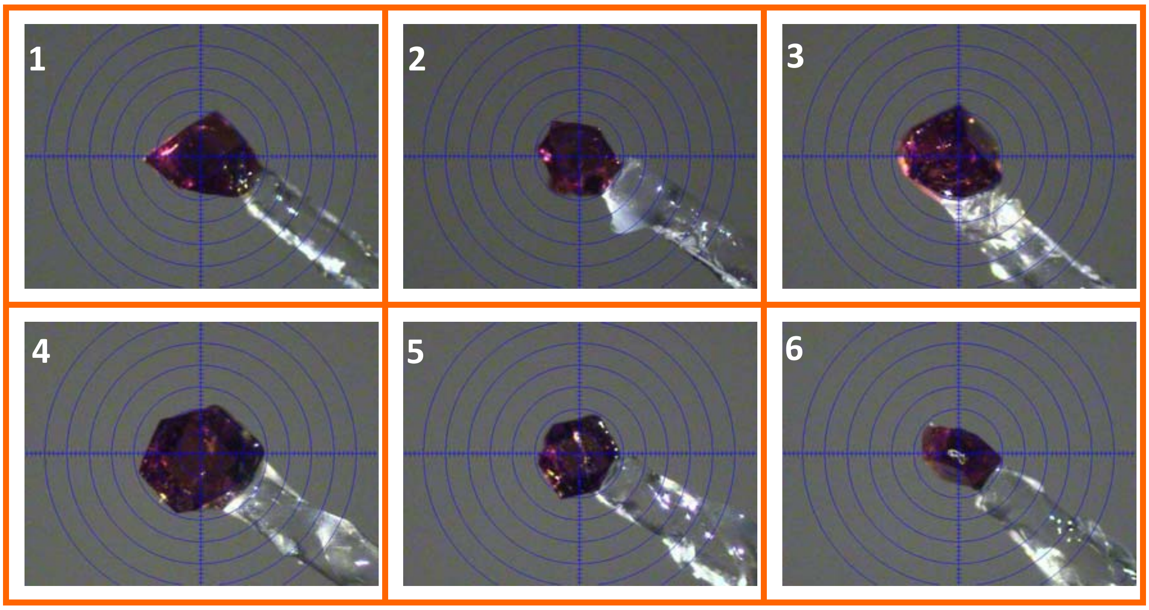

Figure S28. Photographs of six randomly selected crystals for **1-*P*** grown in the presence of ***D*-BPAM** and used for X-ray data collection.

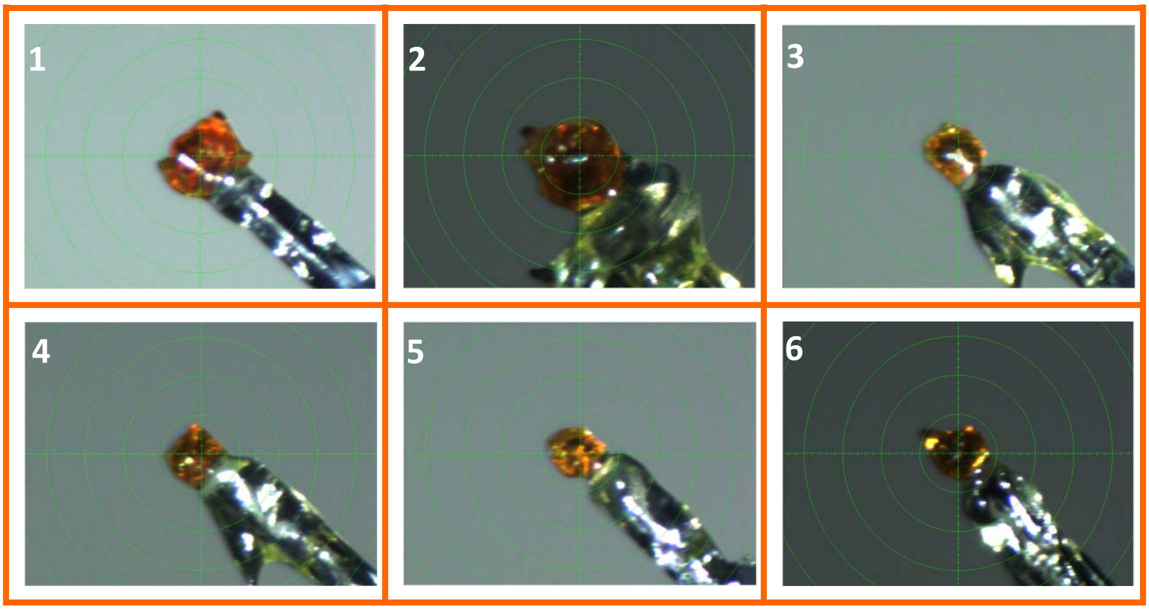

Figure S29. Photographs of six randomly selected crystals for **2-*M*** grown in the presence of ***L*-BPAM** and used for X-ray data collection.

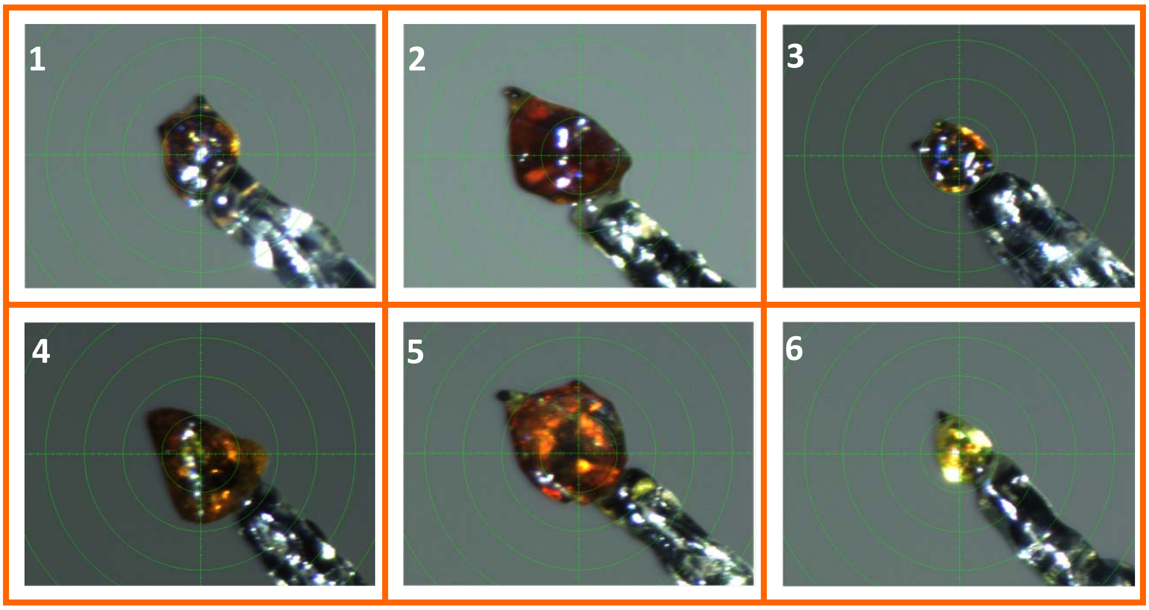

Figure S30. Photographs of six randomly selected crystals for **2-*P*** grown in the presence of ***D*-BPAM** and used for X-ray data collection.

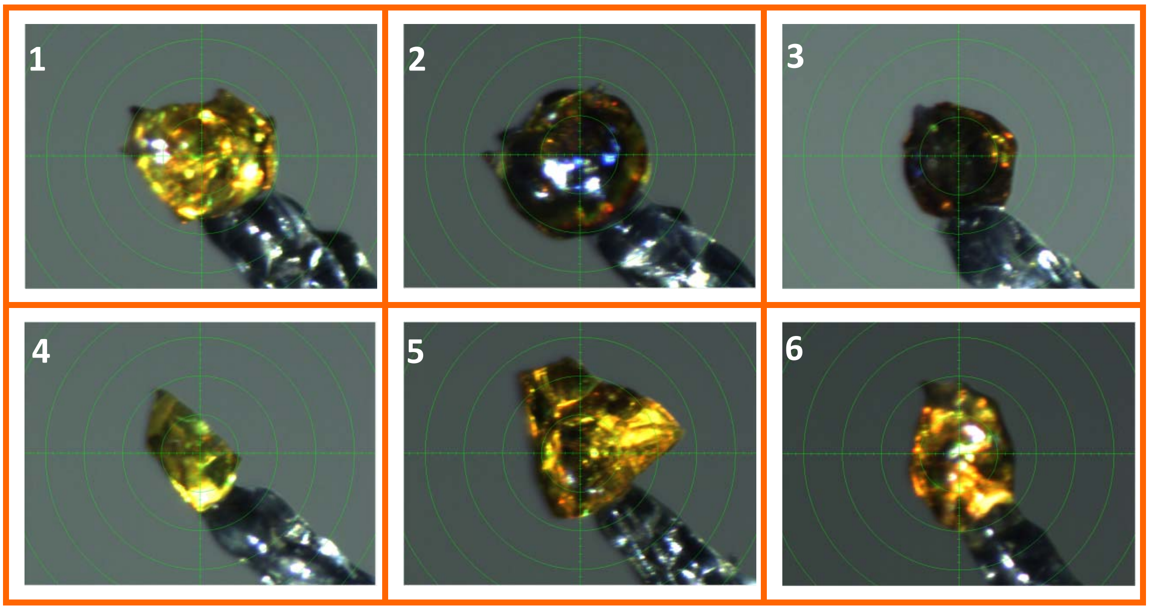

Figure S31. Photographs of six randomly selected crystals for **3-*M*** grown in the presence of ***L*-BPAM** and used for X-ray data collection.

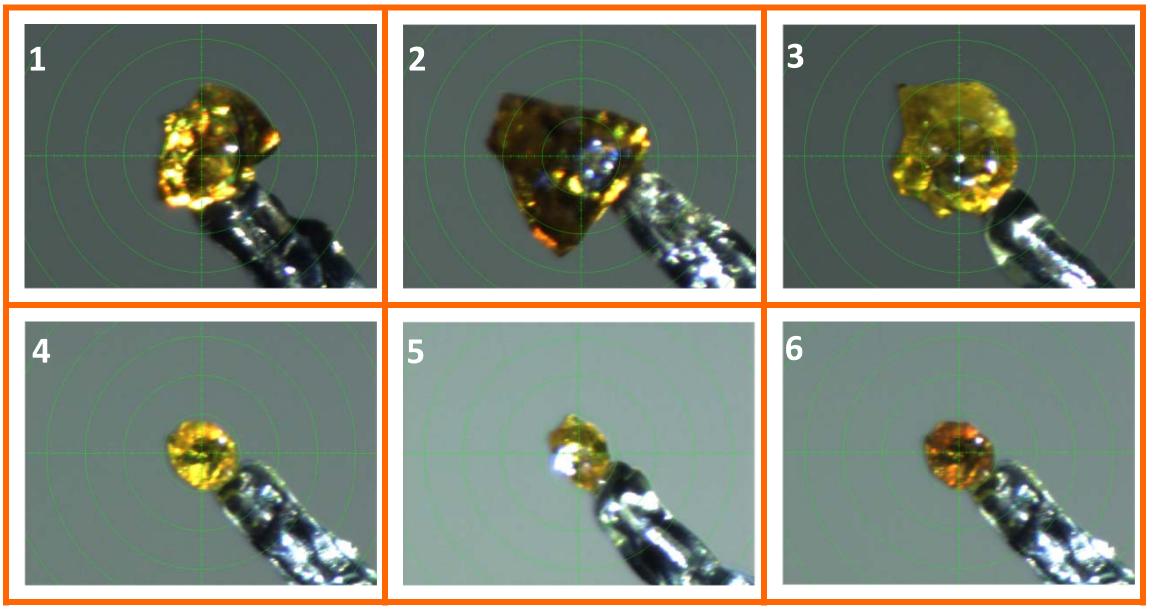

Figure S32. Photographs of six randomly selected crystals for **3-*P*** grown in the presence of ***D*-BPAM** and used for X-ray data collection.

NMR and ESI-MS spectrum

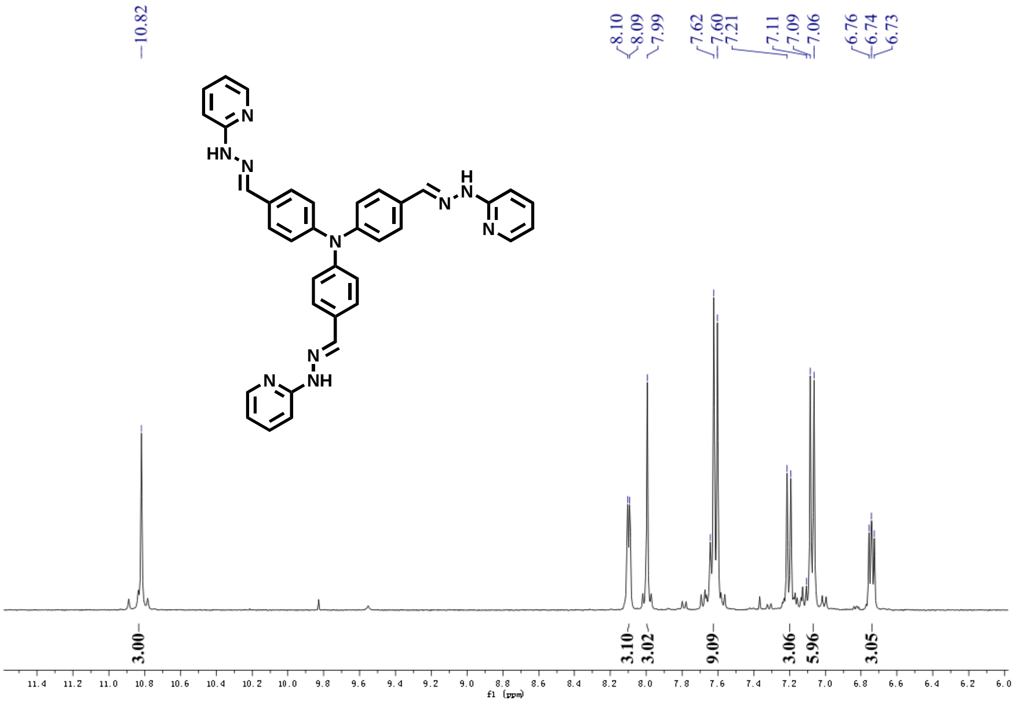

Figure S33. ^{1}H NMR (400 MHz, DMSO-d_6) spectrum of tris(4-(2-pyridin-2-ylhydrazono)phenyl) amine

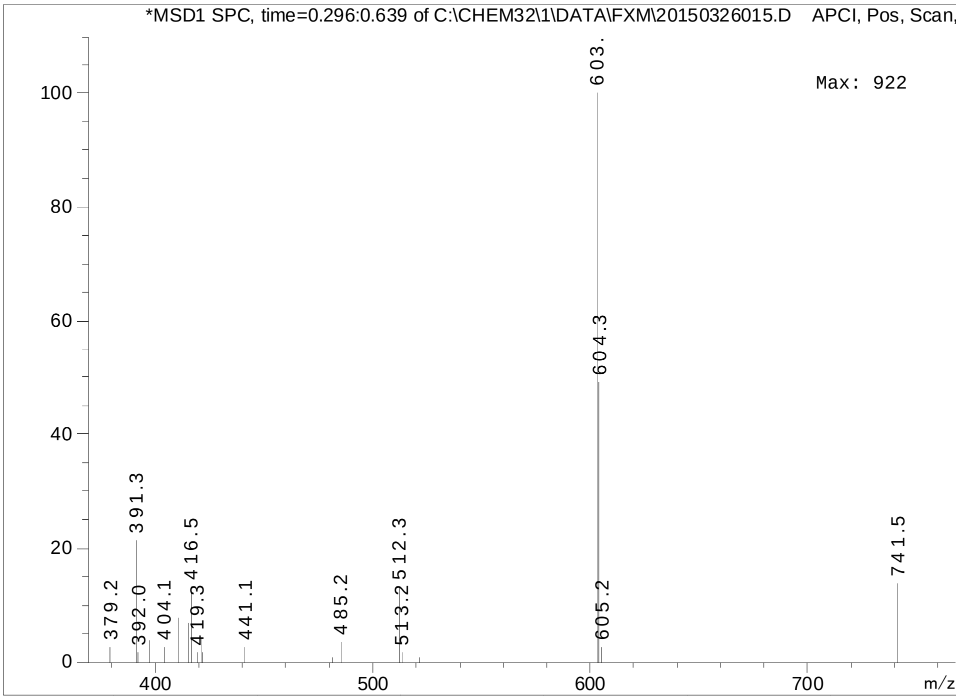

Figure S34. ESI-MS of tris(4-(2-pyridin-2-ylhydrazono)phenyl)amine

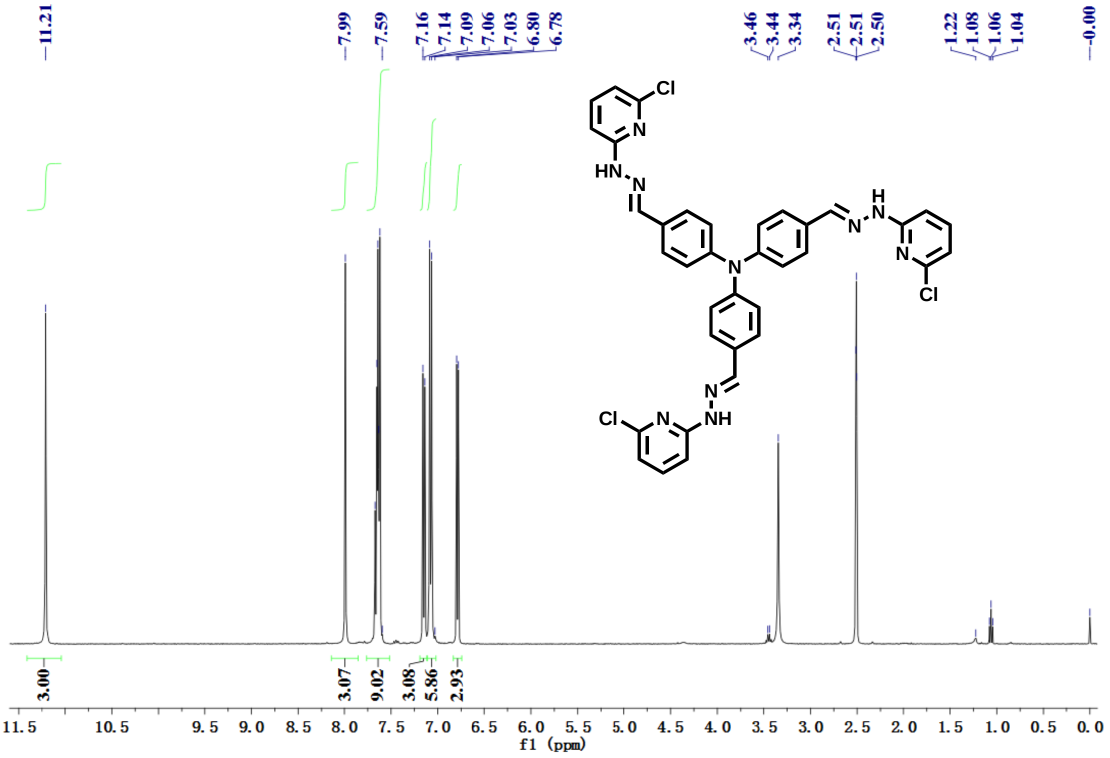

Figure S35. ^{1}H NMR (400 MHz, DMSO-d_6) of tris(4-(6-chloro-2-pyridin-2-ylhydrazono)phenyl) amine

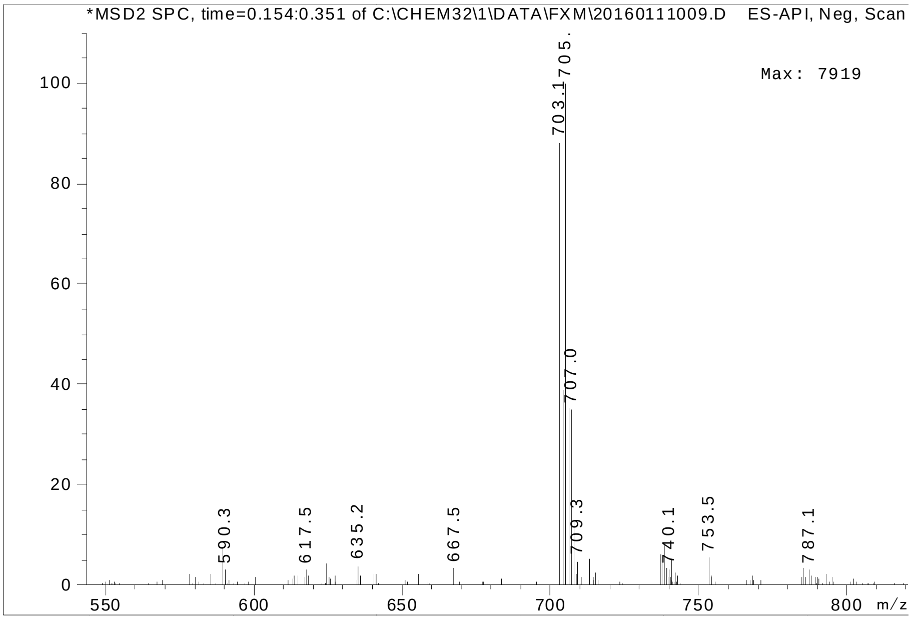

Figure S36. ESI-MS of tris(4-(6-chloro-2-pyridin-2-ylhydrazono)phenyl)amine

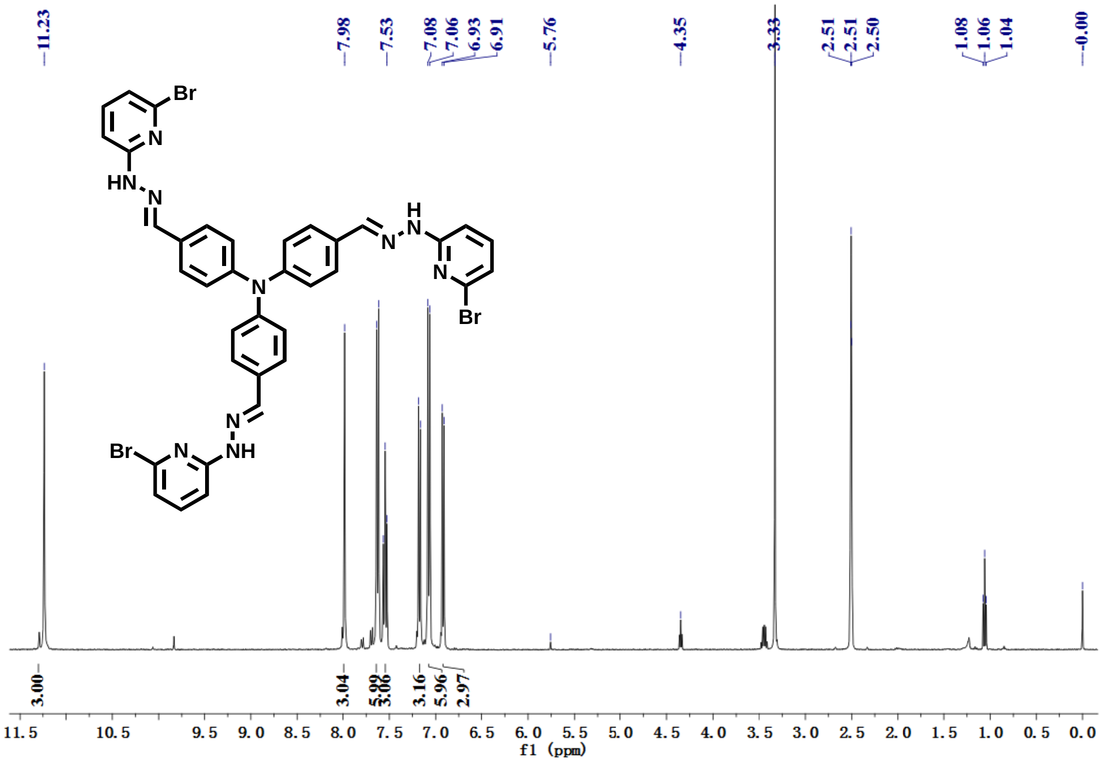

Figure S37. ^{1}H NMR (400 MHz, DMSO-d_6) of tris(4-(6-bromo-2-pyridin-2-ylhydrazono)phenyl) amine

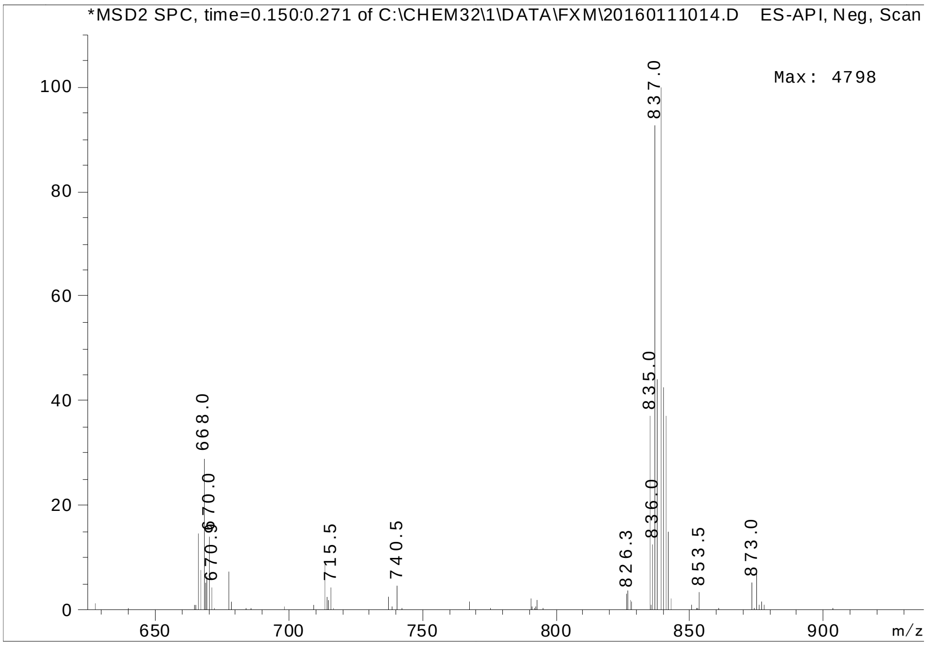

Figure S38. ESI-MS of tris(4-(6-bromo-2-pyridin-2-ylhydrazono)phenyl)amine

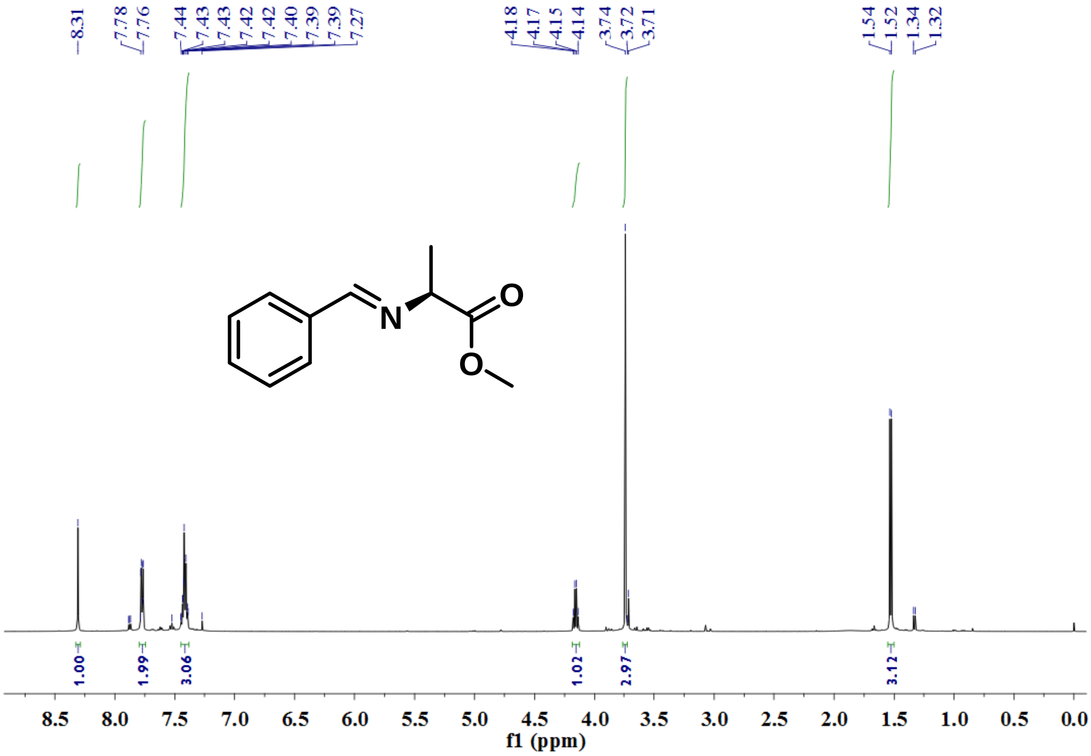

Figure S39. ^{1}H NMR (400 MHz, $CDCl_3$) spectrum of (*S*,*E*)-methyl 2-(benzylideneamino) propanoate

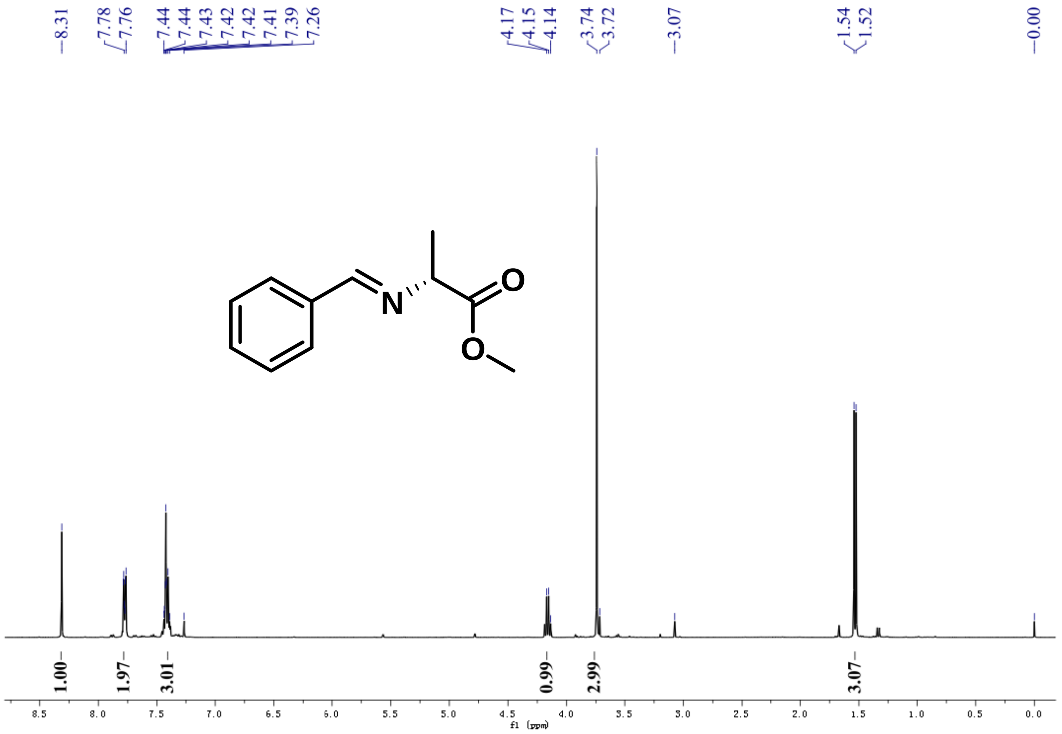

Figure S40. ^{1}H NMR (400 MHz, $CDCl_3$) spectrum of (*R*,*E*)-methyl 2-(benzylideneamino) propanoate

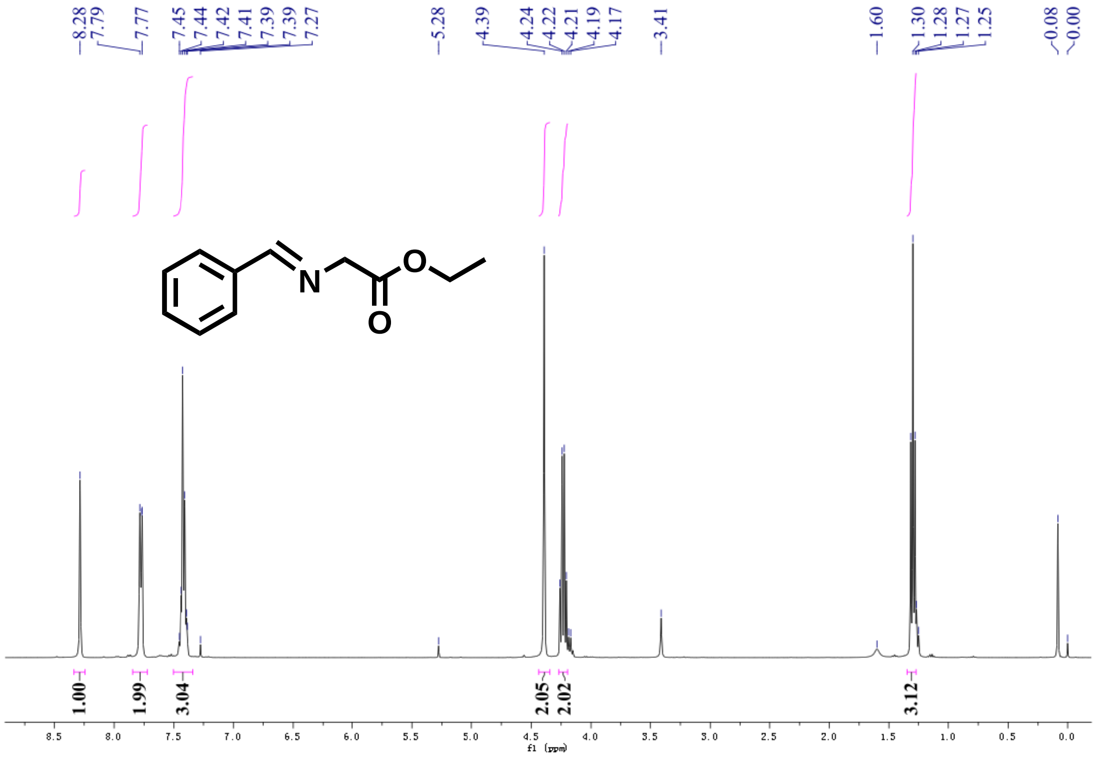

Figure S41. ^{1}H NMR (400 MHz, $CDCl_3$) spectrum of N-(Phenylmethylene)-glycine ethyl ester

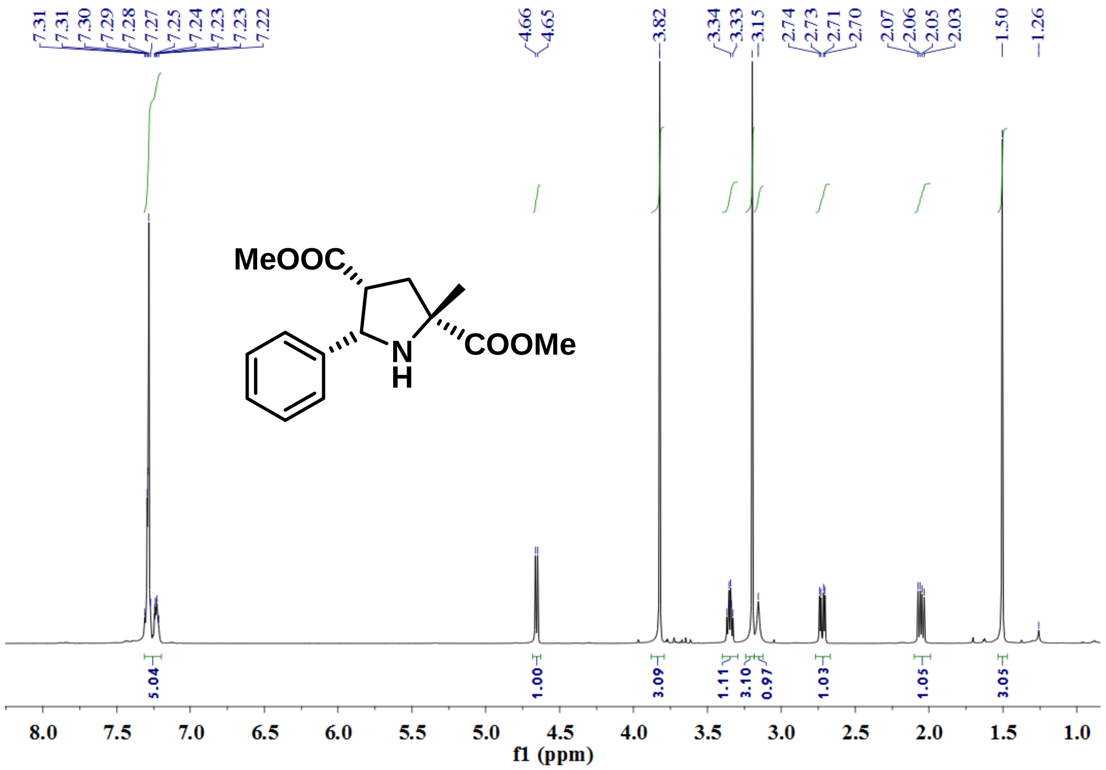

Figure S42. ^{1}H NMR (500 MHz, $CDCl_3$) spectrum of (2*R*,4*R*,5*S*)-Dimethyl-2-methyl-5-phenylpyrrolidine-2,4-dicarboxylate

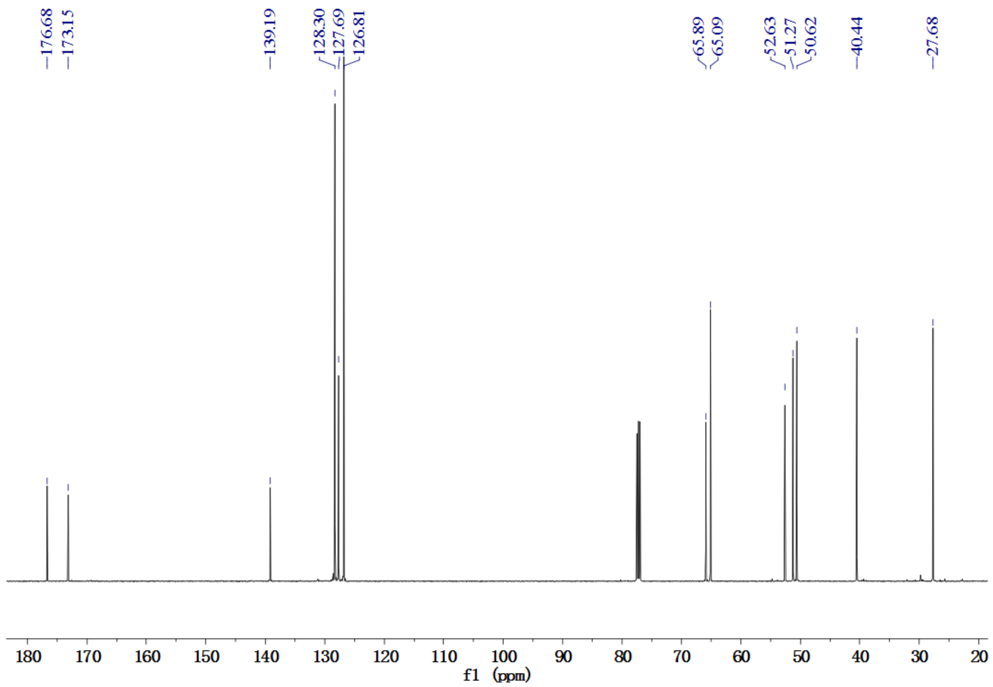

Figure S43. ^{13}C NMR (126 MHz, $CDCl_3$) spectrum of (2*R*,4*R*,5*S*)-Dimethyl-2-methyl-5-phenylpyrrolidine-2,4-dicarboxylate

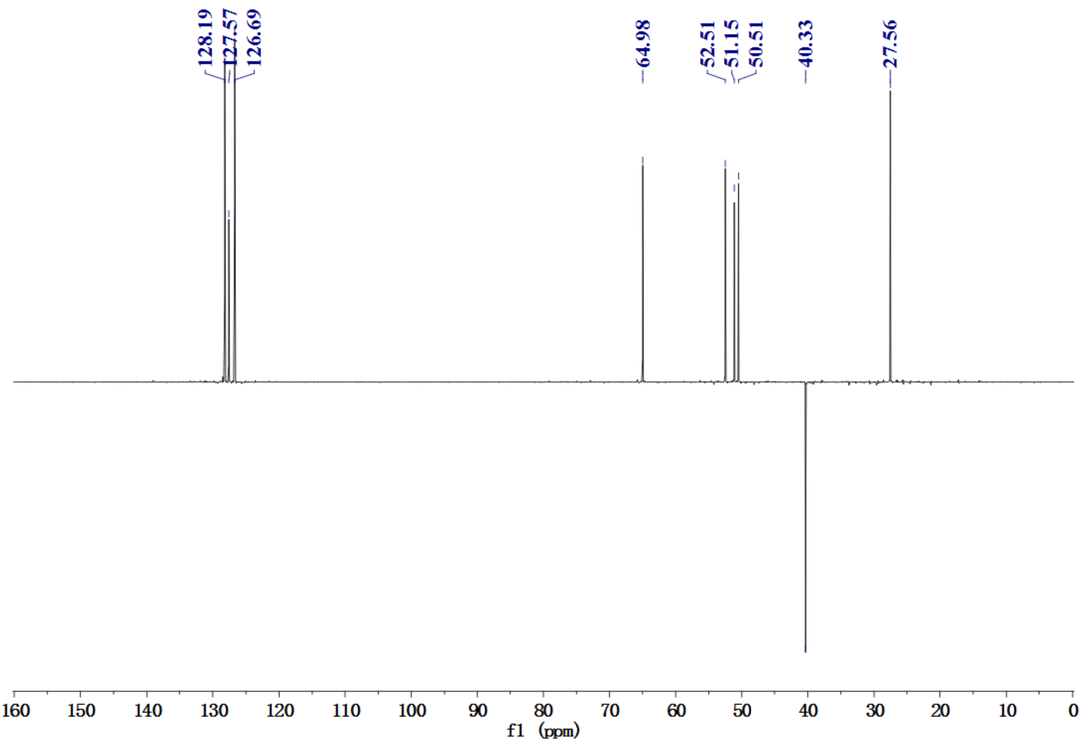

Figure S44. DEPT-135 spectrum of (2*R*,4*R*,5*S*)-Dimethyl-2-methyl-5-phenylpyrrolidine-2,4-dicarboxylate

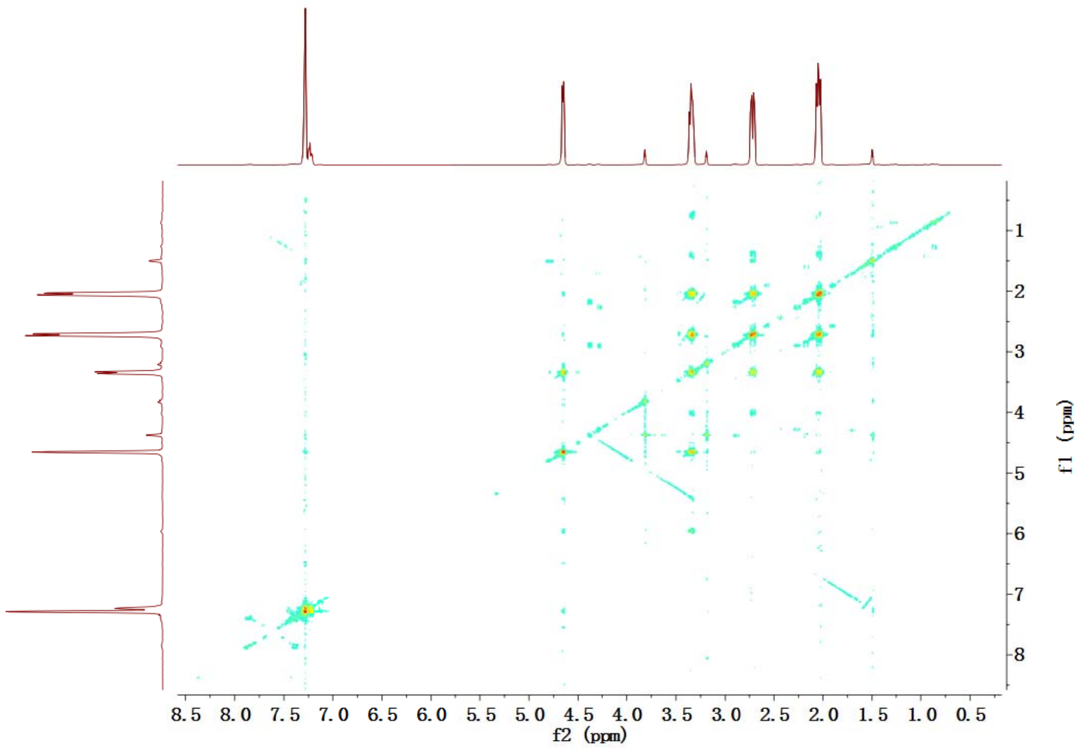

Figure S45. 2D-COSY spectrum of (2*R*,4*R*,5*S*)-Dimethyl-2-methyl-5-phenylpyrrolidine-2,4-dicarboxylate

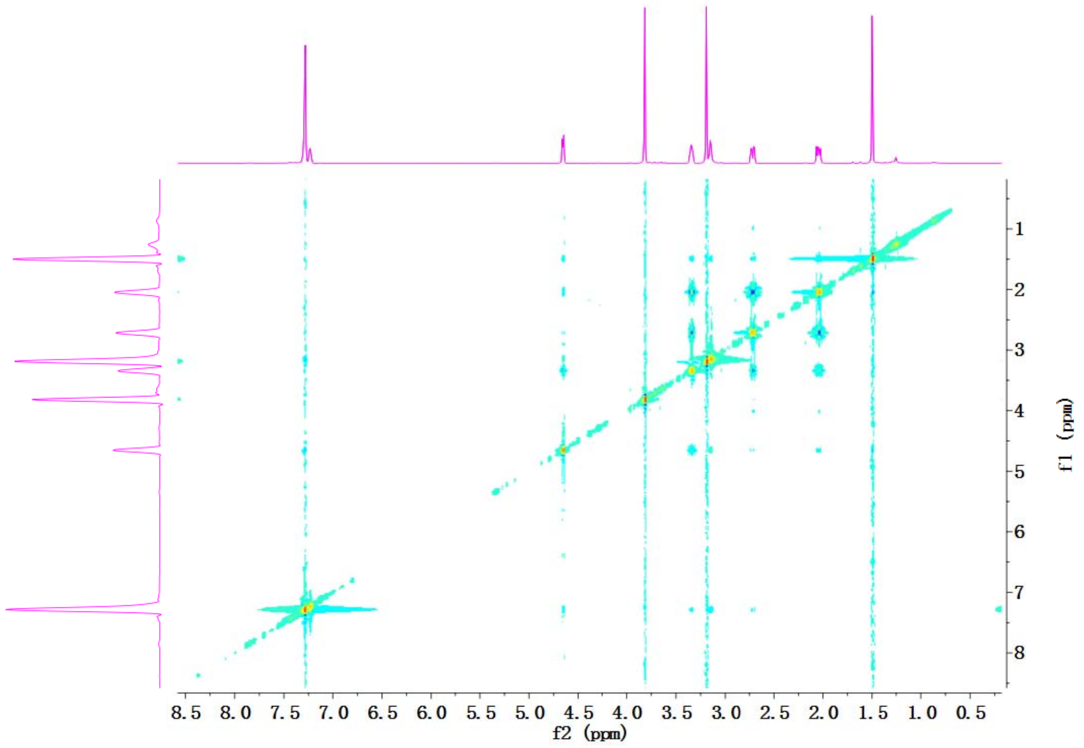

Figure S46. 2D-NOESY spectrum of (2*R*,4*R*,5*S*)-Dimethyl-2-methyl-5-phenylpyrrolidine-2,4-dicarboxylate

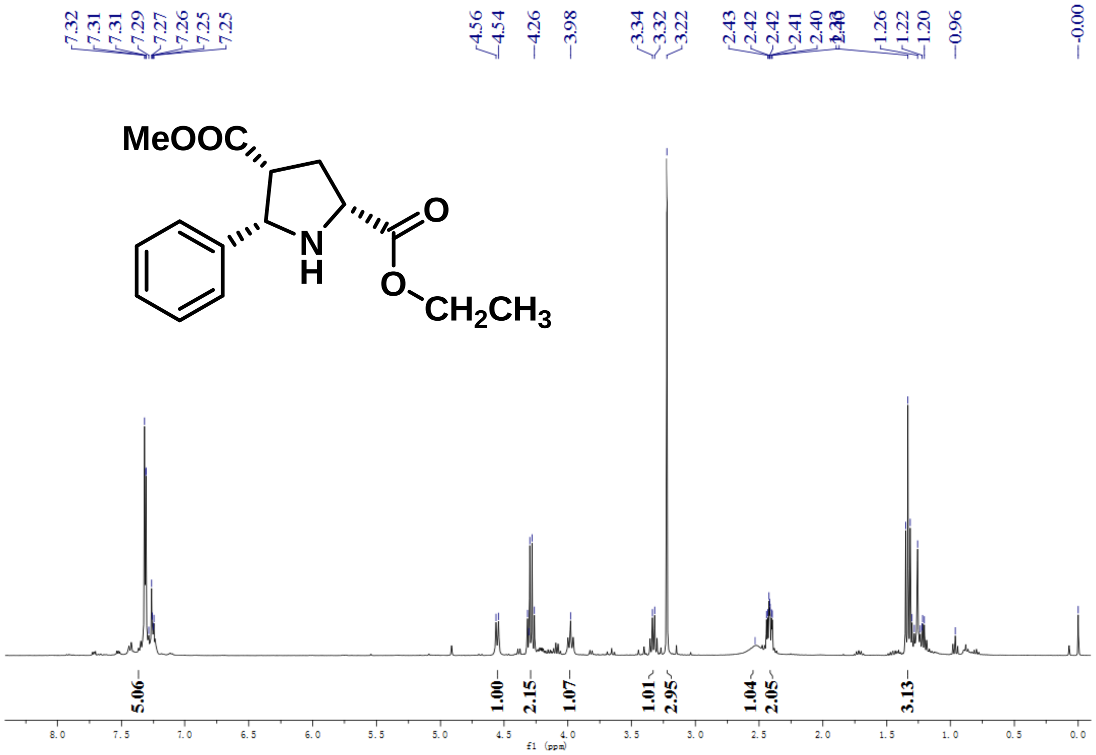

Figure S47. ^{1}H NMR (500 MHz, $CDCl_3$) spectrum of (2*R*,4*R*,5*S*)-4-methylester-2-ethyl-5-phenylpyrrolidine -2,4-dicarboxylate

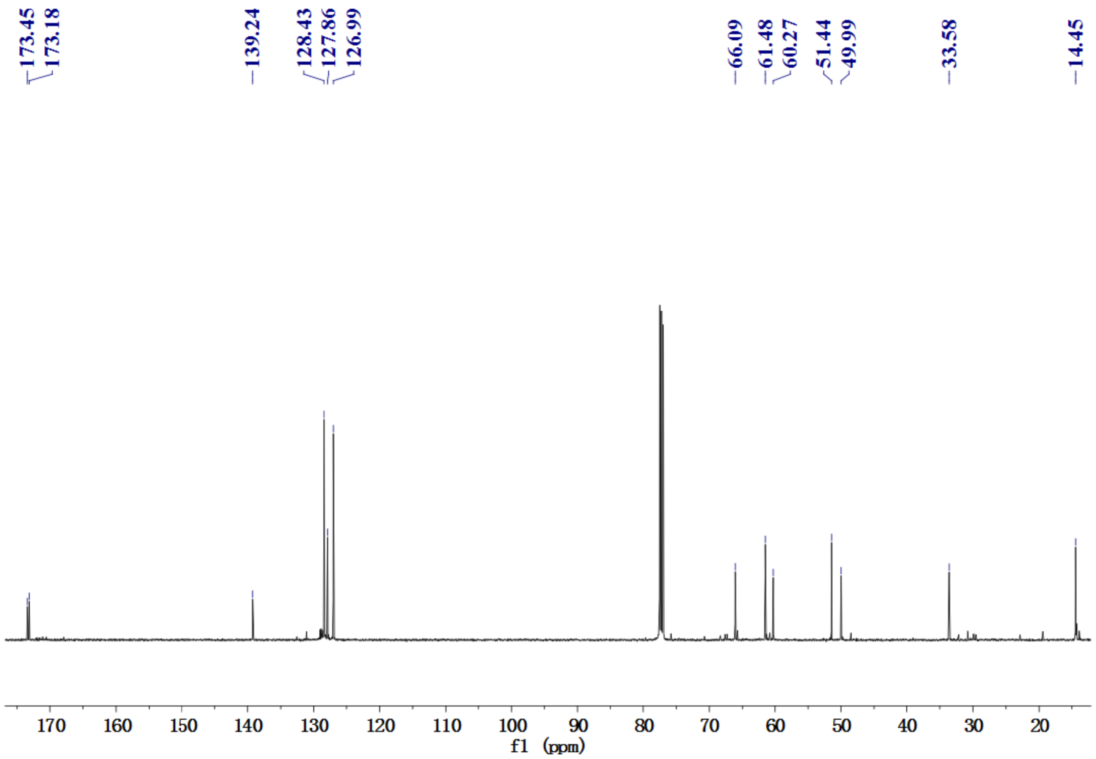

Figure S48. ^{13}C NMR (126 MHz, $CDCl_3$) spectrum of (2*R*,4*R*,5*S*)-4-methylester-2-ethyl-5-phenylpyrrolidine-2,4-dicarboxylate

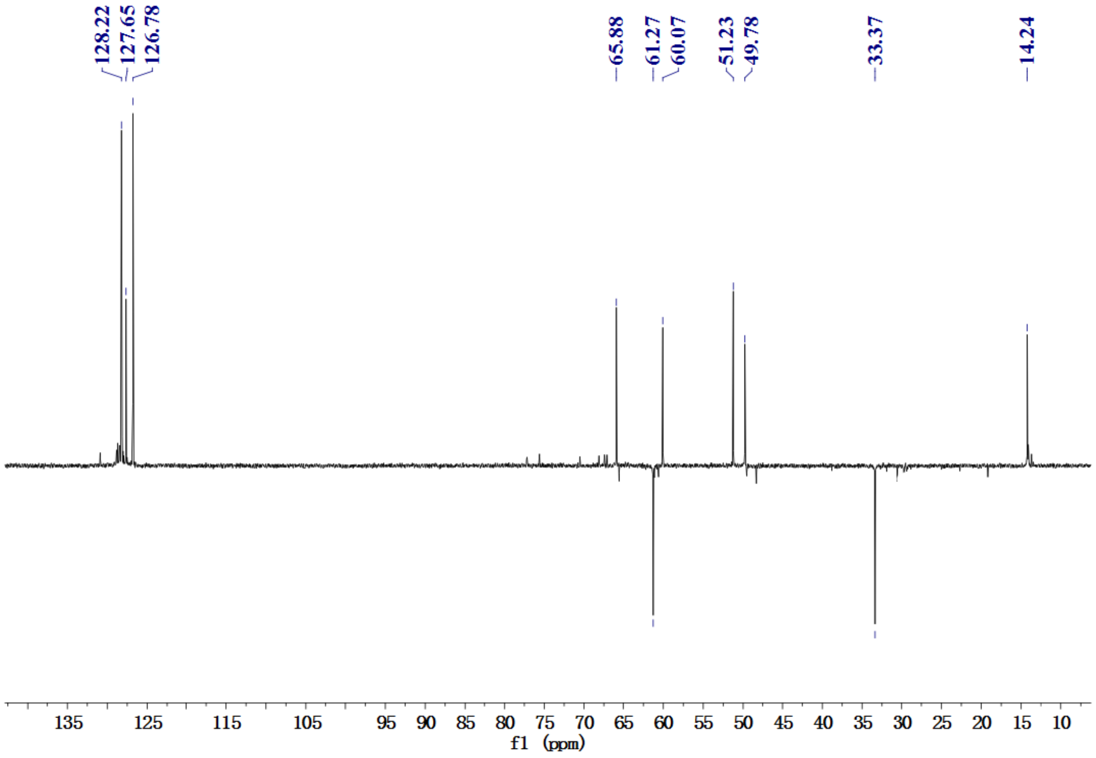

Figure S49. DEPT-135 spectrum of (2*R*,4*R*,5*S*)-4-methylester-2-ethyl-5-phenylpyrrolidine-2,4-dicarboxylate

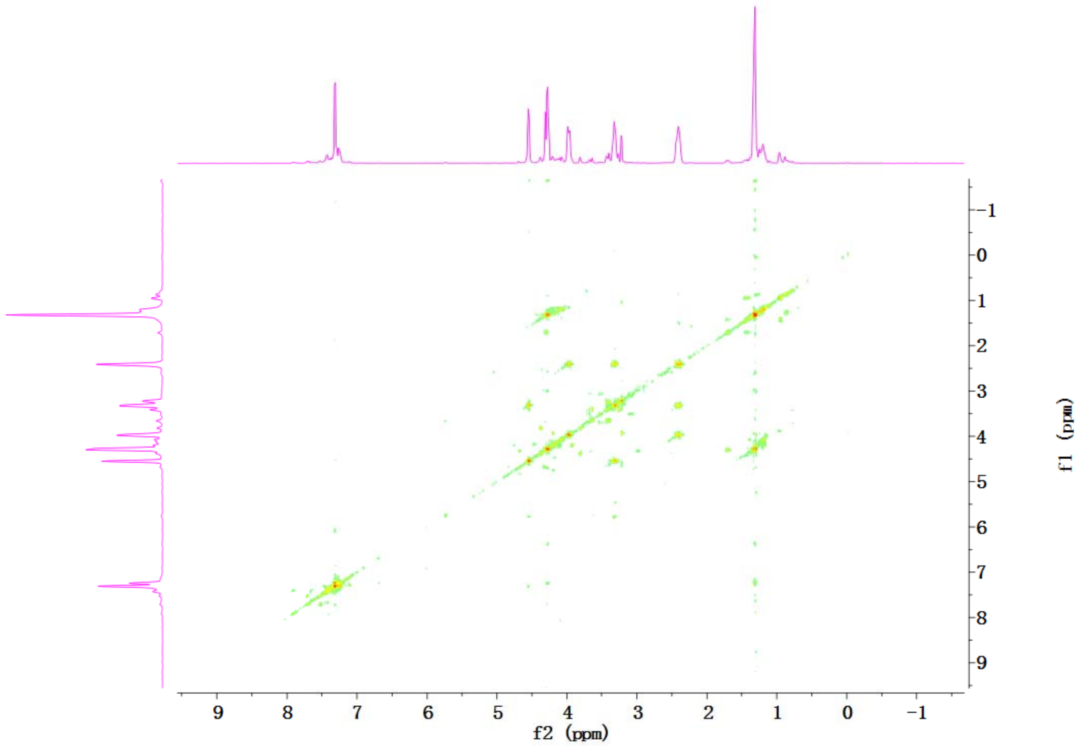

Figure S50. 2D-COSY spectrum of (2*R*,4*R*,5*S*)-4-methylester-2-ethyl-5-phenylpyrrolidine-2,4-dicarboxylate

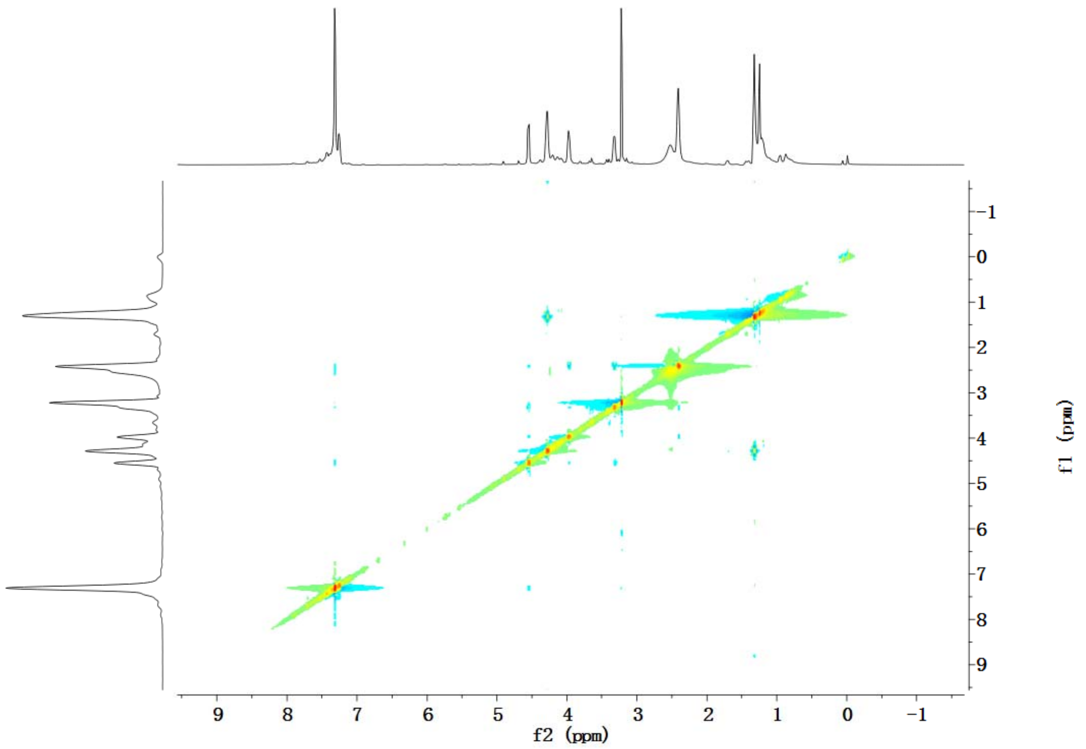

Figure S51. 2D-NOESY spectrum of (2*R*,4*R*,5*S*)-4-methylester-2-ethyl-5-phenylpyrrolidine-2,4-dicarboxylate

HPLC chromatograms

Figure S52. HPLC traces of the products from five parallel cycloaddition reactions between ***L*-BPAM** and methyl acrylate (catalyzed by **1** from five isolated crystallization batches).

(a)

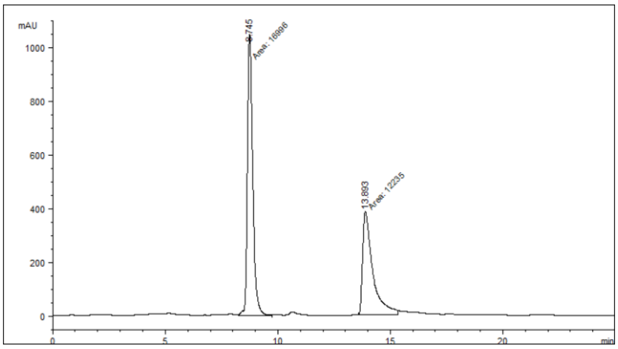

Peak #	RetTime [min]	Area [mAU *s]	Height [mAU]	Area [%]
1	8.745	16996.000	1050.444	58.144
2	13.893	12234.980	385.574	41.856
Total	—	29230.980	1436.018	100.000

(b)

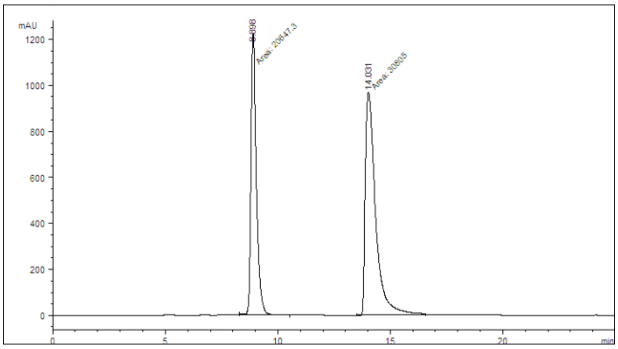

Peak #	RetTime [min]	Area [mAU *s]	Height [mAU]	Area [%]
1	8.898	20647.330	1222.807	40.129
2	14.031	30804.968	970.169	59.871
Total	—	51452.298	2192.976	100.000

(c)

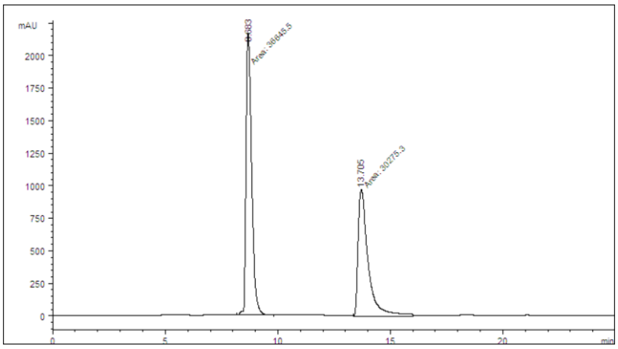

Peak #	RetTime [min]	Area [mAU *s]	Height [mAU]	Area [%]
1	8.683	36645.523	2160.269	54.759

2	13.705	30275.289	973.463	45.241
Total	—	66920.812	3133.732	100.000

(d)

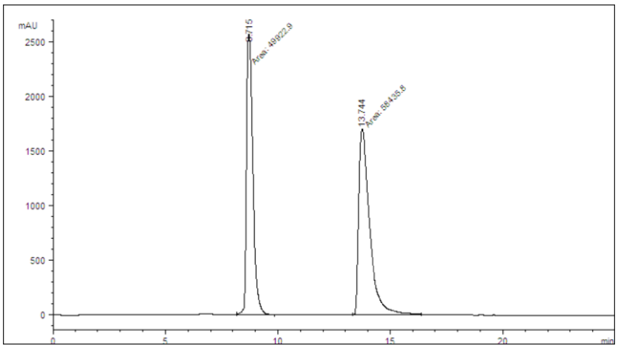

Peak #	RetTime [min]	Area [mAU *s]	Height [mAU]	Area [%]
1	8.715	49922.902	2573.321	46.072
2	13.744	58435.765	1700.359	53.928
Total	—	108358.667	4273.68	100.000

(e)

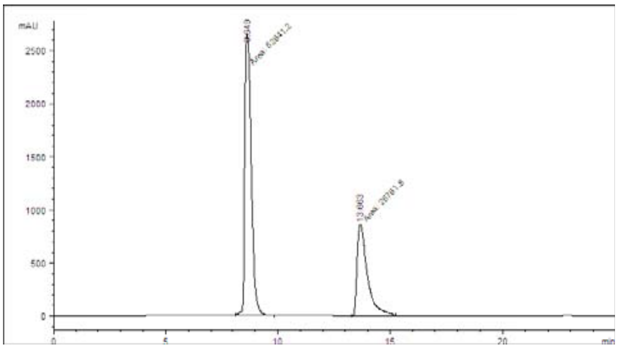

Peak #	RetTime [min]	Area [mAU *s]	Height [mAU]	Area [%]
1	8.649	52641.152	2650.247	66.296
2	13.663	26761.754	868.767	33.704
Total	—	79402.906	3519.014	100.000

Figure S53. HPLC traces of the products from five parallel cycloaddition reactions between ***D*-BPAM** and methyl acrylate (catalyzed by **1** from five isolated crystallization batches).

(a)

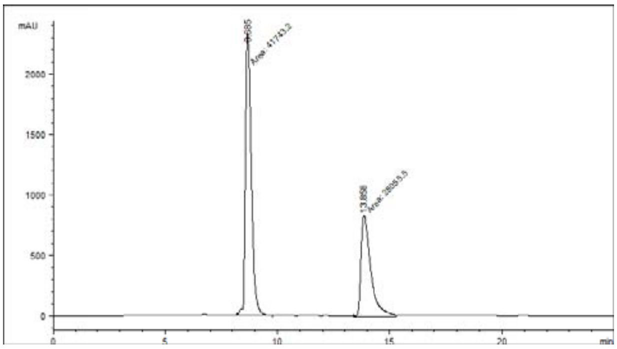

Peak #	RetTime [min]	Area [mAU *s]	Height [mAU]	Area [%]
1	8.685	41743.230	2328.582	59.805
2	13.858	28055.518	865.444	40.195
Total	—	69798.748	3194.026	100.000

(b)

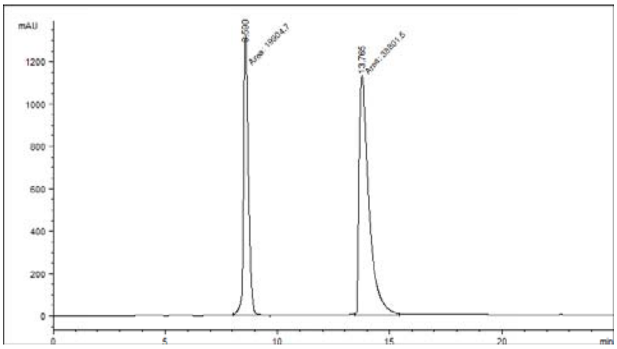

Peak #	RetTime [min]	Area [mAU *s]	Height [mAU]	Area [%]
1	8.590	19904.742	1321.453	33.906
2	13.765	38801.531	1163.686	66.094
Total	—	58706.273	2485.139	100.000

(c)

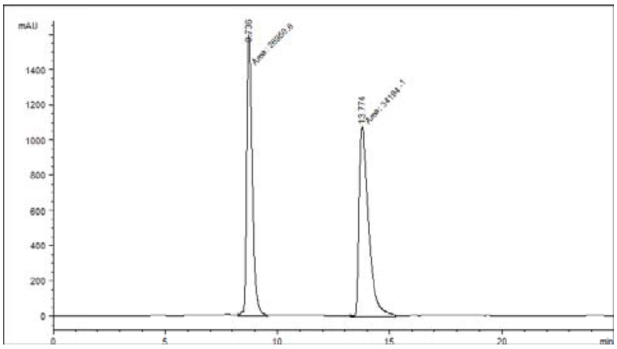

Peak #	RetTime [min]	Area [mAU *s]	Height [mAU]	Area [%]
1	8.736	26950.605	1599.896	44.077
2	13.774	34194.109	1101.019	55.923
Total	—	61144.715	2700.916	100.000

(d)

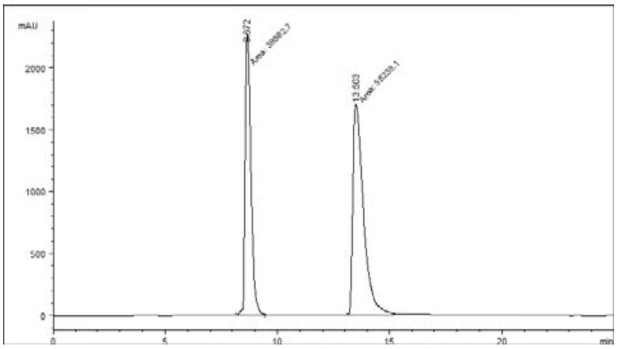

Peak #	RetTime [min]	Area [mAU *s]	Height [mAU]	Area [%]

1	8.672	39592.723	2266.334	40.471
2	13.503	58238.105	1717.554	59.529
Total	—	97830.828	3983.888	100.000

(e)

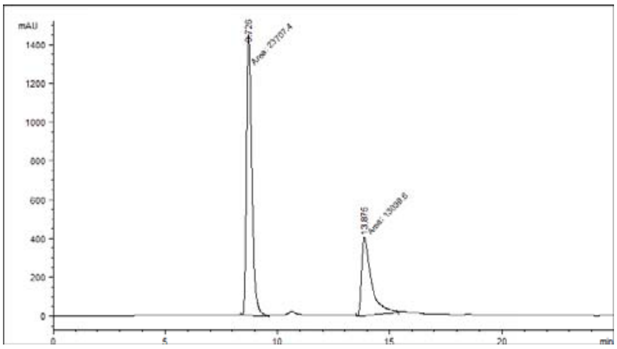

Peak #	RetTime [min]	Area [mAU *s]	Height [mAU]	Area [%]
1	8.726	23707.395	1448.698	64.515
2	13.875	13039.558	408.471	35.485
Total	—	36746.953	1857.169	100.000

Figure S54. HPLC chromatograms of the products in five parallel reactions between **PGE** and methyl acrylate (catalyzed by **1** from five isolated crystallization batches).

(a)

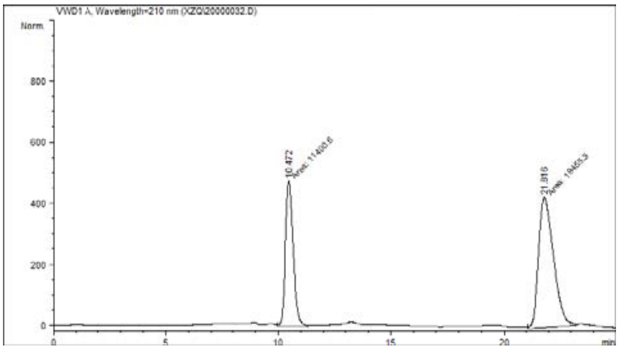

Peak #	RetTime [min]	Area [mAU *s]	Height [mAU]	Area [%]
1	10.472	11400.623	476.316	36.948
2	21.816	19455.295	425.783	63.052
Total	—	30855.918	902.099	100.000

(b)

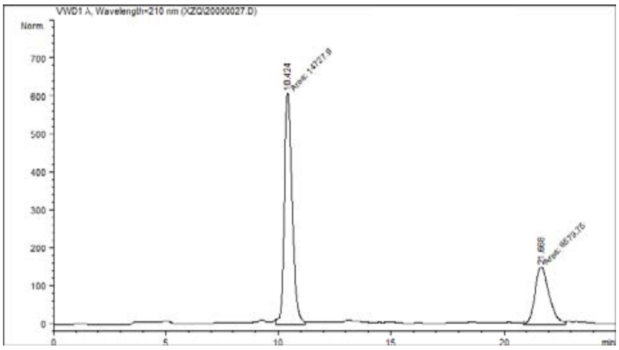

Peak #	RetTime [min]	Area [mAU *s]	Height [mAU]	Area [%]
1	10.424	14727.923	611.393	69.120
2	21.668	6579.746	151.073	30.880
Total	—	21307.669	762.466	100.000

(c)

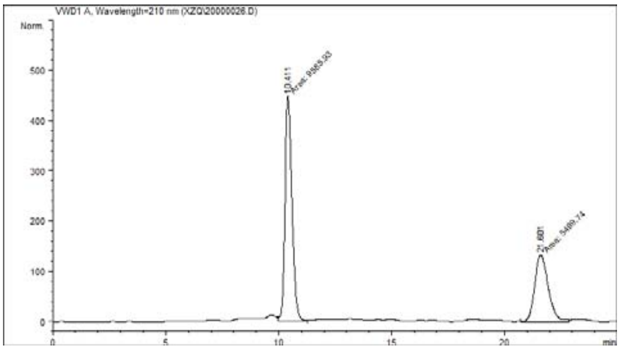

Peak #	RetTime [min]	Area [mAU *s]	Height [mAU]	Area [%]
1	10.411	9585.929	448.796	63.543
2	21.601	5499.740	132.353	36.457
Total	—	15085.669	581.149	100.000

(d)

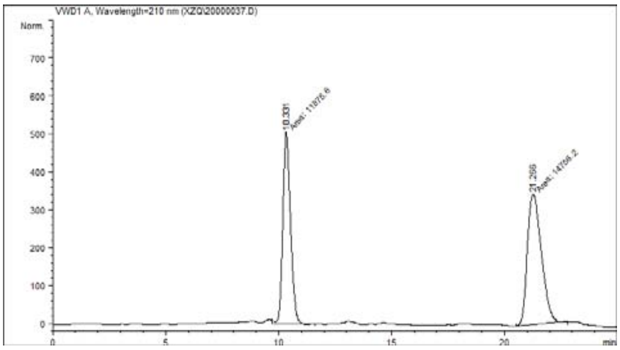

Peak #	RetTime [min]	Area [mAU *s]	Height [mAU]	Area [%]
1	10.331	11875.571	502.935	44.592
2	21.266	14756.199	342.689	55.408
Total	—	26631.770	845.624	100.000

(e)

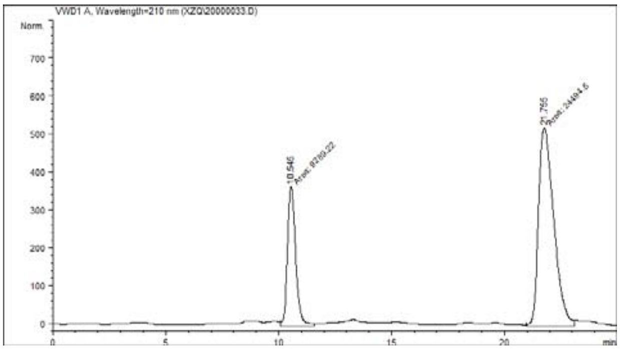

Peak #	RetTime [min]	Area [mAU *s]	Height [mAU]	Area [%]

1	10.545	9289.221	367.765	27.496
2	21.755	24494.549	522.577	72.504
Total	—	33783.770	890.342	100.000

Figure S55. HPLC traces of the products from five parallel cycloaddition reactions between ***L*-BPAM** and methyl acrylate (catalyzed by **1-*M*** from five isolated crystallization batches).

(a)

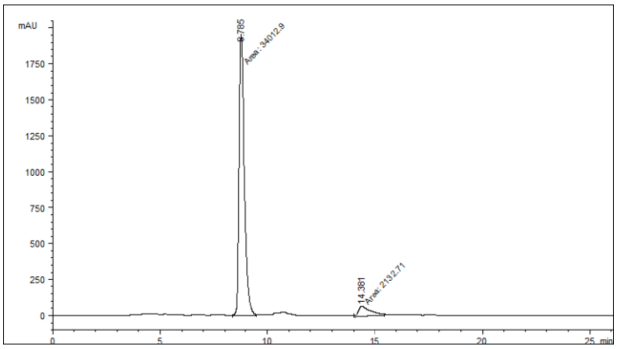

Peak #	RetTime [min]	Area [mAU *s]	Height [mAU]	Area [%]
1	8.785	34012.852	1964.106	94.099
2	14.381	2132.711	64.497	5.901
Total	—	36145.563	2028.603	100.000

(b)

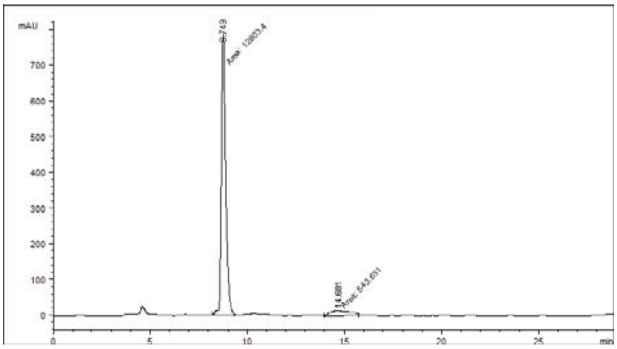

Peak #	RetTime [min]	Area [mAU *s]	Height [mAU]	Area [%]
1	8.749	12603.403	788.802	95.865
2	14.681	543.651	16.364	4.135
Total	—	13147.054	805.166	100.000

(c)

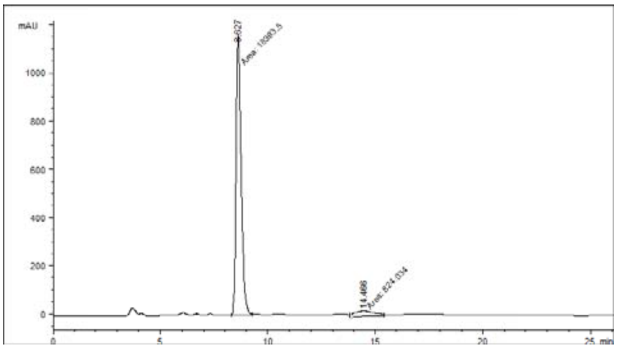

Peak #	RetTime [min]	Area [mAU *s]	Height [mAU]	Area [%]
1	8.627	18393.465	1163.297	95.712
2	14.466	824.034	15.554	4.288
Total	—	19217.499	1178.851	100.000

(d)

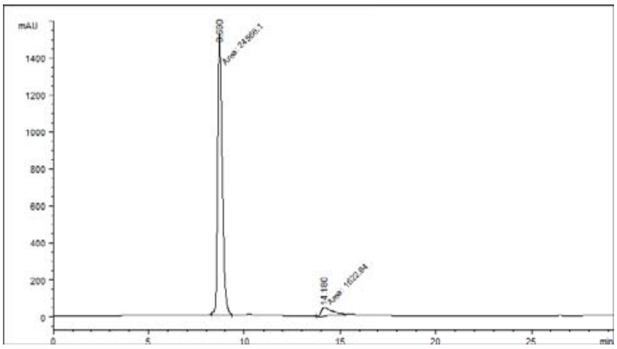

Peak #	RetTime [min]	Area [mAU *s]	Height [mAU]	Area [%]
1	8.690	24566.127	1519.472	93.803
2	14.180	1622.936	46.543	6.197
Total	—	26189.063	1566.015	100.000

(e)

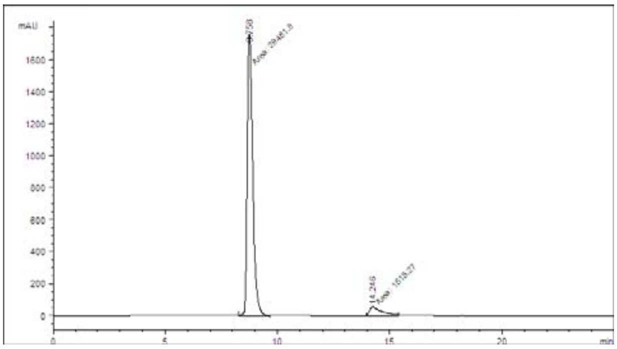

Peak #	RetTime [min]	Area [mAU *s]	Height [mAU]	Area [%]
1	8.758	29481.789	1756.231	95.112
2	14.246	1515.268	54.252	4.888
Total	—	30997.057	1810.484	100.000

Figure S56. HPLC traces of the products from five parallel cycloaddition reactions between ***D*-BPAM** and methyl acrylate (catalyzed by **1-*M*** from five isolated crystallization batches).

(a)

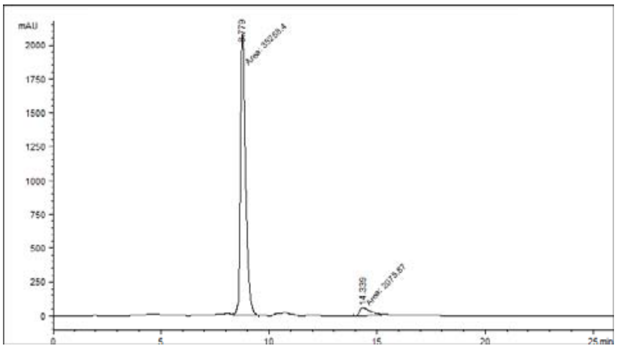

Peak #	RetTime [min]	Area [mAU *s]	Height [mAU]	Area [%]
1	8.779	35258.367	2078.778	94.439
2	14.339	2075.872	60.877	5.561
Total	—	37334.239	2139.655	100.000

(b)

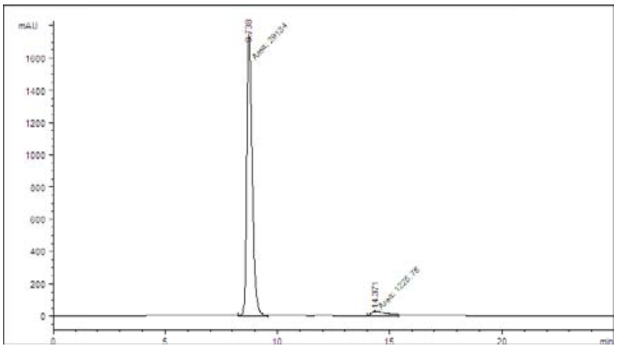

Peak #	RetTime [min]	Area [mAU *s]	Height [mAU]	Area [%]
1	8.738	29134.004	1748.341	95.963
2	14.371	1225.775	26.240	4.037
Total	—	30359.779	1774.581	100.000

(c)

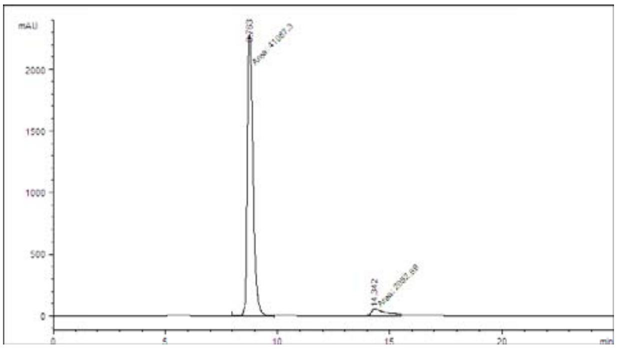

Peak #	RetTime [min]	Area [mAU *s]	Height [mAU]	Area [%]
1	8.763	41087.320	2290.699	95.242
2	14.342	2052.690	55.571	4.758
Total	—	43140.010	2346.27	100.000

(d)

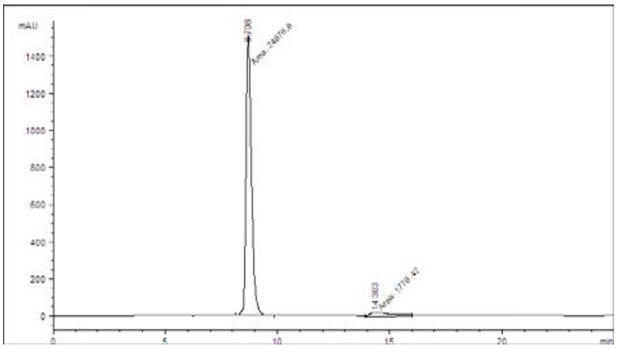

Peak #	RetTime [min]	Area [mAU *s]	Height [mAU]	Area [%]

1	8.708	24676.853	1513.741	93.285
2	14.383	1776.415	26.744	6.715
Total	—	26453.268	1540.485	100.000

(e)

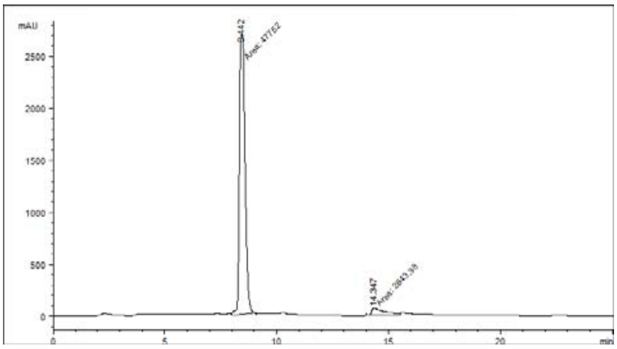

Peak #	RetTime [min]	Area [mAU *s]	Height [mAU]	Area [%]
1	8.442	47752.015	2692.983	94.755
2	14.347	2643.376	69.244	5.245
Total	—	50395.391	2762.227	100.000

Figure S57. HPLC traces of the products from five parallel cycloaddition reactions between ***L*-BPAM** and methyl acrylate (catalyzed by **1-*P*** from five isolated crystallization batches).

(a)

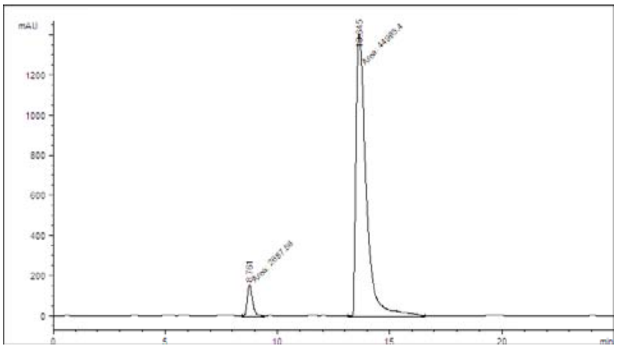

Peak #	RetTime [min]	Area [mAU *s]	Height [mAU]	Area [%]
1	8.761	2687.558	159.155	5.636
2	13.645	44995.410	1408.927	94.364
Total	—	47682.968	1568.082	100.000

(b)

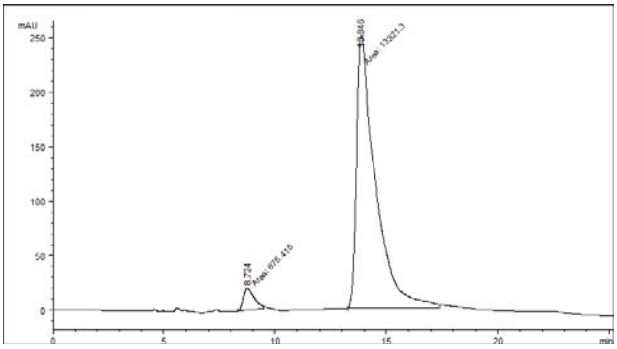

Peak #	RetTime [min]	Area [mAU *s]	Height [mAU]	Area [%]
1	8.724	675.415	20.078	4.826
2	13.846	13321.303	252.114	95.174
Total	—	13996.718	272.192	100.000

(c)

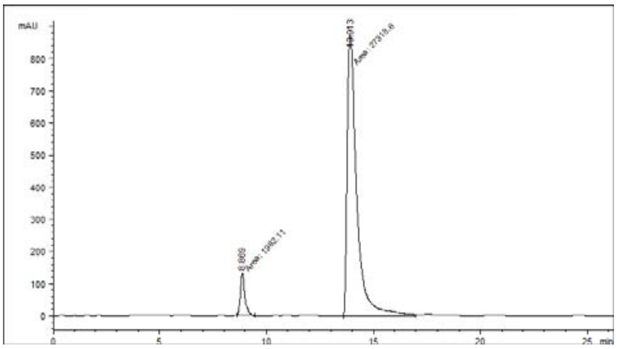

Peak #	RetTime [min]	Area [mAU *s]	Height [mAU]	Area [%]
1	8.869	1962.114	141.163	6.701
2	13.913	27318.576	878.058	93.299
Total	—	29280.69	1019.221	100.000

(d)

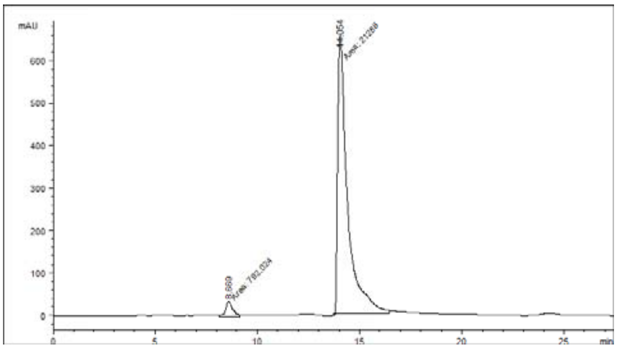

Peak #	RetTime [min]	Area [mAU *s]	Height [mAU]	Area [%]
1	8.669	792.024	26.658	3.587
2	14.054	21286.003	661.888	96.413
Total	—	22078.027	688.546	100.000

(e)

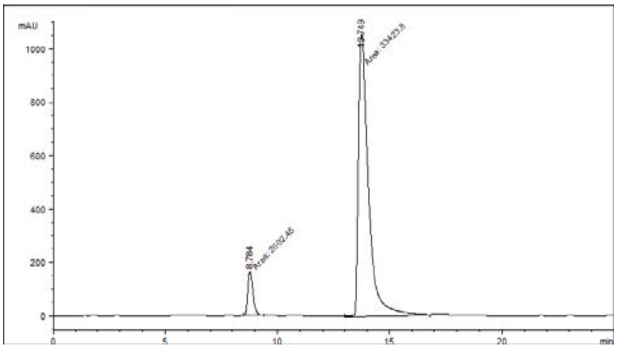

Peak #	RetTime [min]	Area [mAU *s]	Height [mAU]	Area [%]

1	8.784	2002.455	148.024	5.652
2	13.749	33423.836	1058.411	94.348
Total	—	35426.291	1206.436	100.000

Figure S58. HPLC traces of the products from five parallel cycloaddition reactions between ***D*-BPAM** and methyl acrylate (catalyzed by **1-*P*** from five isolated crystallization batches).

(a)

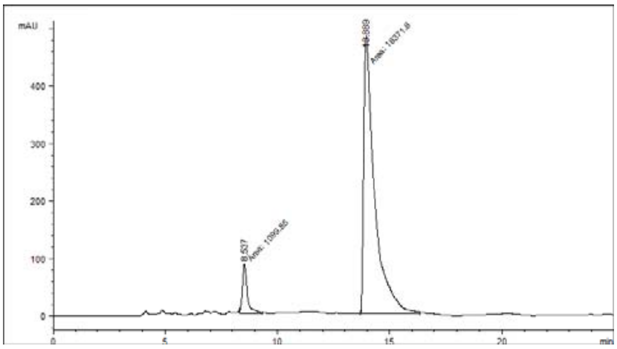

Peak #	RetTime [min]	Area [mAU *s]	Height [mAU]	Area [%]
1	8.537	1099.849	91.711	6.295
2	13.889	16371.593	484.245	93.705
Total	—	17471.442	575.956	100.000

(b)

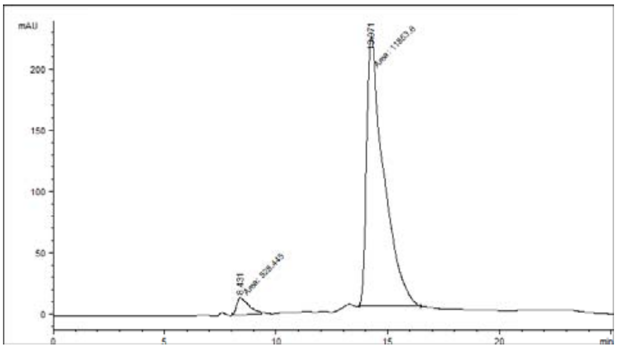

Peak #	RetTime [min]	Area [mAU *s]	Height [mAU]	Area [%]
1	8.431	528.445	17.138	4.268
2	13.971	11853.604	228.809	95.732
Total	—	12382.049	245.947	100.000

(c)

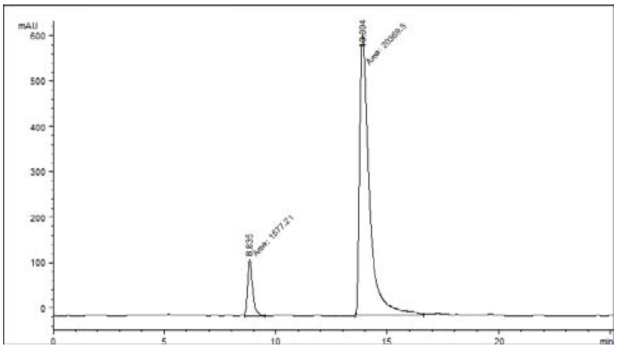

Peak #	RetTime [min]	Area [mAU *s]	Height [mAU]	Area [%]
1	8.835	1577.208	130.618	7.187
2	13.894	20369.451	627.660	92.813
Total	—	21946.659	758.278	100.000

(d)

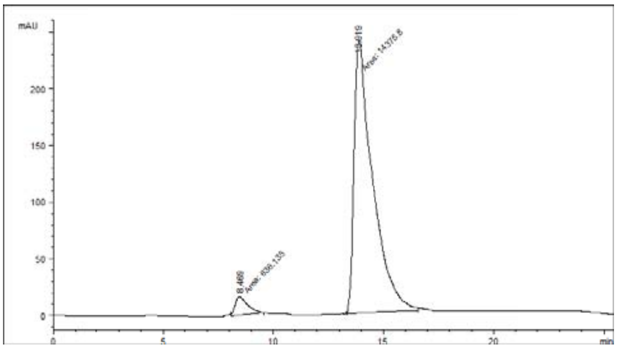

Peak #	RetTime [min]	Area [mAU *s]	Height [mAU]	Area [%]
1	8.469	636.135	18.573	4.238
2	13.919	14375.812	247.200	95.762
Total	—	15011.948	265.773	100.000

(e)

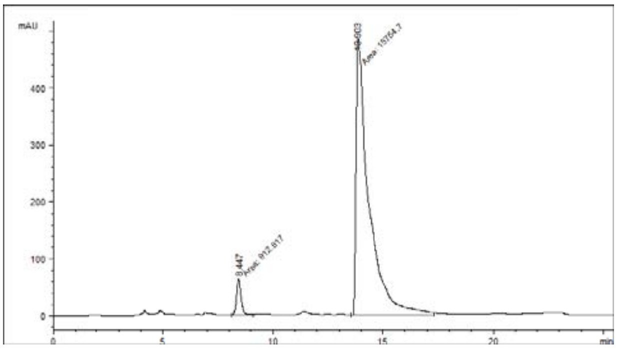

Peak #	RetTime [min]	Area [mAU *s]	Height [mAU]	Area [%]
1	8.447	912.617	61.512	5.475
2	13.903	15754.732	490.313	94.525
Total	—	16667.349	551.825	100.000

Figure S59. HPLC traces of the products from five parallel tandem catalytic systems of **1+*L*-BPAM** (entry 7, Table 1).

(a)

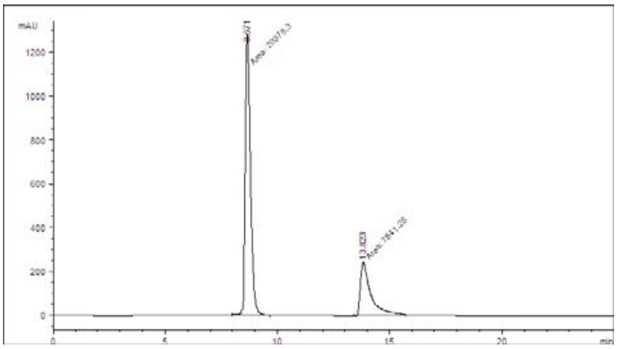

Peak #	RetTime [min]	Area [mAU *s]	Height [mAU]	Area [%]
1	8.671	20378.318	1284.030	72.213
2	13.823	7841.246	243.656	27.787
Total	—	28219.564	1527.686	100.000

(b)

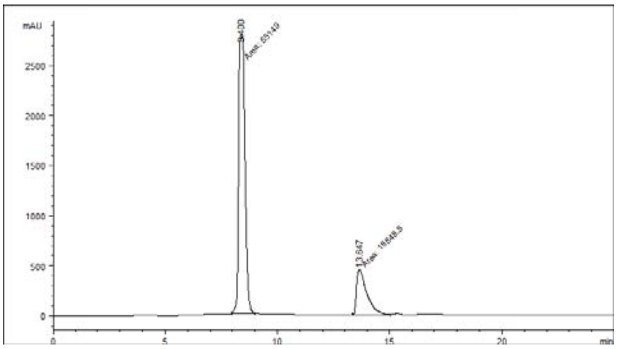

Peak #	RetTime [min]	Area [mAU *s]	Height [mAU]	Area [%]
1	8.400	55148.988	2779.704	74.528
2	13.647	18848.533	500.258	25.472
Total	—	73997.521	3279.962	100.000

(c)

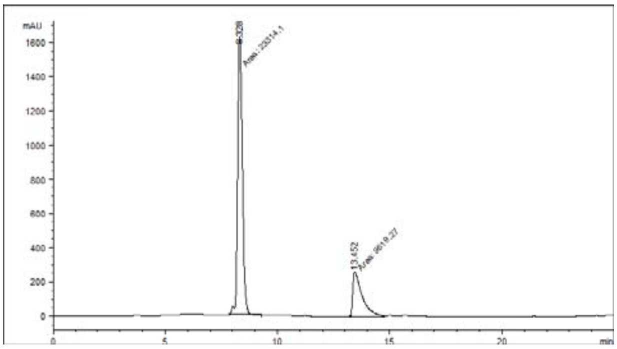

Peak #	RetTime [min]	Area [mAU *s]	Height [mAU]	Area [%]
1	8.328	23314.055	1630.087	70.792
2	13.452	9619.270	280.619	29.208
Total	—	32933.324	1910.706	100.000

(d)

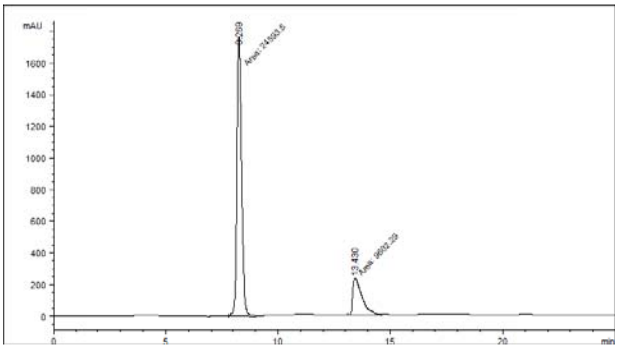

Peak #	RetTime [min]	Area [mAU *s]	Height [mAU]	Area [%]

1	8.269	24593.459	1761.181	71.920
2	13.430	9602.290	269.030	28.080
Total	—	34195.749	2030.211	100.000

(e)

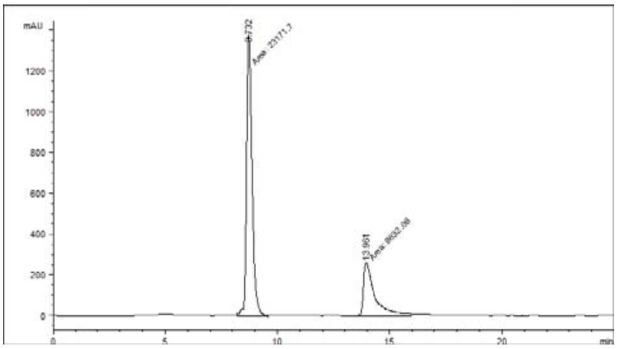

Peak #	RetTime [min]	Area [mAU *s]	Height [mAU]	Area [%]
1	8.732	23171.740	1384.061	70.637
2	13.961	9632.063	263.862	29.363
Total	—	32803.803	1647.923	100.000

Figure S60. HPLC traces of the products from five parallel tandem catalytic systems of **1+*D*-BPAM** (entry 8, Table 1).

(a)

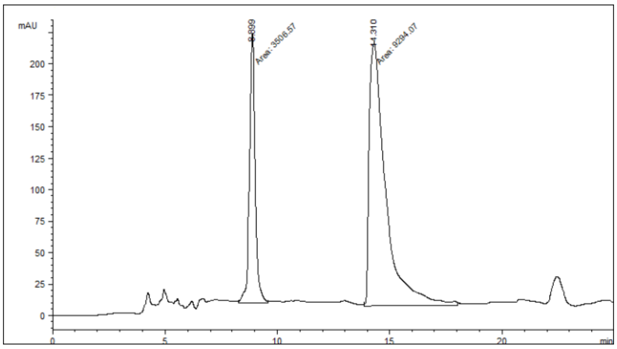

Peak #	RetTime [min]	Area [mAU *s]	Height [mAU]	Area [%]
1	8.899	3506.568	210.240	27.394
2	14.310	9294.073	210.059	72.606
Total	—	12800.642	420.298	100.000

(b)

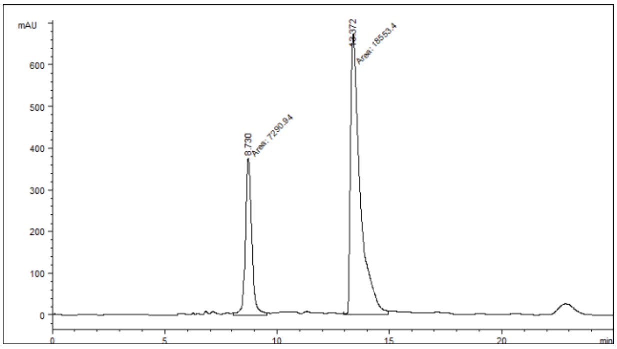

Peak #	RetTime [min]	Area [mAU *s]	Height [mAU]	Area [%]
1	8.730	7290.941	379.183	28.211
2	13.372	18553.389	674.415	71.789
Total	–	25844.330	1053.598	100.000

(c)

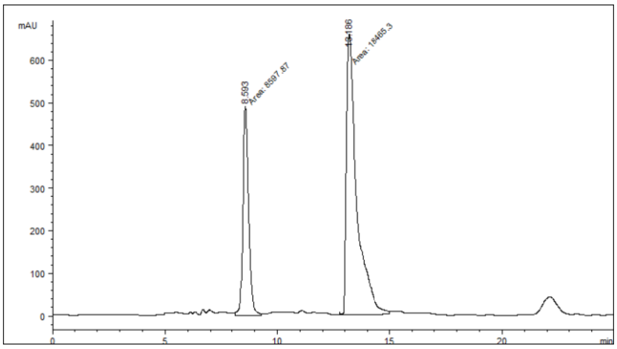

Peak #	RetTime [min]	Area [mAU *s]	Height [mAU]	Area [%]
1	8.593	8597.870	491.639	31.770
2	13.186	18465.318	659.148	68.230
Total	–	27063.188	1150.787	100.000

(d)

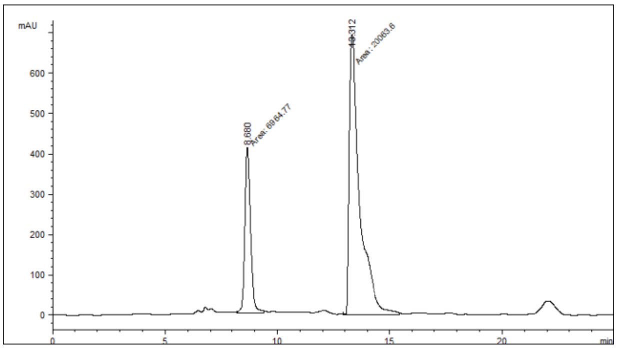

Peak #	RetTime [min]	Area [mAU *s]	Height [mAU]	Area [%]
1	8.680	6964.773	410.698	25.768
2	13.312	20063.639	697.459	74.232
Total	–	27028.412	1108.157	100.000

(e)

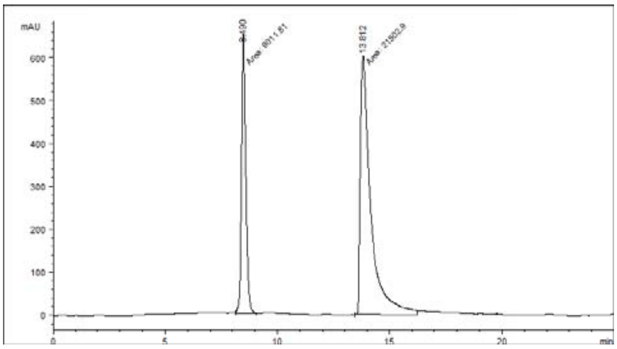

Peak #	RetTime [min]	Area [mAU *s]	Height [mAU]	Area [%]

1	8.490	9011.808	649.603	29.533
2	13.812	21502.949	605.420	70.467
Total	—	30514.757	1255.023	100.000

Figure S61. HPLC trace of the product from the cycloaddition reaction between ***D*-BPAM** and methyl acrylate (catalyzed by **2-*M***).

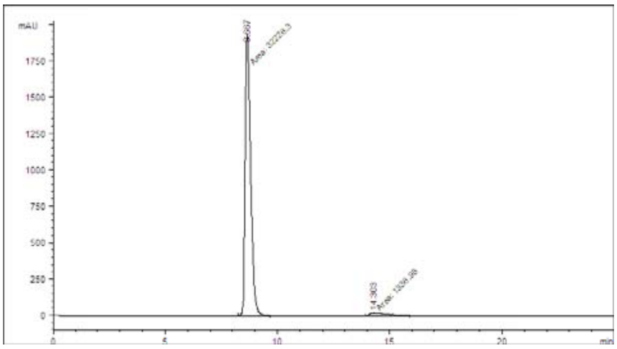

Peak #	RetTime [min]	Area [mAU *s]	Height [mAU]	Area [%]
1	8.667	32226.299	1934.385	96.016
2	14.303	1336.953	23.927	3.984
Total	—	33563.252	1958.312	100.000

Figure S62. HPLC trace of the product from the cycloaddition reaction between ***D*-BPAM** and methyl acrylate (catalyzed by **2-*P***).

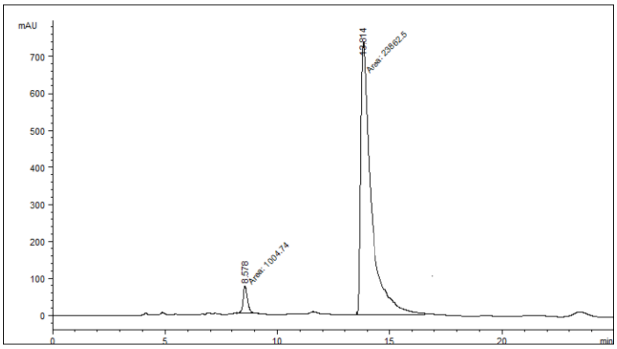

Peak #	RetTime [min]	Area [mAU *s]	Height [mAU]	Area [%]
1	8.578	1004.736	72.762	4.040
2	13.814	23862.504	760.413	95.960
Total	—	24867.240	833.176	100.000

Figure S63. HPLC trace of the product from the cycloaddition reaction between ***D*-BPAM** and methyl acrylate (catalyzed by **3-*M***).

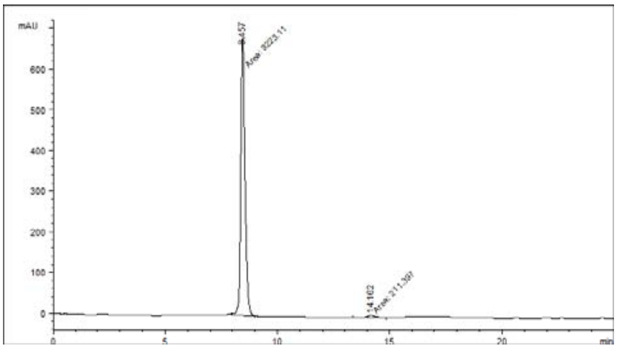

Peak #	RetTime [min]	Area [mAU *s]	Height [mAU]	Area [%]
1	8.457	9223.109	685.570	97.759
2	14.162	211.397	6.492	2.241
Total	—	9434.507	692.062	100.000

Figure S64. HPLC trace of the product from the cycloaddition reaction between ***D*-BPAM** and methyl acrylate (catalyzed by **3-*P***).

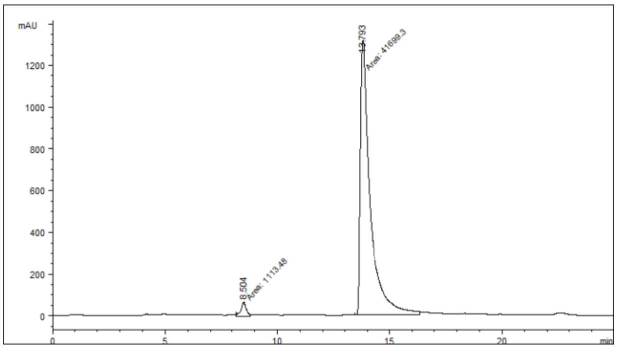

Peak #	RetTime [min]	Area [mAU *s]	Height [mAU]	Area [%]
1	8.504	1113.476	69.102	2.601
2	13.793	41699.266	1347.262	97.399
Total	—	42812.742	1416.363	100.000

Figure S65. HPLC traces of the products from five parallel tandem catalytic systems of **2+*L*-BPAM** (entries a-e in Table S11).

(a)

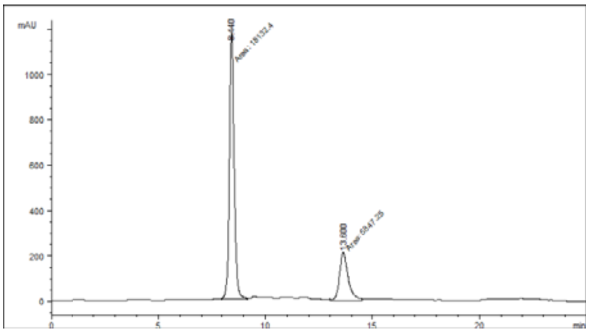

Peak #	RetTime [min]	Area [mAU *s]	Height [mAU]	Area [%]
1	8.440	18132.391	1174.235	75.616
2	13.600	5847.249	212.049	24.384

Total	–	23979.640	1386.284	100.000

(b)

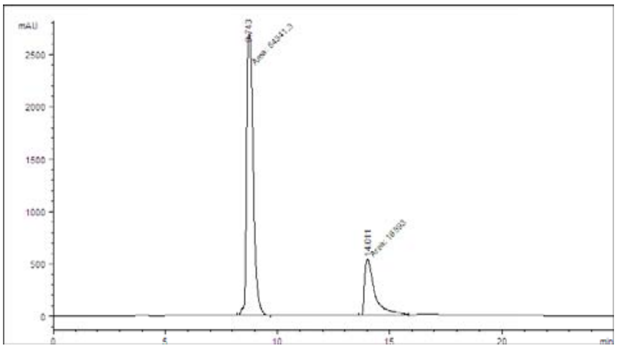

Peak #	RetTime [min]	Area [mAU *s]	Height [mAU]	Area [%]
1	8.743	54341.324	2685.315	76.607
2	14.011	16592.988	534.350	23.393
Total	–	70934.312	3219.665	100.000

(c)

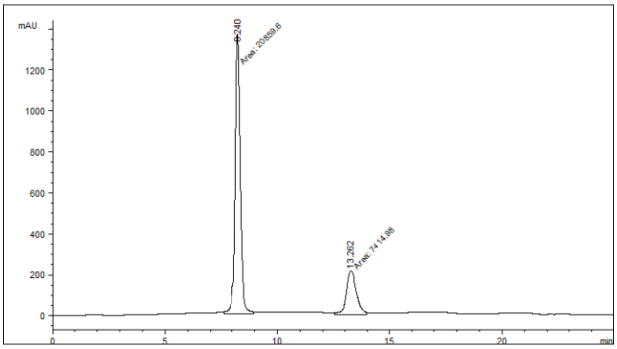

Peak #	RetTime [min]	Area [mAU *s]	Height [mAU]	Area [%]
1	8.240	20859.619	1373.198	73.775
2	13.262	7414.980	222.580	26.225
Total	–	28274.599	1595.778	100.000

(d)

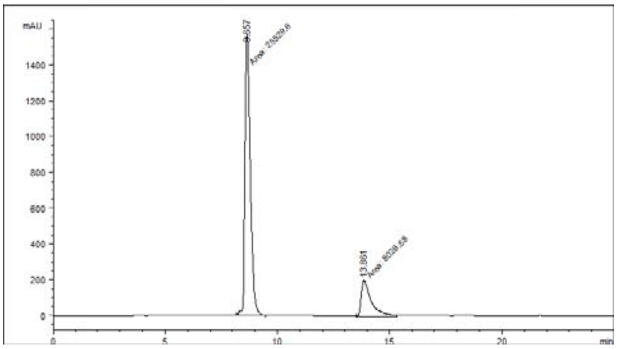

Peak #	RetTime [min]	Area [mAU *s]	Height [mAU]	Area [%]
1	8.657	25529.590	1571.436	76.080
2	13.861	8026.575	215.820	23.920
Total	–	33556.165	1787.256	100.000

(e)

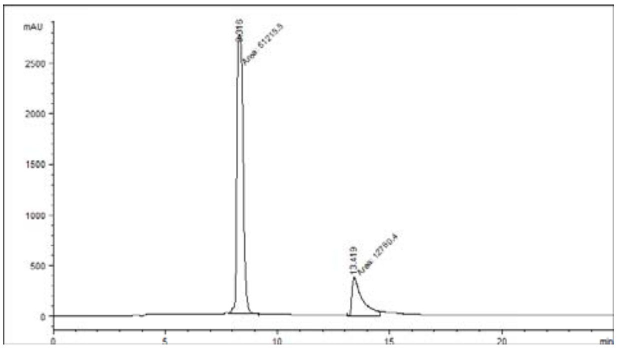

Peak #	RetTime [min]	Area [mAU *s]	Height [mAU]	Area [%]
1	8.316	51215.535	2755.052	80.054
2	13.419	12760.384	382.854	19.946
Total	—	63975.919	3137.906	100.000

Figure S66. HPLC traces of the products from five parallel tandem catalytic systems of **3+*L*-BPAM** (entries f-j in Table S11).

(f)

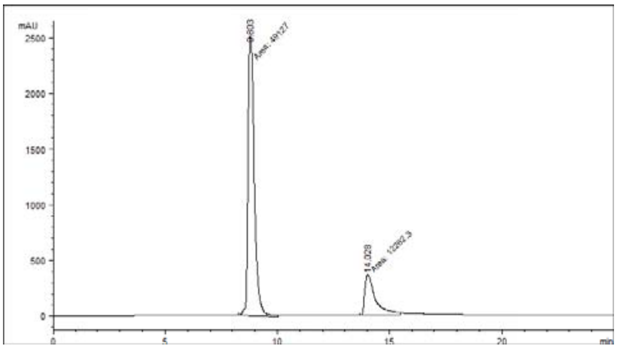

Peak #	RetTime [min]	Area [mAU *s]	Height [mAU]	Area [%]
1	8.803	49126.965	2529.323	80.025
2	14.028	12262.342	363.609	19.975
Total	—	61389.307	2892.932	100.000

(g)

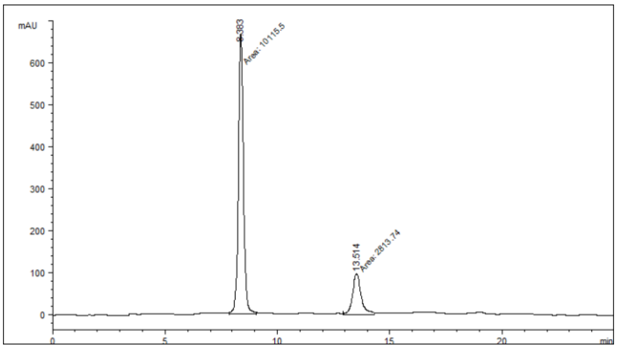

Peak #	RetTime [min]	Area [mAU *s]	Height [mAU]	Area [%]
1	8.383	10115.515	669.809	78.237

2	13.514	2813.735	97.878	21.763
Total	—	12929.250	767.687	100.000

(h)

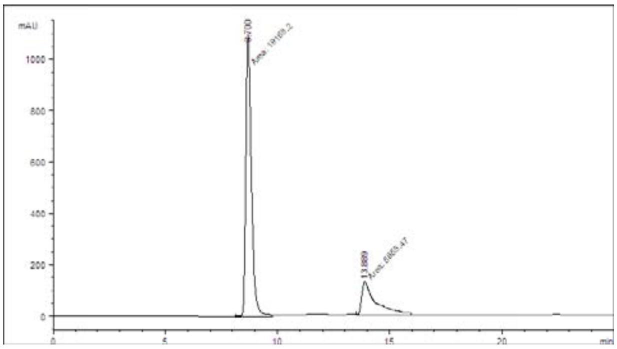

Peak #	RetTime [min]	Area [mAU *s]	Height [mAU]	Area [%]
1	8.700	19168.175	1100.271	77.217
2	13.889	5655.466	130.599	22.783
Total	—	24823.641	1230.870	100.000

(i)

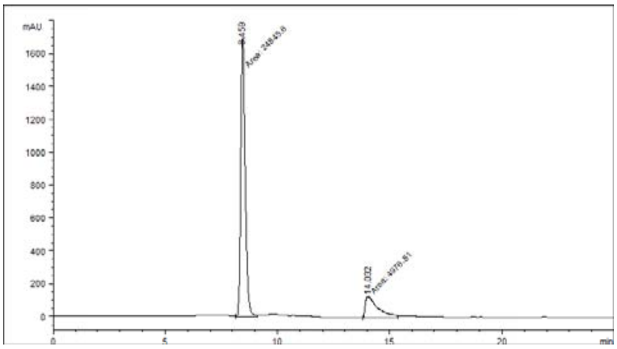

Peak #	RetTime [min]	Area [mAU *s]	Height [mAU]	Area [%]
1	8.459	24845.627	1708.910	83.312
2	14.032	4976.813	130.507	16.688
Total	—	29822.439	1839.417	100.000

(j)

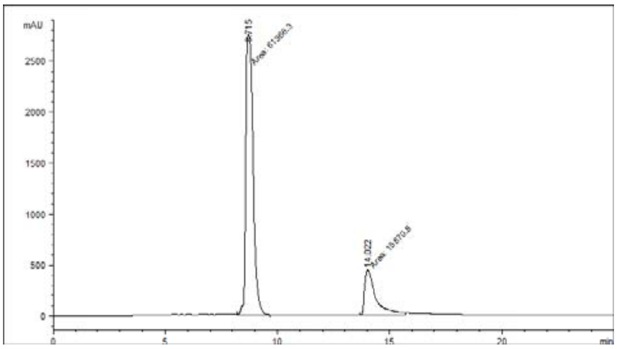

Peak #	RetTime [min]	Area [mAU *s]	Height [mAU]	Area [%]
1	8.715	61366.270	2756.679	79.452
2	14.022	15870.824	445.675	20.548

Total	—	77237.094	3202.355	100.000

Figure S67. HPLC traces of the products from five parallel tandem catalytic systems of **2+*D*-BPAM** (entries a-e in Table S12).

(a)

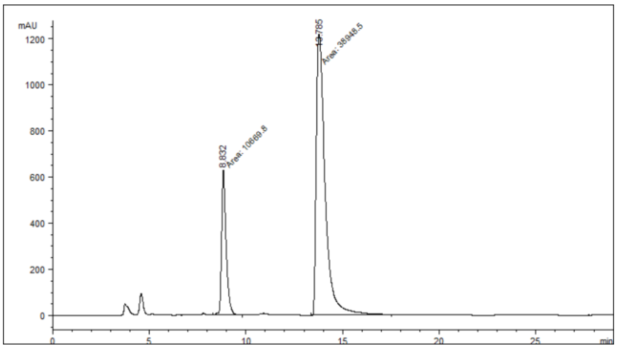

Peak #	RetTime [min]	Area [mAU *s]	Height [mAU]	Area [%]
1	8.832	10669.752	629.071	21.504
2	13.785	38948.531	1220.065	78.496
Total	—	49618.283	1849.136	100.000

(b)

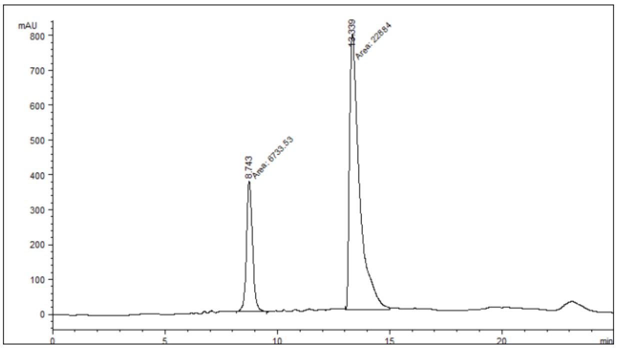

Peak #	RetTime [min]	Area [mAU *s]	Height [mAU]	Area [%]
1	8.743	6733.534	375.276	22.735
2	13.339	22883.965	793.149	77.265
Total	—	29617.499	1168.425	100.000

(c)

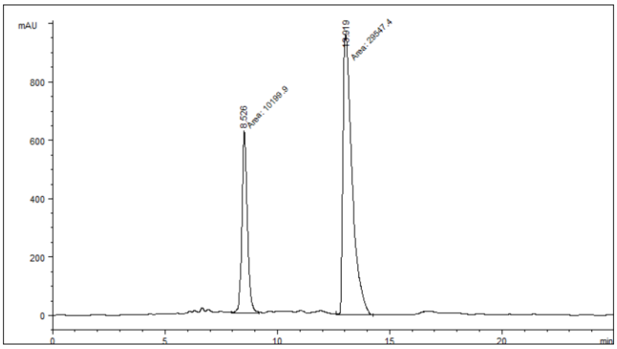

Peak #	RetTime [min]	Area [mAU *s]	Height [mAU]	Area [%]

1	8.526	10199.854	620.344	25.662
2	13.919	29547.391	955.376	74.338
Total	—	39747.245	1575.72	100.000

(d)

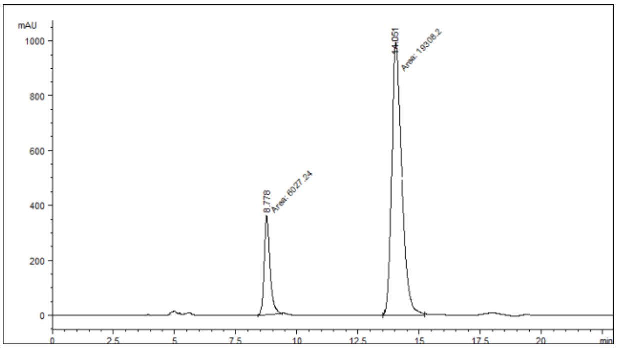

Peak #	RetTime [min]	Area [mAU *s]	Height [mAU]	Area [%]
1	8.778	6027.241	360.991	23.789
2	14.051	19308.236	999.753	76.211
Total	—	25335.477	1360.744	100.000

(e)

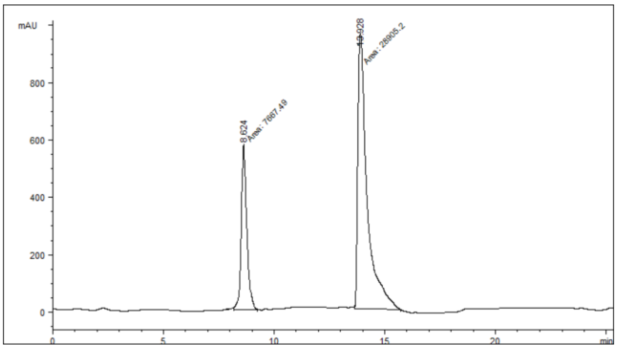

Peak #	RetTime [min]	Area [mAU *s]	Height [mAU]	Area [%]
1	8.624	7667.491	587.227	20.965
2	13.928	28905.211	958.994	79.035
Total	—	36572.702	1546.221	100.000

Figure S68. HPLC traces of the products from five parallel tandem catalytic systems of **3+*D*-BPAM** (entries f-j in Table S12).

(f)

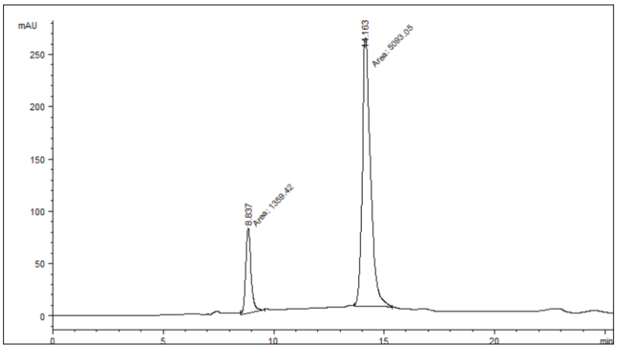

Peak #	RetTime [min]	Area [mAU *s]	Height [mAU]	Area [%]
1	8.837	1359.422	81.592	21.068
2	14.163	5093.047	195.991	79.035
Total	—	6452.469	277.583	100.000

(g)

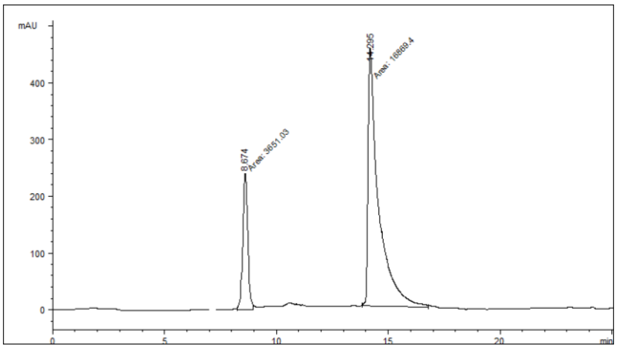

Peak #	RetTime [min]	Area [mAU *s]	Height [mAU]	Area [%]
1	8.674	3651.026	241.806	17.792
2	14.295	16869.402	457.655	82.208
Total	—	20520.428	699.461	100.000

(h)

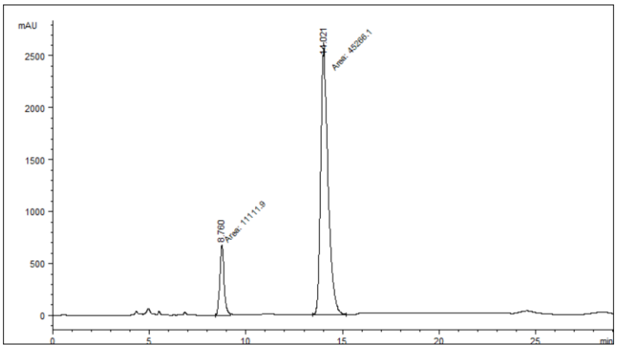

Peak #	RetTime [min]	Area [mAU *s]	Height [mAU]	Area [%]
1	8.760	11111.895	686.331	19.709
2	14.021	45266.111	2614.807	80.291
Total	—	56378.006	3301.138	100.000

(i)

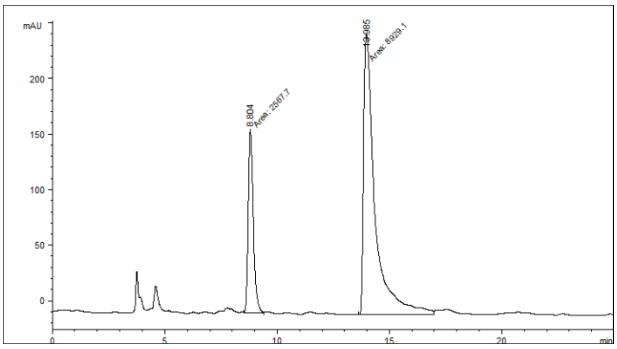

Peak #	RetTime [min]	Area [mAU *s]	Height [mAU]	Area [%]

1	8.804	2567.699	164.892	22.334
2	13.985	8929.095	252.481	77.666
Total	—	11496.794	417.373	100.000

(j)

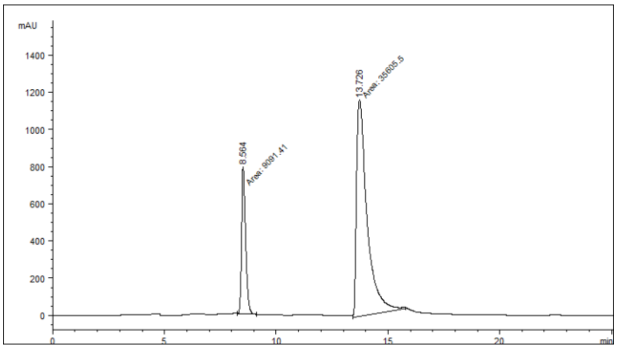

Peak #	RetTime [min]	Area [mAU *s]	Height [mAU]	Area [%]
1	8.564	9091.414	974.751	20.341
2	13.726	35605.508	1150.716	79.659
Total	—	44696.922	2125.467	100.000

Supplementary References

1. Lai, G. F., Bu, X. R., Santos, J. & Mintz, E. A. Reinvestigation of the Vilsmeier-Haack formylation of triphenylamine. *Synlett* **1997,** 1275–1276 (1997).
2. Thomas, G. L. *et al.* Anti-MRSA agent discovery using diversity-oriented synthesis. *Angew. Chem. Int. Ed.* **47,** 2808–2812 (2008).
3. Yamashita, Y.; Imaizumi, T. & Kobayashi, S. Chiral silver amide catalyst for the [3+2] cycloaddition of α-amino esters to olefins. *Angew. Chem. Int. Ed.* **50,** 4893–4896 (2011).
4. Longmire J. M., Wang, B. & Zhang, X. Highly enantioselective Ag(I)-catalyzed [3+2] cycloaddition of azomethine ylides. *J. Am. Chem. Soc.* **124,** 13400-13401 (2002).
5. Tsubogo, T., Saito, S., Seki, K., Yamashita, Y. & Kobayashi, S. Development of catalytic asymmetric 1,4-addition and [3+2] cycloaddition reactions using chiral calcium complexes. *J. Am. Chem. Soc.* **130,** 13321-13332 (2008).
6. Boruah, M.; Konwar, D. & Sharma, S. D. KF/Al_2O_3 mediated 1,3-dipolar cycloaddition of azomethine ylides: a novel and convenient procedure for the synthesis of highly substituted pyrrolidines. *Tetrahedron Lett.* **48,** 4535–4537 (2007).
7. SAINT, Data reduction software (version 6.45) (Bruker AXS Inc., Madison, WI, 2003).
8. Sheldrick, G. M. SADABS Empirical Absorption Correction Program (University of Göttingen, 1996).
9. Sheldrick, G. M. *SHELXTL Reference Manual*: *Version 5.1* (Bruker AXS Inc., Madison, WI, 1997).

10 Sheldrick, G. M. *SHELXL-97: Program for Crystal Structure Refinement* (University of Göttingen, 1997).
